# Spatiotemporal variations in hydrometeorological drought across North Africa and indications of anthropogenic amplification

**DOI:** 10.1038/s43247-026-03807-2

**Published:** 2026-07-13

**Authors:** Morteza Rahimpour, Taha B. M. J. Ouarda, Emna Gargouri-Ellouze, Fairouz Slama

**Affiliations:** 1 Canada Research Chair in Statistical Hydro-Climatology, Institut National de la Recherche Scientifique, Centre Eau Terre Environnement, INRS-ETE, Québec, QC Canada; 2 University of Tunis El Manar, National Engineering School of TUNIS (ENIT), LR99ES19; Laboratory of Modelling in Hydraulics and Environment (LMHE), Tunis, Tunisia

**Keywords:** Environmental impact, Natural hazards, Hydrology

## Abstract

Conventional unidimensional approaches that monitor only a single type of drought in water-resources research, particularly in the context of emerging droughts, often lead to ineffective planning and management strategies. This study addresses that gap by examining multiple large-scale drought types through a streamlined, integrative framework for monitoring severe drought events driven by both climate change and human activities across North Africa (NA), a region characterized by pronounced data scarcity and observation gaps. The spatiotemporal analysis of hydro-meteorological drought variations across NA demonstrates that anthropogenic factors seem to have substantially intensified hydrological drought conditions throughout the region. Furthermore, between 2021 and 2024, the area experienced unprecedented meteorological droughts. Evaluation of multiple independent datasets shows that traditional univariate monitoring frameworks significantly underestimate the magnitude and persistence of extreme events, such as the 2010 NA drought, because they fail to capture the multidimensional and interactive nature of drought dynamics. These results highlight the necessity of adopting a multivariate, spatiotemporally integrated perspective to accurately assess extreme droughts, even where observational data remain limited. In Tunisia, the findings reveal that hydrological drought patterns diverge notably from meteorological trends: despite relatively stable precipitation between 2002 and 2010, groundwater storage declined sharply after 2010–2011. This divergence underscores the potential influence of anthropogenic stressors, particularly unsustainable groundwater abstraction, in amplifying and prolonging hydrological drought beyond the effects of climatic variability alone. Nevertheless, this relationship warrants further investigation. Overall, these insights hold critical implications for improving drought-monitoring systems and advancing sustainable water resources management across NA.

## Abstract (web summary)

North Africa has experienced a persistent drying trend since 1998 culminating in unprecedented drought conditions in 2024, potentially amplified by anthropogenic contributions, according to a comparative data-driven approach integrating satellite data, global datasets and observations.

## Introduction

Traditionally, drought is defined at a given location as the moisture deficit over a specific period compared to a long-term statistical period at the same location, and it can be strictly attributed to meteorological phenomenon for monitoring purposes^1,2^. However, as a complex natural hazard, it encompasses various dimensions, with concepts and definitions that differ depending on the context of the study^2–6^.

In general, any drought event can be categorized and explained based on its context and the variable under investigation into four categories: (1) Meteorological drought (precipitation, temperature, etc.), (2) Agricultural drought (soil moisture, vegetation indicators, etc.), (3) Hydrological drought (surface water discharge and/or groundwater storage, etc.), and (4) Socioeconomic drought (economic, social, ecological, and/or health impacts associated with drought or related categories)^7,8^. Given the interdisciplinary nature of drought studies, extensive global research has focused on analyzing and modeling the processes, impacts, and characteristics of one or more drought events, such as their spatiotemporal extent, intensity, duration, and frequency^9–17^.

The examination of droughts over time in North Africa (NA) reveals that this part of the African continent has experienced a rapid shift toward drier conditions in recent decades^18,19^. These continuous and prolonged changes have led to numerous adverse consequences^20,21^. Among the most significant of these is the destruction of a substantial volume of surface water resources^22^. Nevertheless, while the impact of drought on groundwater resources in NA is undoubtedly severe, it remains shrouded in uncertainty due to the lack of access to observational data^23^.

However, numerous independent studies have been conducted in various countries, including Morocco, Algeria, Tunisia, Libya, and Egypt, focusing on aspects of drought other than anthropogenic drought^13,15,24–28^. All these studies emphasize that the primary limitation in studying drought in this region is the lack of access to observational data. One notable example is the study by Tanarhte, et al.^29^, which provides comprehensive insights through a review of drivers, impacts, and management in NA. Their research revealed that, over the past fifty years, the 1999–2004 drought period was the most widespread and significant for all NA countries except Libya (including Morocco, Algeria, Tunisia, and Egypt). Their findings also indicated that the most recent impactful drought occurred during 2015–2016. It is worth mentioning that these droughts were extremely severe and could be classified not only as meteorological droughts but also in other categories, such as agricultural and socioeconomic droughts. This classification is supported by evidence showing that cereal production in Algeria, Morocco, and Tunisia significantly declined during these events^29^, with further impacts on national budgets, export revenues, and import costs^29^. Moreover, the extent of drought relative to territorial area affected Tunisia and Morocco more than other NA countries^30^. While, the assessments by Abdelmalek and Nouiri^25^, based on data from 16 main meteorological stations in Tunisia between September 1973 and August 2016, revealed that Tunisia experienced a prolonged drought (approx., six years) from the late 1970s to the early 1980s, with 1981 being the most difficult year. However, their results indicated that drought recurred in this country during the 2000s to 2010s, with shorter and less intense durations (approx., three years), the most severe of which occurred in 2001.

While numerous studies have examined individual types of droughts or focused on localized case studies, few have simultaneously and comprehensively assessed the spatiotemporal evolution of both meteorological and hydrological droughts across NA^18–21,29,30^. To address this gap, the present study aims to assess the spatiotemporal dynamics of drought variations using a comparative, data-driven, and streamlined approach that integrates publicly available satellite observations and global datasets in NA. Where severe data scarcity and limited ground-based observations pose major challenges to conventional drought assessment^29^. This approach enables the detection of non-stationary behavior and the identification of statistically significant change points in long-term time series of key meteorological and hydrological variables, including surface and groundwater resources. Importantly, the methodology allows for the investigation of coupled human–water–climate interactions without reliance on government-restricted data sources. The analysis is structured around four central research questions: (1) What are the spatiotemporal trends in key meteorological and hydrological variables across the five North African countries? (2) Are statistically significant change points present that indicate shifts in time series behavior? (3) If so, when and where have these shifts occurred? and (4) To what extent is hydrological drought a direct consequence of meteorological drought, and where might anthropogenic drivers explain any decoupling between them?

## Results and discussions

### Monitoring meteorological drought

#### Detection of changes in meteorological variables

**Fig. 1:**
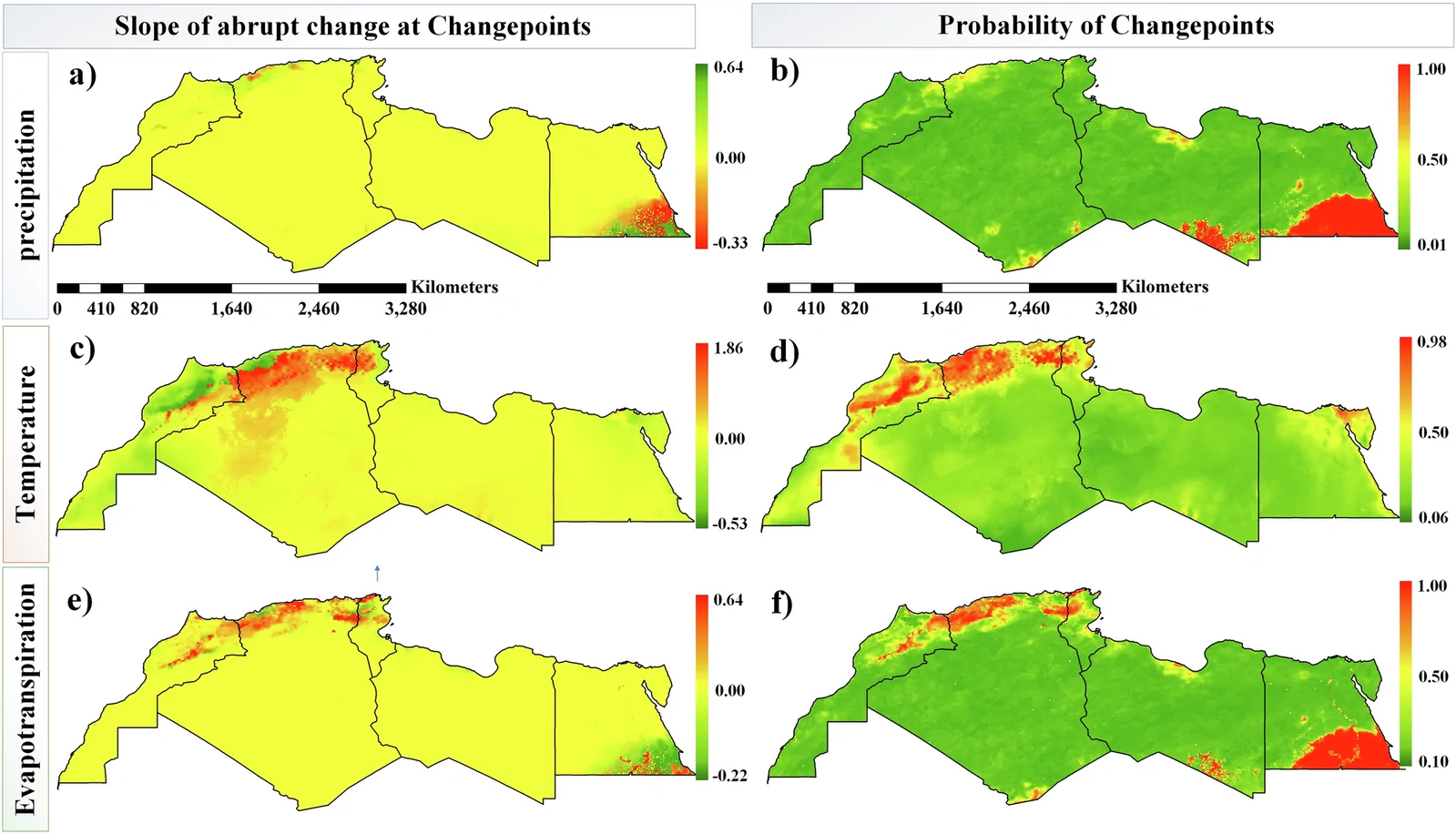
Spatiotemporal trends and abrupt changes in hydroclimatic variables across North Africa. Results of the spatiotemporal time-series analysis based on the ERA5 dataset for 1981–2024. Panels **a** and **b** show the slope and probability of abrupt changes for precipitation, panels **c** and **d** show the slope and probability of abrupt changes for temperature, and panels **e** and **f** show the slope and probability of abrupt changes for evapotranspiration.

Figure 1 illustrates the spatial changes in the probability of abrupt changes and the slope of variations derived from the time series analysis of three variables: temperature, precipitation, and evaporation; for all pixels across the five studied countries using the ERA5 dataset for the period 1981–2024. In all maps depicting these three variables (Fig. 1), the color legend is standardized so that red corresponds to the most pronounced transitions toward drier conditions. As can be inferred from comparing all maps in Fig. 1, the most significant and widespread changes in time series behavior across the region are associated with temperature, followed by evaporation and precipitation. Figure 1a, 1b shows that changes in precipitation are limited to the southern regions of Egypt. These Fig. 1e, 1f, shown evapotranspiration in southern Egypt exhibits a heterogeneous pattern, with both positive and negative slope changes across adjacent pixels. Nevertheless, the number of pixels displaying decreasing evapotranspiration coinciding with increasing precipitation appears greater, suggesting localized hydroclimatic recovery in some parts of the region. Comparison of the maps in Fig. 1a, 1b reveals that no significant change points are observed in the precipitation time series across a broad spatial extent encompassing a large number of pixels within the studied countries.

The temperature maps (Fig. 1c,  1d) show that significant changes are observed across extensive areas of the Atlas Mountains region of NA near the Mediterranean Sea, covering parts of Tunisia, Algeria, and Morocco. In Fig. 1c, the slope values of temperature changes are positive, exceeding 1.5 in red-highlighted regions, indicating increasing temperature trends in these areas. Figure 1e,  1f depict changes in evapotranspiration during the study period. A comparison of the map in Fig. 1e with the other maps (Fig. 1a, 1c) suggests that the time series changes for the evapotranspiration variable are closely linked to both precipitation and temperature variables, with both contributing significantly to the increasing evapotranspiration trends. Figure 1e indicates the slope changes in northern regions in the Atlas Mountains are positive, exhibiting behavior similar to the positive trends observed in the temperature time series. Additionally, positive slope changes in evapotranspiration are observed in southern Egypt at critical breakpoints, particularly in areas where increasing precipitation trends have been detected. The rise in temperature, combined with increased evapotranspiration, has led to higher water demands for crops, indicating that more water resources are needed for agriculture compared to the past. Overall, the spatiotemporal analysis highlights substantial changes across various regions of NA, characterized by increasing temperature and evapotranspiration and decreasing precipitation near the Mediterranean coasts.

**Fig. 2:**
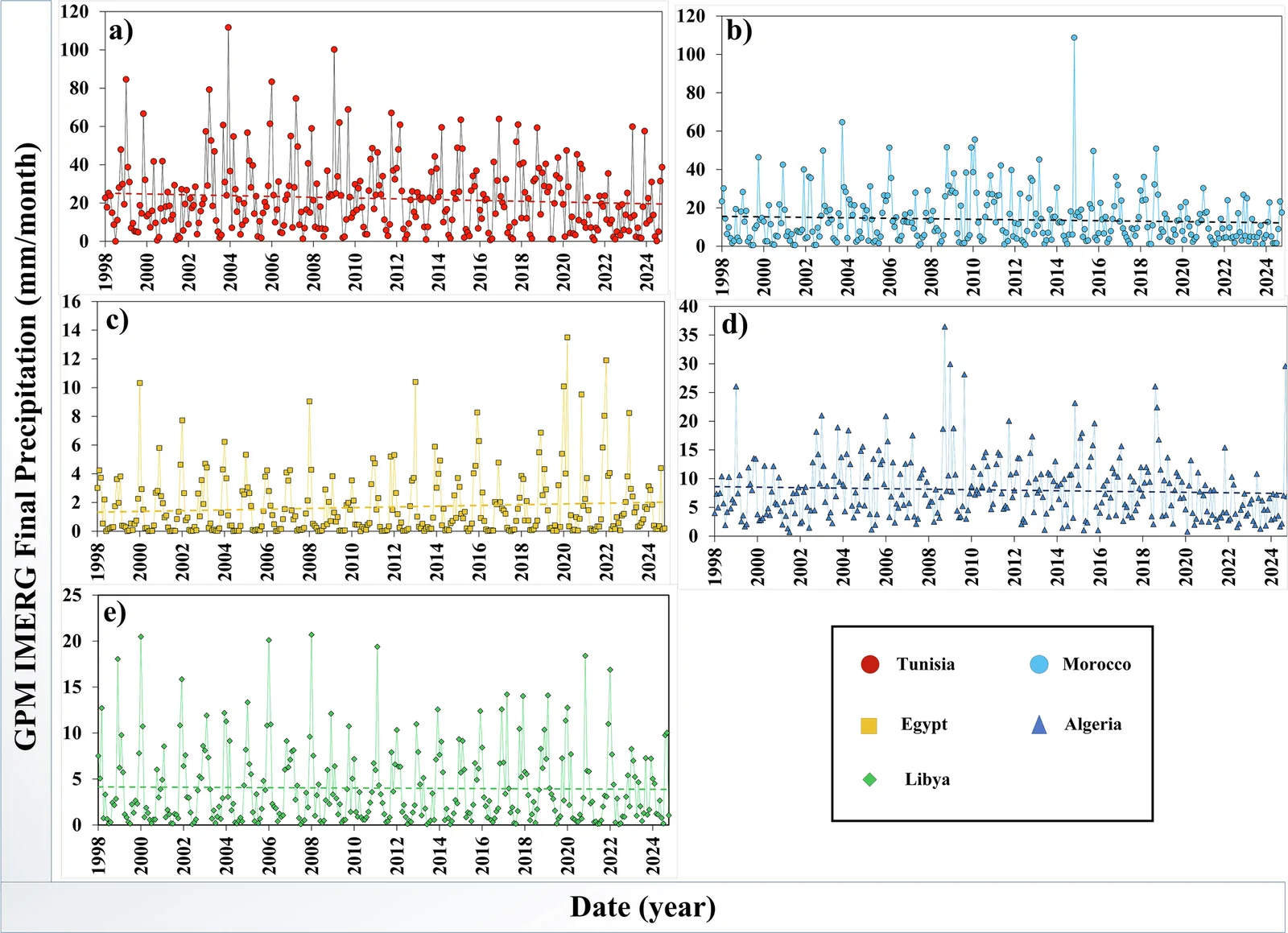
Monthly precipitation variability across five North African countries. Monthly precipitation time series derived from the GPM IMERG product for 1998–2024 across five countries: **a** Tunisia; **b** Morocco; **c** Egypt; **d** Algeria; and **e** Libya.

It is noteworthy that the breakpoints detected in a few pixels in Fig. 1 may not be spatially extensive enough to achieve statistical significance at broader scales, such as the national level. For example, as shown in Fig. 1a, precipitation exhibits localized significant breakpoints in parts of Egypt and Algeria; however, this does not necessarily imply corresponding, statistically significant changes in the drought indices for those areas. To address these spatial limitations and the paucity of in situ observations, we drew on multiple open data sources, including satellite-based precipitation datasets (Fig. 2). Figure 2 presents the monthly precipitation time series for each of the five countries. As indicated, no significant trend except light visually trend discernible isn’t apparent, and both the BEAST and MMK tests confirm the absence of statistically significant breakpoints or changepoints in any precipitation series. By contrast, as discussed in the following section, the drought indices reveal distinct long-term patterns, including statistically significant trends and breakpoints, that provide deeper insight into the region’s hydroclimatic variability.

These findings are consistent with previous studies that did not identify substantial precipitation declines or statistically significant breakpoints across NA, yet documented episodes of severe drought, particularly during 1998–2001^19,29–31^. Accordingly, the next section examines drought behavior using multiple indices derived from diverse datasets. Moreover, unlike earlier research that focused primarily on meteorological indicators, our analysis integrates satellite-based groundwater storage variations with conventional drought metrics, providing a more comprehensive perspective to potentially link climatic variability with anthropogenic influences.

#### Meteorological drought index results

**Fig. 3:**
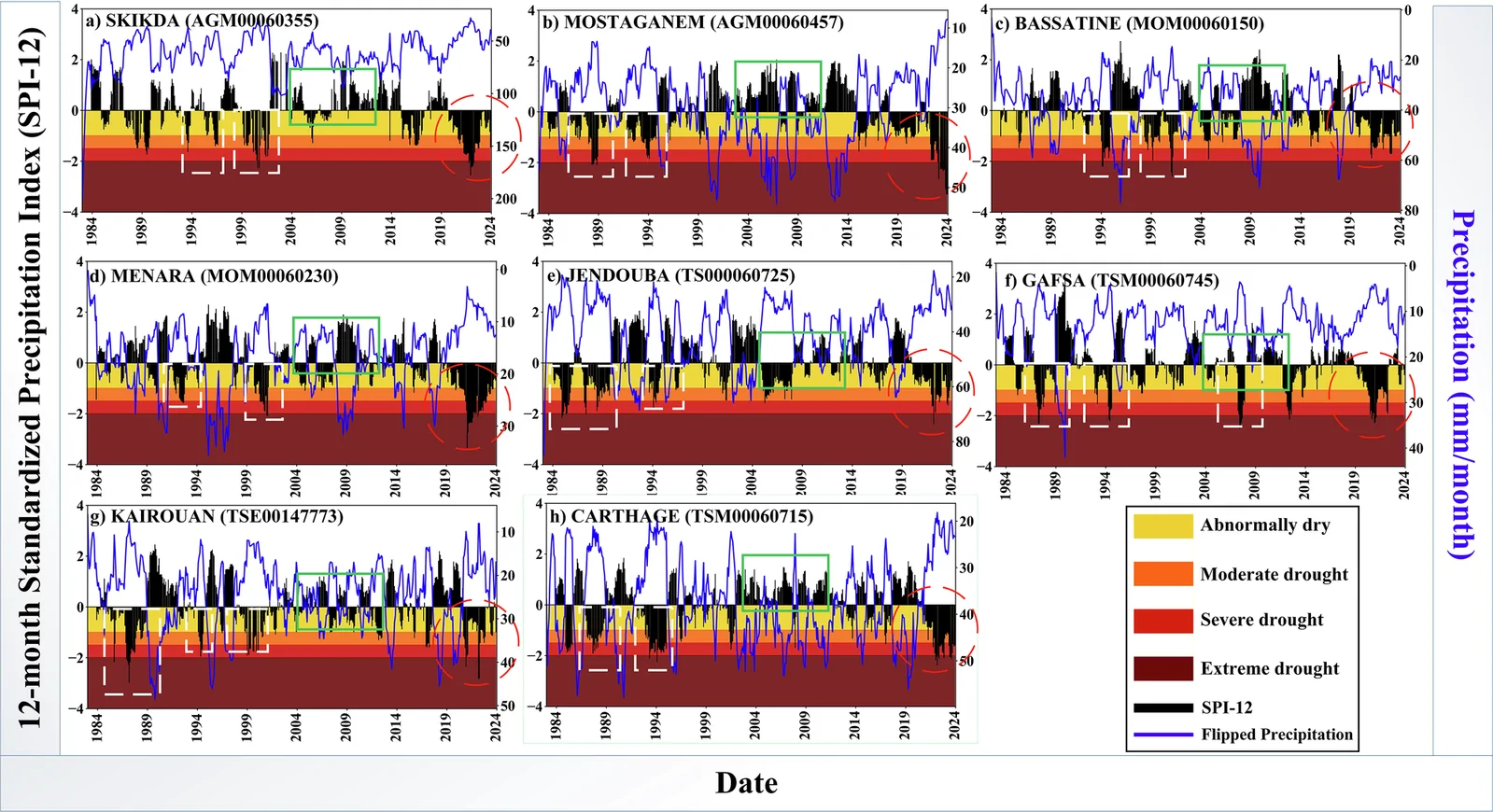
Drought-index and precipitation variability at eight selected stations across North Africa. Twelve-month Standardized Precipitation Index values (SPI-12; black bars) and precipitation totals (blue line) are shown for the observation stations during 1984–2024: **a** SKIKDA (AGM00060355); **b** MOSTAGANEM (AGM00060457); **c** BASSATINE (MOM00060150); **d** MENARA (MOM00060230); **e** JENDOUBA (TS000060725); **f** GAFSA (TSM00060745); **g** KAIROUAN (TSE00147773); and **h** CARTHAGE (TSM00060715).

In Fig. 3, the variations of the 12-month SPI (SPI-12) and precipitation are illustrated for eight representative observational stations across NA, selected as a sample from all available stations (refer to the Supplementary Files for additional details). Results indicate that, aside from a few instances of weak and short-term droughts, nearly all moderate to severe and prolonged drought events exhibit a consistent pattern across the analyzed stations. Figure 3 highlights that during the late 1980s, early 1990s, and late 1990s, Algeria, Tunisia, and Morocco experienced regionally coherent droughts (white dashed rectangles), which have also been reported in previous studies^18,20,29,30^. Furthermore, Fig. 3 indicates that from 2002 to 2011, precipitation levels at all stations were above the long-term mean, although some regions still experienced mild drought conditions (green rectangles in Fig. 3). Most notably, a historically unprecedented drought has emerged in recent years, clearly visible across all stations. This severe drought began in 2019 and has intensified each year since (red dashed circle in Fig. 3).

**Fig. 4:**
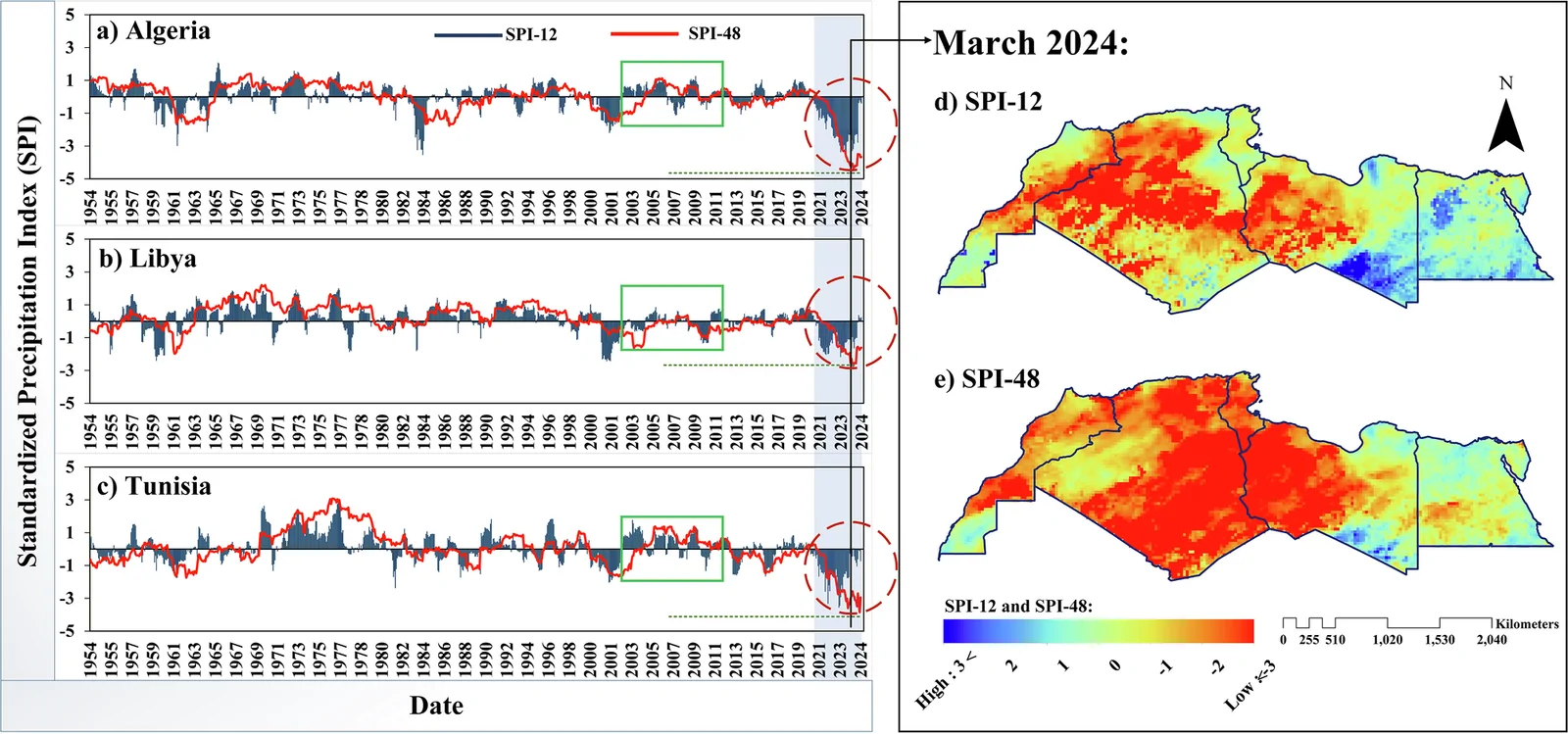
Temporal drought variability and spatial drought conditions across North Africa. Time-series variations of SPI-12 and SPI-48 based on ERA5 data at the national scale for Algeria, Libya, and Tunisia are shown in panels **a**, **b**, and **c**, respectively. Spatial distributions of SPI-12 and SPI-48 across North Africa for March 2024 are shown in panels **d** and **e**, respectively.

This temporal behavior is corroborated by ERA5 reanalysis data (Fig. 4). Figure 4a–c presents the national-scale time series of SPI-12 and SPI-48 derived from ERA5 for Algeria, Libya, and Tunisia, respectively, while Fig. 4d–e displays the spatial distribution of both indices across North Africa for March 2024. The national-scale results (Fig. 4a–c) confirm the occurrence of intense and unprecedented droughts during 2021–2024 (red dashed circles) and near-normal conditions between 2001 and 2010 (green rectangles). Because ERA5 products are partly constrained by GHCN ground-based observations, their temporal behavior closely aligns with the station-based SPI results (Fig. 3 and Supplementary Material). As shown in Fig. 4d–e, the selected accumulation period (or timescale) plays a critical role in interpreting spatial SPI results and must be evaluated jointly, as it can substantially alter the spatial classification of drought severity even for the same date. For instance, in Tunisia, the SPI-12 map for March 2024 (Fig. 4d) classifies the country as experiencing moderate drought, whereas the SPI-48 map (Fig. 4e) categorizes nearly the entire territory under extreme drought, consistent with widespread aridity across NA. This comparison underscores the necessity of analyzing multiple accumulation periods simultaneously to capture both short- and long-term drought dynamics. Also, integrating multiple data sources is likewise essential for a comprehensive understanding of the spatial and temporal variability of drought conditions across complex climatic regimes.

**Fig. 5:**
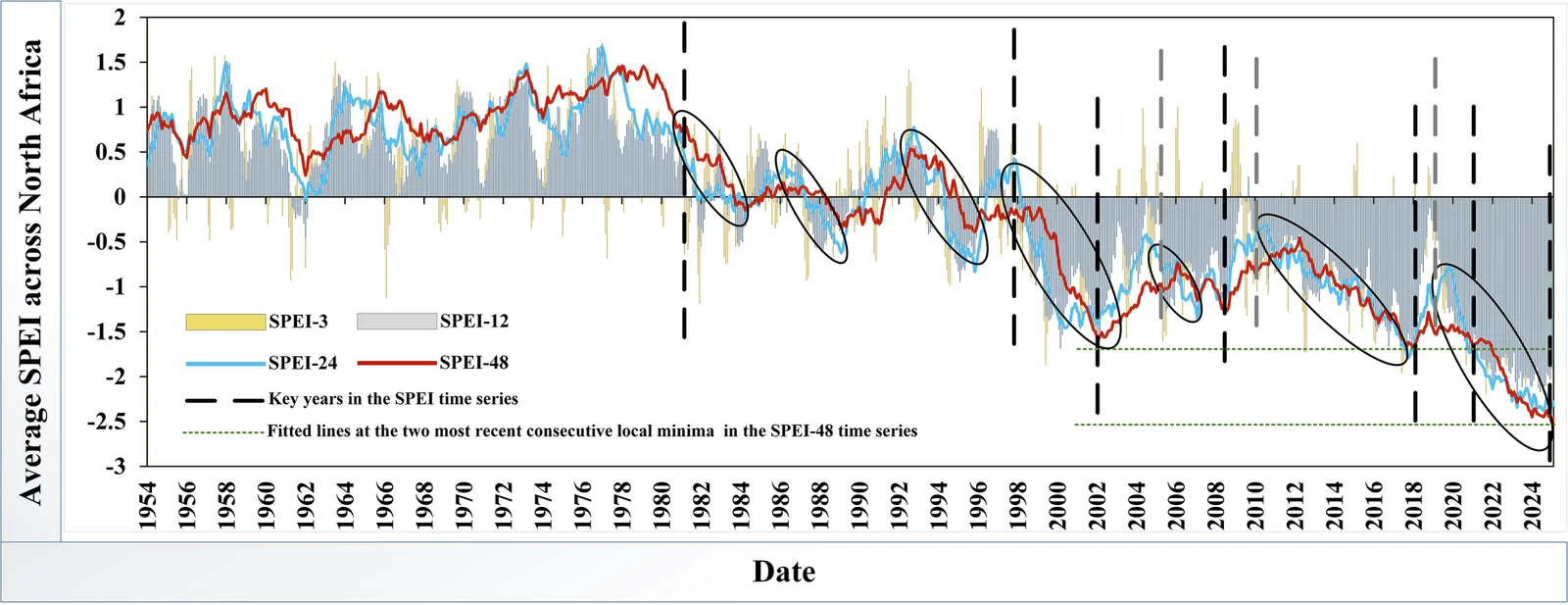
Average SPEIbase time series over the North Africa for 3-, 12-, 24-, and 48-month timescales (1954–2024).

To assess drought conditions more comprehensively, the SPEI, which incorporates both precipitation and potential evapotranspiration from external different sources, was also utilized. The temporal changes in SPEI were examined across four timescales (3, 12, 24, and 48 months), averaged over the three countries based SPEIbase dataset, and are illustrated in Fig. 5. The results reveal that the temporal patterns of the SPEI (Fig. 5) closely mirror those of the SPI observed in all stations (Fig. 3) and ERA5 drought index (Fig. 4). However, some variations in timing and magnitude between the two indices (SPI and SPEI) are expected due to differences in accumulation periods and calculation methodologies. Figure 5 shows that over the past 60 years, North Africa has experienced seven dry periods (black ovals), among which three stand out due to their exceptional severity, duration, and intensity. The first major drought occurred from 1998 to 2004, and the second from 2010 to 2018. Both were comparable in overall severity, though the first exhibited higher intensity, while the second lasted longer.

The third and most critical drought began in early 2019 and is still ongoing. As shown in Figs. 3, 4 and 5, this drought intensified dramatically from 2021 onward, breaking historical records across multiple dimensions. This ongoing event is notable for three key reasons: (1) Record-breaking: This drought surpasses all previous events in terms of severity, intensity, and duration. Its severity since 2021 is unprecedented over the past six decades. Figure 3 (e.g., parts a, b, etc.), Figs. 4 and  5 illustrate that its intensity rivals the 1998–2004 drought, but it has already surpassed it in duration. Compared to the 2010–2018 drought, it is more intense and severe. (2) Widespread extent: The drought spans a vast region, particularly affecting three countries, Tunisia, Algeria, and Morocco; in a synchronized pattern. This broad extent aligns with regional temperature and precipitation anomalies (see Fig. 1). (3) Compounding with long-term drying: This drought has emerged amid a prolonged multi-decadal drying trend, which has weakened the resilience of ecosystems, agricultural systems, and water supplies. As a result, its consequences are expected to be especially profound, likely exceeding the impacts of previous droughts across hydrological, agricultural, economic, and social sectors that were reported before^29^.

In summary, based on the results presented in this section (Figs. 3, 4 and 5), the onset of severe and prolonged droughts in North Africa can be traced back to the mid-1990s. Since 1998, the region has experienced sustained drying. During this period, three major droughts have been identified, with the current ongoing drought, beginning in 2019 and reaching record-breaking severity in 2021, representing the most critical in terms of spatial extent and historical intensity.

### Monitoring hydrological drought

The pixel-by-pixel time-series analysis revealed notable changes in meteorological variables across localized regions in several North African countries, including Tunisia, Algeria, Morocco, and Egypt. However, at the national scale, no significant changes or breakpoints were detected (Fig. 2). The drought monitoring assessment further characterized the timing, duration, and severity of drought events across North Africa. Together, these results indicate that meteorological drought variations are evident across the region and can alter time-series behavior at the pixel level. Nevertheless, these changes are not spatially extensive enough to produce detectable shifts at the country scale, including in Tunisia. This localized behavior is likely driven by broader climatic changes in the Mediterranean region^31^ that have intensified and deepened droughts in specific areas, as highlighted by the red pixels in Fig. 1.

However, according to the literature, numerous but scattered studies have reported a sharp decline in both the quality and quantity of groundwater resources in certain countries (e.g., Abdelmalek and Nouiri^25^^,^Frija, et al.^32^ and Hamdi, et al.^11^). To identify the underlying causes of these severe and widespread changes, it became necessary to analyze drought at a deeper level, beyond meteorological drought and general climate change, which have already been addressed in terms of reduced precipitation and rising temperatures. It was also essential to explore the relationship between meteorological and hydrological droughts and to address the critical question: Is hydrological drought a direct consequence of meteorological drought? If no, why?

#### Terrestrial water storage anomalies (TWSA) decomposition based on GRACE/GRACE-FO time series

**Fig. 6:**
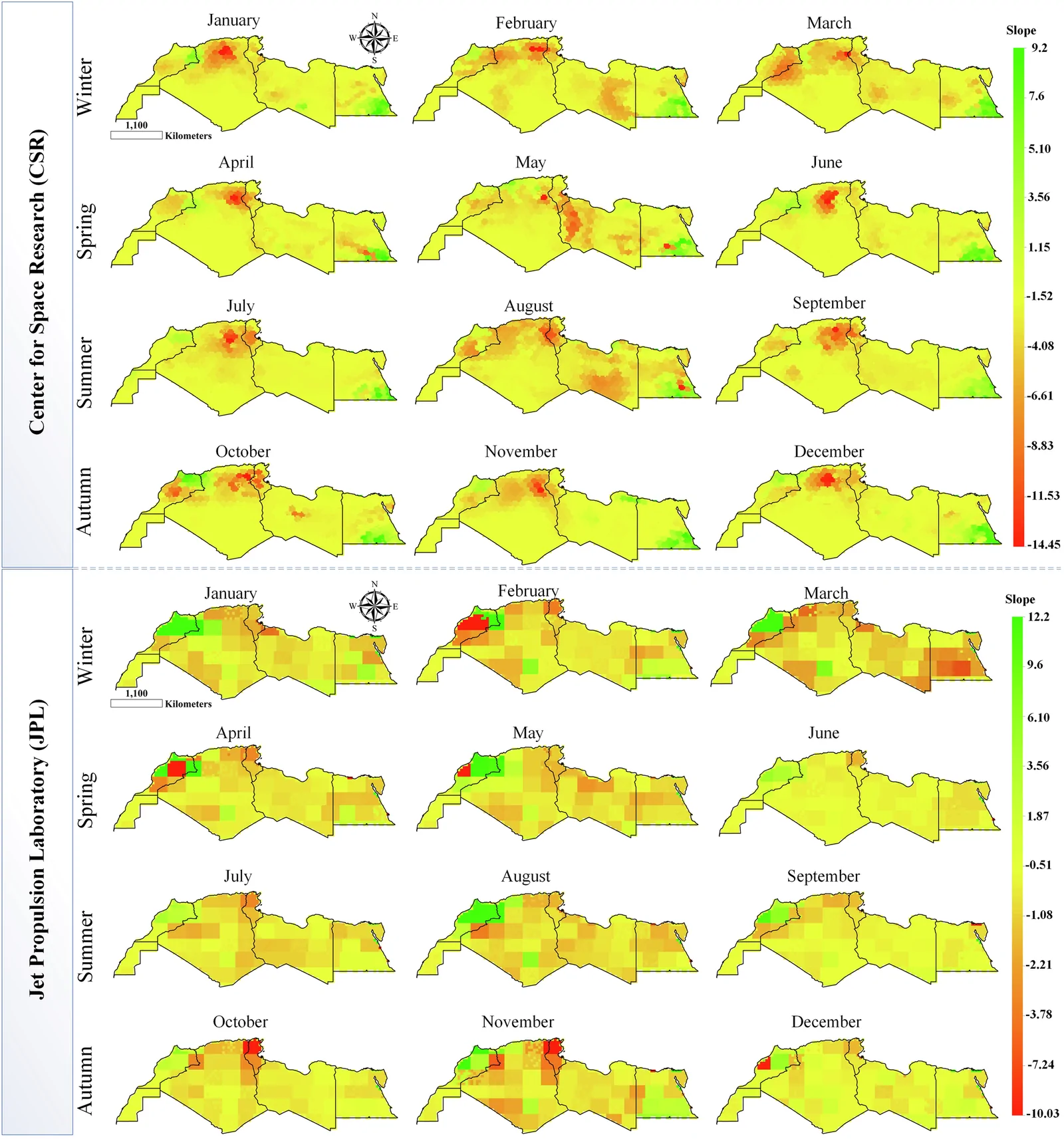
Spatiotemporal distribution of the slopes corresponding to the points of maximum probability of abrupt change in the monthly total water storage (TWS) time series, based on CSR and JPL GRACE datasets for the period 2002–2024.

Figure 6 displays the slope of changes at points where breakpoints in the TWSA time series derived from the BEAST analysis are statistically significant, using two products, CSR and JPL, on a monthly basis. As shown in Fig. 6, the two products exhibit different time series behaviors. The CSR product demonstrates almost consistent behavior across all months, with major decreases observed over a broad area along the border between Tunisia and Algeria. However, the TWSA time series analysis using the JPL product indicates that the time series behavior varies across pixels over different months. Specifically, Fig. 6 illustrates that the JPL product in October and November reveals the most significant negative slope changes at breakpoints occurring in Tunisia, which are highly significant (see Fig. S2). However, due to the coarse resolution of these products, they are suitable only for early warning of groundwater drought, a limitation also noted in other studies^33,34^. Furthermore, Figs. 6 and  S2 illustrate that the CSR and JPL products yield different results, and averaging their outputs to create a new product is not advisable.

#### Groundwater and drought monitoring

Table 1 presents the trend changes using the MK test and Sen’s Slope for each of these three indicators across the five studied countries. As shown in the table, the most significant negative trends are observed in Tunisia, Algeria, and Morocco, respectively. However, a more important observation is that Sen’s Slope increases as the depth of soil moisture analysis moves from surface to deeper layers. This means that as the analysis shifts to greater soil depths, the decreasing trends become more pronounced, and the likelihood of the trends being statistically significant increases. This indicates that drought effects are more pronounced at greater depths, closer to groundwater, where human activities, such as groundwater extraction, have a larger impact.

**Table 1:** Trend analysis results: bold values indicate trends significant at 95% and 99% confidence levels across five countries in North Africa

Variables	Country Name	Sen’ slope	Direction	Significant at 5%	Significant at 1%
Surface soil moisture percentile (SFSM)	Egypt	–0.00675	Decreasing	FALSE	FALSE
	Morocco	–0.34094	Decreasing	TRUE	TRUE
	Algeria	–1.75262	Decreasing	TRUE	TRUE
	Libya	–1.98698	Decreasing	TRUE	TRUE
	Tunisia	–2.03501	Decreasing	TRUE	TRUE
Root zone soil moisture percentile (RZSM)	Egipt	–0.18387	Decreasing	TRUE	FALSE
	Morocco	–0.3635	Decreasing	TRUE	TRUE
	Algria	–2.0268	Decreasing	TRUE	TRUE
	Libya	–2.21761	Decreasing	TRUE	TRUE
	Tunisia	–2.27731	Decreasing	TRUE	TRUE
Groundwater storage percentile (GWS)	Egipt	–0.23679	Decreasing	TRUE	TRUE
	Morocco	–0.57408	Decreasing	TRUE	TRUE
	Libya	–1.90643	Decreasing	TRUE	TRUE
	Algria	–2.15695	Decreasing	TRUE	TRUE
	Tunisia	–2.91261	Decreasing	TRUE	TRUE

**Fig. 7:**
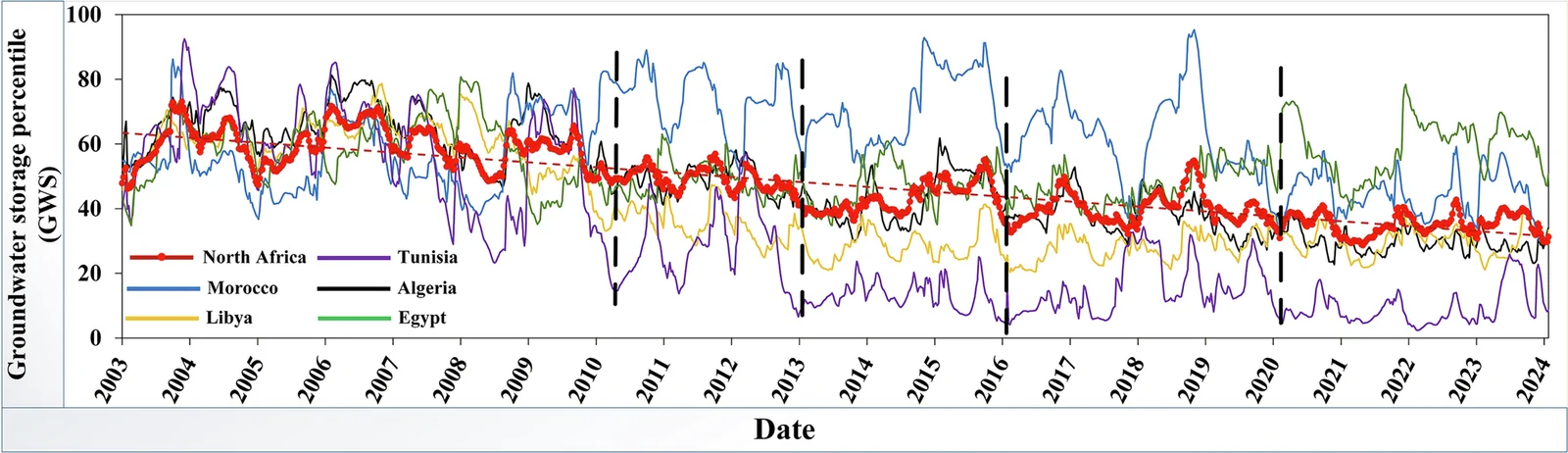
Time series of groundwater storage percentiles for the five individual countries and the area-averaged value across all countries (in red) in North Africa (2003–2024).

Figure 7 presents the groundwater storage percentile changes for each of the five countries individually, as well as for the entire North African region. The figure indicates that the overall trend of Groundwater storage across North Africa is declining. However, the time series behavior varies notably between countries. A comparison of all the plots in Fig. 7 reveals that groundwater storage in Morocco and Egypt remains relatively stable, with some years even showing values above the long-term average. In contrast, Algeria and Libya exhibit groundwater storage changes that align closely with the regional mean. Among all five countries, Tunisia stands out with the most significant reduction in groundwater storage. As illustrated by the purple curve in Fig. 7, groundwater levels in Tunisia have declined dramatically, following a trend that is clearly distinct from the others. This indicates that hydrological drought in Tunisia is more severe than in other North African countries.

**Fig. 8:**
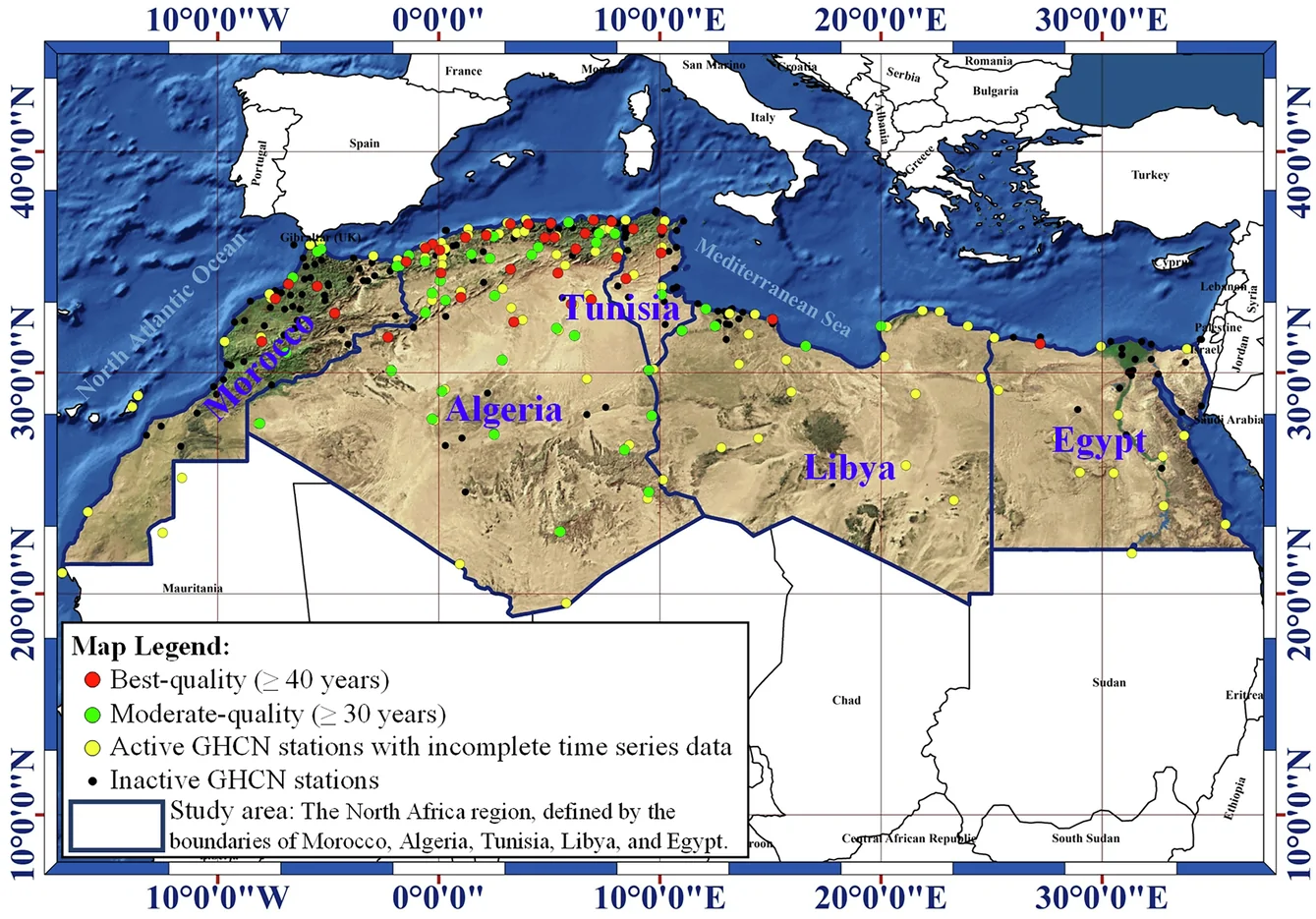
Study area: five countries in North Africa.

Several factors may account for this discrepancy. The first concerns differences in spatial scale, which are critical in explaining the contrasting drought behaviors observed in Tunisia relative to other North African countries. As illustrated in Fig. 8, Tunisia is geographically smaller than its neighbors, such as Libya and Algeria. Consequently, droughts driven by climatic variability in the Mediterranean region often encompass the entire Tunisian territory, whereas only portions of the much larger Algeria, Morocco, or Libya are affected. However, comparison of Figs. 6 and  7 with the plots presented earlier (Figs. 2, 3, and 4) clearly indicates that meteorological drought alone cannot account for the hydrological drought observed in Tunisia. Between 2002 and 2010, precipitation remained relatively favorable (Fig. 2a), and drought conditions were largely normal (green rectangles in Figs. 3e–h and 4c). Nevertheless, a distinct breakpoint and sharp decline in groundwater storage appear around 2010–2011 (purple curve in Fig. 6). These findings suggest that the hydrological drought in Tunisia cannot be attributed solely to meteorological drivers; rather, anthropogenic factors likely played a significant role in intensifying this pronounced decline.

### Amplified drought linked to anthropogenic influences

This section explores the implications of the results to better understand the potential role of anthropogenic factors in the occurrence of amplified drought in North Africa, with a particular focus on developments in Tunisia compared to other studies. Additionally, by addressing the study’s limitations and identifying existing knowledge gaps, detailed opportunities for future research are presented. The findings indicate notable changes in the time series trends of meteorological variables within localized areas of limited spatial extent. These changes may be attributed to drought or climate change, as suggested in previous studies on NA^29,31^. However, our analysis of drought impacts on groundwater resources offers an additional perspective.

The results show that while meteorological variables exhibit relatively consistent trends across North African countries, the reduction in groundwater resources due to hydrological drought is more severe in certain nations. Among the five countries studied, Tunisia displays the most significant negative trends despite sharing broadly similar hydroclimatic conditions with its neighbors. This appears to be the primary factor contributing to the widespread decline in both the quality and quantity of groundwater resources, a concern frequently highlighted in recent research^35–37^. The time series analysis of TWSA and GRACE-DA-DM at the country scale revealed a steep and statistically significant downward trend in Tunisia’s groundwater resources. Given that agriculture represents the main livelihood for a large portion of the population, this decline poses a direct threat to national well-being^38–41^.

Overall, the findings of this study are consistent with previous research, confirming that Tunisia’s water resources have experienced a dramatic decline, particularly since 2010-2011. This decline is attributed to poor groundwater governance compounded by overlapping seasonal droughts. The severe and irreversible depletion of groundwater resources threatens to trigger salinization, land subsidence, and severe impacts on the agricultural sector, and further deterioration of water quality^23,42,43^. These challenges, particularly in the aftermath of the unprecedented emerging drought (2021–2024) documented here, are expected to have profound and far-reaching consequences across all sectors of society. Marginalized farmers, whose livelihoods depend directly on groundwater availability, are likely to be the most severely affected^41^.

Time series analysis also identified a statistically significant breakpoint in 2010, accompanied by a negative trend that cannot be fully explained by meteorological variables. Although drought occurred during this period, our findings indicate that climatic drivers alone do not account for the extent and severity of Tunisia’s groundwater depletion. Other studies, such as those by Abdelmalek and Nouiri^25^ and Tanarhte, et al.^29^, which specifically examined drought in Tunisia, suggest that even more severe meteorological droughts occurred in earlier years (e.g., 1981 and 2001). This discrepancy supports the hypothesis of a more intense drought event, termed amplified drought, whose primary driver is anthropogenic influence.

Therefore, all available evidence indicates that, apart from increased consumption and excessive groundwater abstraction, no other factor could have played a significant role in intensifying the hydrological drought in Tunisia. However, the mechanisms through which these processes influence water-use dynamics, as well as the underlying drivers of their occurrence, remain unknown.

### Uncertainty and limitations

In this study, each dataset and drought index was analyzed independently using its native temporal coverage and a dataset-specific reference period at either the national or regional scale. This methodological choice was deliberately adopted to avoid discarding valuable long-term information that would otherwise be lost if all analyses were constrained to a single, shorter common period across heterogeneous data sources. Consequently, the temporal behavior of each country’s time series was examined over the full available record of the corresponding dataset. This strategy enabled robust identification of country-specific temporal patterns, breakpoints, and inter-country similarities and differences without relying on direct comparisons of absolute index magnitudes. While this approach minimizes information loss and enhances the detection of long-term variability and non-stationarity, it also introduces uncertainty related to differences in dataset-specific reference periods, which is acknowledged as an inherent limitation of multi-source drought analyses.

Importantly, this strategy does not compromise the robustness of the main conclusions. Despite differences in native temporal coverage, a sufficiently long common overlapping period spanning 2002–2024 exists across all major datasets employed in this study. This shared temporal window encompasses the most critical hydroclimatic features discussed, including the pronounced post-2010 shifts and the unprecedented emerging drought observed between 2021 and 2024. It therefore provides a robust and independent basis for cross-validation of temporal coherence across datasets. Moreover, interpretations are derived primarily from relative temporal dynamics and trend behavior rather than absolute magnitudes, thereby reducing sensitivity to source-specific biases and measurement uncertainties.

Furthermore, the analyses combined large-scale spatial averaging with pixel-wise statistical evaluations, each of which introduces distinct analytical constraints. In particular, pixel-level changepoint detection across large spatial domains may generate statistical uncertainties and spatial artifacts, especially in regions characterized by heterogeneous hydroclimatic conditions. These limitations were anticipated and are explicitly acknowledged within the uncertainty framework of this study.

The spatiotemporal hydrometeorological drought results reveal pronounced changes after 2010, suggesting potential links between drought intensification and anthropogenic drivers such as groundwater abstraction. However, despite the emphasis on anthropogenic influences, this study is strictly confined to hydrological and hydroclimatic evidence. Accordingly, these interpretations should be approached with caution. The limitations of the present research highlight the need for future studies to adopt integrated socio-hydrological approaches that examine groundwater governance, land-use change, and water consumption, particularly agricultural water use, in Tunisia, where the most pronounced post-2010 anomalies were observed.

An additional limitation concerns the interpretation of localized versus regional-scale drought signals. Meteorological drought variability is analyzed exclusively using the SPEIbase dataset, and all conclusions referring to widespread or sustained drying are derived from spatially aggregated SPEI fields rather than from individual stations or localized anomalies. While local-scale variability is intrinsic to drought processes across North Africa, the regional interpretations presented here are based on statistically coherent patterns observed at aggregated spatial scales, thereby reducing the risk of extrapolating isolated signals to the regional domain. Nevertheless, spatial aggregation may partially obscure subregional heterogeneity, and caution is therefore warranted when interpreting regional-scale summaries in areas characterized by strong climatic and physiographic gradients. To enhance transparency and enable independent assessment of spatial variability, the raw SPEIbase data used in this analysis have been made publicly available (see Supplementary Files).

## Conclusion

In this study, unlike conventional approaches, spatiotemporal variations in hydrometeorological drought were analyzed to detect significant change points and assess abrupt changes, both anthropogenic and climatic, in amplifying drought across North Africa (NA). This region, characterized by severe data scarcity, observational gaps, and limitations in documenting human water withdrawals, was investigated using a simplified yet robust framework that relies on limited in-situ observations while integrating openly accessible satellite datasets and global public databases. The results indicate that since 1998, the entire NA region has experienced a persistent drying trend, culminating in unprecedented drought conditions in 2024. The analyses further reveal indications of anthropogenic contributions that may have amplified drought severity, particularly after 2010–2011. The findings suggest that in recent years, especially since 2021, an exceptionally severe drought has emerged and persisted through 2024, representing an event without precedent over the past six decades. Within this context, Tunisia, Algeria, and Morocco have been the most critically affected countries, both in terms of spatial extent and drought severity, experiencing conditions ranging from critical to extreme. Furthermore, the evaluation of hydrological drought indices confirms that these three countries face significant water stress. However, the patterns of hydrological drought indicators exhibit distinct behaviors across countries. In Tunisia, for example, the spatiotemporal evolution of hydrological indicators does not align with meteorological drought trends. The results show a statistically significant decline in hydrological variables, particularly groundwater storage after 2010–2011. This contrasts with the period from 2002 to 2010, during which precipitation levels remained relatively stable and no severe meteorological drought was detected. Despite this apparent meteorological stability, a pronounced hydrological drought emerged after 2010, accompanied by a sharp decline in Tunisia’s groundwater resources. This temporal decoupling suggests that additional non-meteorological factors may have contributed to the observed groundwater depletion. While excessive groundwater abstraction represents a plausible mechanism consistent with the timing and spatial coherence of the decline, formal causal attribution would require dedicated socio-hydrological data beyond the scope of the present study. Nevertheless, the critical question remains whether anthropogenic changes have contributed to the amplification of drought. Additionally, the analysis demonstrates that hydrological drought has affected the broader North African region, primarily driven by the unprecedented meteorological drought from 2021 to 2024. The interaction between extreme climatic conditions, human activities, and heterogeneous water management strategies across countries may generate diverse socio-economic and environmental consequences. Each of these consequences warrants independent investigation in future research, particularly to understand why and how such severe transformations occurred in Tunisia after 2010–2011. Based on these findings, it is strongly recommended that policymakers and experts in the affected countries implement urgent and strategic measures to mitigate the impacts of these persistent droughts. Given their extensive repercussions, especially for hydrology, water resources, agriculture, and the broader economy, proactive and adaptive management strategies are essential to enhance resilience against ongoing and potentially intensifying drought events.

## Material and methods

### Data: observation, global datasets and remote sensing

The study area is characterized by a pronounced scarcity of ground-based observational data. To address this limitation and ensure a comprehensive evaluation, a broad suite of global databases encompassing the maximum available temporal coverage was employed. As a result, uniform baseline periods were not imposed across all datasets, since doing so would have excluded valuable temporal information. Given the objectives of this research, defining a fixed reference period was unnecessary, as the primary aim was to detect major fluctuations and assess the non-stationarity of spatiotemporal hydro-meteorological drought variations through a comparative framework, rather than to quantify absolute variable magnitudes. Accordingly, multiple datasets with varying temporal spans were integrated into the analysis. As summarized in Table 2, the “period used” in this study may differ from the “period available” originally provided by each data source.

**Table 2:** Overview of all data used in this study, including variables, data type, dataset name, available period, spatial resolution, and references

Variable/drought index	Type	Dataset name	Period available	Analysis periods	Spatial resolution	Reference
Actual Evapotranspiration (ETa)	reanalysis dataset	ERA5-Land	1950-present	1981–2024	0.1^o^	^57^
Mean surface air temperature (at 2-meter)	reanalysis dataset	ERA5-Land			0.1^o^	^57^
Precipitation (P)	reanalysis dataset	ERA5-Land			0.1^o^	^57^
	The Integrated Multi-satellitE Retrievals for GPM (IMERG) is the unified U.S. algorithm that provides the multi-satellite precipitation product for the U.S. GPM team.	GPM_3IMERGM v7	1998–2024	1998–2024	0.1^o^	^58^
Terrestrial water storage (TWS)	RS (GRACE and GRACE-FO)	JPL	2002- present	2002- present	0.5^o^	^59^
	RS (GRACE and GRACE-FO)	CSR			0.25^o^	^60^
Standardised Precipitation-Evapotranspiration Index (SPEI)	near real-time dataset	SPEIbase	1950-present	1954–2024	0.5^o^	^48,61^
Surface soil moisture percentile (SFSM)	GRACE Data Assimilation for Drought Monitoring	GRACEDADM-SFSM	2003-present	2003-present	0.25^o^	^46^
Root zone soil moisture percentile (RTZSM)		GRACEDADM-RTZSM				
Groundwater storage percentile (GWS)		GRACEDADM-GWS				

In the present study, the latest version (Version 3) of the Global Historical Climatology Network (GHCN) monthly precipitation dataset^44^ was used to calculate and investigate SPI-12 across NA (see metadata in Table S1 and Supplementary Files). This dataset consists of historical observational records from ground-based stations, including a total of 280 stations distributed throughout the study area, as shown in Fig. 8 and Table S1. Among these, 131 stations remained active through 2024. Of these, 76 meteorological stations with observation records exceeding 30 years were selected for the drought analysis conducted in this study (Fig. 8, red and green). Stations shown in red represent those with long-term, high-quality time series extending beyond 40 years (1984–2024).

Due to the limited spatial coverage and uneven distribution of GHCN stations across North Africa, no spatial interpolation of the station time series was performed. Instead, all analyses were conducted on a point-to-grid basis. GHCN station data were used to compute the SPI and to perform independent trend analyses of drought variability based on in situ precipitation records. Importantly, the temporal patterns detected in station-based SPI series are consistent with those derived from gridded products, thereby providing independent observational support for the main findings. Comprehensive plots showing the trend, seasonal, and residual components for all 131 active stations (identified by station name), as well as SPI time series for 35 selected stations (indicated by red points in Fig. 8), are provided in the Supplementary Files.

Special attention is given to satellite-based data products from the Gravity Recovery and Climate Experiment (GRACE) and its successor, GRACE Follow-On (GRACE-FO), launched in March 2002 and May 2018, respectively. These missions are particularly valuable for monitoring groundwater and climate-related changes, serving as integrated indicators of anthropogenic effects on water resources^45^. The primary GRACE-derived products used in this study are the Total Water Storage (TWS) anomalies generated by the Jet Propulsion Laboratory (JPL) and the Center for Space Research (CSR). In addition, three satellite-based drought indicators, groundwater storage (GWS), root-zone soil moisture (RTZSM), and surface soil moisture (SFSM); were incorporated from NASA’s GRACE Data Assimilation for Drought Monitoring (GRACE-DA-DM) product^46,47^, which provides global dryness or wetness percentiles based on historical records from 1948 to 2014The Standardized Precipitation Evapotranspiration Index (SPEI) was employed as a climate-based drought index to capture temporal variability across multiple timescales (1 to 48 months). Monthly SPEI data were retrieved from the SPEIbase repository, a globally consistent, near-real-time monitoring system^48,49^.

### Study area

North Africa constitutes the northernmost region of the African continent, situated above the Sahara Desert and bordered by the Mediterranean Sea to the north. Although its exact boundaries vary across definitions, the region is generally considered to extend from the Atlantic coast of Morocco in the west to Egypt and the Red Sea coast of Sudan in the east. In this study, NA refers specifically to five countries, Morocco, Algeria, Tunisia, Libya, and Egypt, which collectively define the study domain (Fig. 8). These countries share strong geographic proximity, comparable climatic and hydrological regimes, and similar demographic, political, social, cultural, and economic characteristics^29^.

The climate of NA is predominantly Mediterranean along the narrow coastal belts of Morocco, Algeria, Tunisia, and parts of Libya, where the mean annual precipitation ranges between 400- and 600-mm yr⁻¹. Inland regions exhibit semi-arid climates (200–400 mm yr⁻¹) that gradually transition to arid desert zones receiving less than 100 mm yr⁻¹ of rainfall^20,29,50^. Three major physiographic features govern the regional climate system: the Mediterranean Sea to the north, the Sahara Desert to the south, and the Atlas Mountains to the west. From a geomorphology the Atlas Mountains extend across northern Morocco, Algeria, and Tunisia, reaching their highest elevation in central Morocco^50^. From a limnological and hydrological perspective, the region exhibits distinctive characteristics. The hydrology of NA is defined by several major river systems that sustain human and ecological life in an otherwise dry and semi-arid environment. The most important of these include the Nile, Medjerda, Moulouya, Oued Draa, and Oum Er-Rbia rivers, each playing a crucial role in supporting agriculture, urban settlements, and ecosystems across Egypt, Sudan, Tunisia, Algeria, and Morocco^29,50^. The Nile River, the longest river in the world, forms the backbone of Egypt’s economy and food security, while smaller rivers such as the Medjerda in Tunisia and the Moulouya in Morocco remain vital to local economies and regional irrigation networks^50^.

The total area of the study domain covers approximately 5.8 million km² and hosts about 223.6 million inhabitants, equivalent to roughly 2.8 % of the global population. A substantial share of this population resides in rural areas, where livelihoods depend directly on agriculture and water availability^29,50^. Consequently, variations in climate and the occurrence of droughts have immediate implications for water accessibility, agricultural productivity, and socioeconomic stability. Understanding spatiotemporal hydro-meteorological drought variations across this region is therefore essential, as such analyses not only clarify drought dynamics but also provide critical insight into broader challenges related to agriculture, water security, and societal resilience. However, this endeavor is constrained by limited observational networks, data scarcity, and restricted data accessibility, which collectively hinder regional climate and hydrological assessment. These limitations underscore the necessity of the present research, which seeks to analyze hydro-meteorological drought events in a region where livelihoods and food security remain tightly interlinked with climate variability and water-resource availability.

### Drought indicators: SPI and SPEI

To quantify meteorological drought conditions, two standardized drought indices were employed: the Standardized Precipitation Index (SPI) and the Standardized Precipitation Evapotranspiration Index (SPEI). These indices are based on probabilistic standardization of climate variables and allow for multi-temporal analysis of drought intensity and duration.

The SPI, proposed by McKee, et al.^51^, is based solely on precipitation and is used to characterize precipitation anomalies over various accumulation periods. Monthly precipitation series are first aggregated over the desired time scale (e.g., 1, 3, 6, or 12 months). The aggregated precipitation values are then fitted to a probability distribution, typically the two-parameter gamma distribution:

1 $$f(x)=\frac{{x}^{\alpha -1}{e}^{x/\beta }}{{\beta }^{\alpha }\varGamma (\alpha )},{{\rm{x}}} > 0$$

where α and β are the shape and scale parameters, respectively. The gamma function Ӷ(α) is defined as:

2 $$\varGamma (\alpha )=\int _{0}^{\infty }{y}^{\alpha -1}{e}^{-y}dy$$

SPEI extends SPI by incorporating the climatic water balance D_i_ = P_i_ - PET_i_^48^, where P is the monthly precipitation and PET is the potential evapotranspiration (e.g., via Hargreaves metho). A log-logistic distribution is fitted to the series of D_i_:

3 $$f(D)=\frac{\beta '}{\alpha '}{\left(\frac{D-\gamma }{\alpha '}\right)}^{\beta '-1}{\left[1+{\left(\frac{D-\gamma }{\alpha '}\right)}^{\beta '}\right]}^{-2}$$

Parameters α‘, β‘ and γ are estimated using L-moments or probability-weighted moments. The probability distribution is:

4 $$F(D)={\left[1+{\left(\frac{\alpha '}{D-\gamma }\right)}^{\beta '}\right]}^{-1}$$

### Trend analysis and Spatiotemporal Change Point Detection

#### Bayesian time-series decomposition using BEAST

We used the BEAST algorithm (Bayesian Estimator of Abrupt change, Seasonal change, and Trend), implemented in the Rbeast package^52,53^, to detect changepoints and decompose drought-related time series into trend and seasonal components. Bayesian data analysis is a particularly adapted framework for changepoint detection, time-series decomposition, and the analysis of trends in the decomposed series (e.g. Seidou and Ouarda^54^). BEAST is a Bayesian ensemble model that employs Bayesian Model Averaging (BMA) and Markov Chain Monte Carlo (MCMC) to quantify uncertainty in the results^52,55^. A time series y (t) is modeled as:

5 $$y(t)=S(t;{\Theta }_{s})+T(t;{\Theta }_{t})+\varepsilon (t),\,\varepsilon (t)\sim {{\rm{N}}}(0,{\sigma }^{2})$$

y(t) is the observed value of the time series at time. S(t;Θs) is the seasonal component with parameters Θ_s_. T(t; Θ_t_) is the trend component with parameters Θ_t_ (piecewise linear with changepoints τ_j_). ε(t) is a random noise term (Gaussian white noise). σ^2^ is the variance of the noise. The seasonal signal in each segment is defined as:

6 $$S(t)=\sum _{l=1}^{{L}_{k}}[{a}_{k,l}sin(\frac{2\pi lt}{P})+{b}_{k,l}cos(\frac{2\pi lt}{P})],t\in [{\xi }_{k},{\xi }_{k+1}]$$

L_k_ is the number of harmonics in a segment. a_k,l_ and b_k,l_ are the Fourier coefficients for the sine and cosine terms. P is a known seasonal period (e.g., 12 months or 365 days) and ξ_k_ is the seasonal changepoint at the beginning of segment k. The trend is modeled as a piecewise linear function:

7 $$T(t)={a}_{j}+{b}_{j}t,\,t\in [{\tau }_{j},{\tau }_{j+1}]$$

a_j_ and bj are the intercept and slope of segment j, respectively. τ_j_ is the trend changepoint at the beginning of segment j. The model can be rewritten in matrix form:

8 $$y={X}_{M}{\beta }_{M}+\varepsilon ,\varepsilon \sim N(0,{\sigma }^{2}I)$$

X_M_ is the design matrix determined by the model structure. β_M_ is the coefficient vector (Fourier and trend parameters). I refer to the identity matrix. In the Bayesian inference framework, the posterior distribution is estimated as follows:

9 $$p({\beta }_{M},{\sigma }^{2},M| y)\propto p(y| {\beta }_{M},{\sigma }^{2},M)\cdot p({\beta }_{M},{\sigma }^{2},M)$$

p(y∣⋅) is the conditional likelihood function. p(⋅) is the prior distribution over model parameters and structure. M is the model configuration including the number and location of changepoints and harmonics. Posterior sampling is done using a hybrid MCMC algorithm combining Gibbs sampling and Reversible Jump MCMC (RJ-MCMC). From the posterior samples, we estimate :1) the most probable trend and seasonal components, 2) the probabilities and locations of changepoints and 3) the 95% credible intervals for parameters and decompositions. For details on MCMC settings and other implementation information, refer to the supplementary document.

In BEAST, each pixel is analyzed independently as a separate time series. For each series, the model allows up to five changepoints, producing up to five posterior probabilities, five years of occurrence, and the corresponding segment parameters (e.g., slopes and intercepts). The algorithm directly computes the posterior inclusion probability for each potential changepoint, meaning that each year (or time step) receives an explicit posterior probability of being an abrupt change. In this framework, no fixed probability threshold is required, because all changepoints are ranked and reported according to their posterior probability. The changepoint with the highest posterior probability (typically > 0.90–0.95 in our analysis) represents the most likely abrupt change within that pixel. This value inherently conveys the confidence level of detection. By comparing the corresponding trend slope at that changepoint, we can determine whether the change was positive or negative and identify its exact year. Accordingly, instead of applying a global cutoff, we compared two complementary maps: Slope of abrupt change, showing the magnitude and direction of the detected shift, and Probability of abrupt change, showing the corresponding posterior probability. Together, these two maps provide a complete spatial representation of where, when, and with what intensity abrupt changes occurred.

#### Modified Mann-Kendall (MMK) and Sen’s Slope

The Mann–Kendall test is a widely used nonparametric method for detecting monotonic trends in environmental and hydrological time series. A key assumption of the test is temporal independence. To ensure accurate trend detection in the presence of serial correlation, we applied the MMK test, which adjusts the variance of the test statistic to account for autocorrelation. Prior to testing, we computed the autocorrelation function for each soil-moisture and groundwater-storage series across multiple lags to identify statistically significant dependencies; a lag was deemed significant if its autocorrelation exceeded the two-sided 95% confidence bounds. The maximum significant lag was then used either to adjust the MMK variance or, where appropriate, to remove first-order (and higher-order, if present) autocorrelation through pre-whitening. This procedure minimized bias from serial dependence. Monthly hydrological data often exhibit pronounced seasonal cycles that can obscure or distort long-term trends. To isolate low-frequency variability and eliminate periodic seasonal effects, the monthly data (expressed as percentages) were first de-seasonalized by subtracting the climatological monthly means; trend analysis was then conducted on the resulting anomaly series. Performing this step on percentage-based data does not introduce unit inconsistency, as the anomalies remain in percent units. Sen’s slope was used to quantify trend magnitude and is reported as the median linear rate of change, expressed here as percent per unit time. Given the monthly resolution, the initial Sen’s slope was calculated as percent per month and then converted to percent per year to aid interpretation. Accordingly, throughout the Results section, Sen’s slopes are reported as “percent change per year,” indicating both magnitude and direction. Complete mathematical formulations for the MMK test and Sen’s slope are provided in the Supplementary document.

### Amplified drought

Here, we introduce an operational framework defining a specific form of anthropogenic drought, which we term Amplified Drought. Amplified Drought refers to situations in which drought categories initiated by precipitation deficits relative to long-term conditions evolve into progressively intensified impacts across subsequent drought classifications (e.g., hydrological, agricultural, and economic), varying across locations and underlying drivers. In other words, a similar meteorological drought signal, arising from reduced precipitation over a broad regional extent, may produce divergent and disproportionately amplified responses in hydrological drought indicators within specific countries or regions. Regardless of the underlying mechanisms driving such amplification, quantifying these differential responses is critically important and plays a fundamental role in water resources management. In this context, the present study proposes a time-series–based methodological framework grounded in spatial comparisons at both regional and national scales. Specifically, the temporal evolution of meteorological and hydrological drought indices was analyzed at the country level using multiple independent databases across NA a region characterized by substantial data limitations. Detailed methodological descriptions of the spatiotemporal processing and multi-source dataset analyses conducted at different spatial scales are provided below.

#### Spatiotemporal processing of multi-source datasets

Initially, the BEAST algorithm was applied to all ERA5 datasets, without altering their native spatial or temporal resolutions, to analyze pixel-wise time series of three variables (precipitation, temperature, and evapotranspiration) across NA for the period 1981–2024. Each pixel’s time series was individually evaluated to identify climatic breakpoints based on both the slope and the probability of abrupt changes. Subsequently, monthly GPM precipitation data (1998–2024) were spatially averaged at the national scale for each of the five NA countries. The resulting time series were compared to assess inter-country differences in breakpoints and long-term trends. As in the ERA5 analysis, no artificial resampling or temporal adjustment was performed, except for spatial averaging of the gridded monthly data within each national boundary. Complementary analyses were performed using in-situ observations from 131 active meteorological stations (see Supplementary Files). Among these, 35 stations meeting four rigorous criteria, (1) the longest continuous observation records, (2) minimal data gaps, if missing data did not exceed 30% of the total record length over the period 1994–2024, or if there were no continuous gaps longer than five consecutive years^56^. (3) stable instrumentation and consistent measurement protocols, and (4) uninterrupted reporting up to the present, were classified as best-quality stations. The SPI was computed for all selected stations over 1984–2024, and each point-based series was evaluated independently (Supplementary Files). To further characterize regional-scale drought evolution, ERA5-Drought datasets were employed to derive national-scale monthly SPI-12 and SPI-48 indices for each country over 1954–2024, followed by detailed time-series analyses. In parallel, the SPEIbase dataset was used to compute regional mean SPEI values across Algeria, Tunisia, and Libya, treated as a single area zone, over four accumulation periods (1954–2024) to examine large-scale temporal patterns of drought intensity and persistence. For hydrological drought assessment, satellite gravimetry products were incorporated in a comparative framework to detect breakpoints and long-term variations across NA countries. TWS anomaly variations were computed at a seasonal scale for each pixel and analyzed using the BEAST algorithm for 2002–2024, preserving the native spatial resolution. Results were obtained separately for the CSR and JPL GRACE products. Finally, temporal trends in three key hydrological components, SFSM, RZSM, and GWS, were analyzed using the GRACE-DA-DM dataset for each country over 2003–2024, with particular focus on GWS depletion. In this regard, no datasets in this study were subjected to cross-product spatial or temporal fusion; standard re-gridding procedures were applied only when strictly necessary. For temporal analyses, data were aggregated to monthly, seasonal, or annual scales according to the comparison objectives. For national-scale spatial assessments, all variables were resampled to the finest common grid (0.1°) using the nearest-neighbor method, and spatial means were computed within national boundaries. A uniform boundary shapefile was used for all averaging procedures, with fractional weighting of boundary pixels according to their overlap with national polygons. Consequently, all spatial and temporal analyses were conducted in a methodologically consistent and internally coherent manner.

## Supplementary information

Transparent Peer Review file

Supplementary Information

## Data Availability

The Supplementary Files generated and analyzed during this study have been deposited in the Figshare digital repository and are publicly available at: https://figshare.com/s/57248f9d919bf1f46eab. The source data for Figs. 1–8 are provided in the accompanying source data file entitled “Figures Data”.

## Code availability

The Bayesian Estimator of Abrupt change, Seasonal change, and Trend (BEAST) algorithm is publicly available at [https://github.com/beast-dev/beast-mcmc]^52,53^. The underlying code for this study is not publicly available but may be made available to qualified researchers on request from the corresponding author.
