# Supplementary material for: Spatiotemporal variations in hydrometeorological drought across North Africa and indications of anthropogenic amplification
Source: Commun Earth Environ. 2026 Jul 13;7(1):871. doi: 10.1038/s43247-026-03807-2 (PMC13736680; doi:10.1038/s43247-026-03807-2)
Supplement: Supplementary file 1 [file 43247_2026_3807_MOESM1_ESM.pdf]

nature portfolio

Peer Review File

Spatiotemporal variations in hydrometeorological drought across North Africa and indications of anthropogenic amplification

Corresponding Author: Dr Morteza Rahimpour

This file contains all reviewer reports in order by version, followed by all author rebuttals in order by version.

Version 0:

Reviewer comments:

Reviewer #1

(Remarks to the Author)
Dear Editor of Communications Earth & Environment,
The manuscript treats an important topic and uses many powerful datasets (ERA5-Land, IMERG, GRACE, SPEI, etc.), but major methodological, data-consistency, statistical, and presentation problems undermine the conclusions. Substantial revision is needed before the work can be considered reliable.
Major scientific weaknesses:
• Conflicting statements about ERA5 / ERA5-Land data period and baseline. The text says ERA5-Land provides "high-resolution land surface reanalysis data from 1980 to 2024" (lines 110–112), yet Table 1 (and other places) lists ERA5-Land as 1950-present (lines 114–116). Different figures/analyses reference different baseline periods (e.g., Fig.5 uses 1954–2024; elsewhere, 1980–2024 is used). These inconsistencies cast doubt on which baseline was used.
• The manuscript reports 280 GHCN stations, 131 active to 2024 and 76 with >30 years (lines 98–104), but elsewhere it states the analysis used ERA5/remote products due to sparse observational records (lines 236–238, 236–240), and many figures use gridded/remote outputs. It's unclear whether station observations were used for validation, for independent trend analysis, or only for illustrative plots. Please, state which analyses used in-situ GHCN station observations (provide station IDs/time coverage), which used gridded products (ERA5, IMERG), and whether station time series were interpolated or compared directly. Provide station-by-station validation statistics, if any.
• Multiple datasets with different spatial resolutions and temporal coverage are used (GPM IMERG, ERA5-Land, GRACE JPL/CSR, GRACE-DA-DM, SPEIbase), but the manuscript lacks detail on regrading, temporal aggregation (monthly vs seasonal), masking, and how percentiles/indices were computed on different grids. Without this, comparisons and trend tests may be invalid (dataset list and table lines 94–127; GRACE/percentiles description lines 116–124).
• The manuscript describes BEAST and its equations (lines 148–174), but important implementation details are missing. Specifically, the authors do not report the MCMC settings (e.g., number of iterations, burn-in), the priors used, or any convergence diagnostics. It also remains unclear how the changepoint probabilities were calculated and how results were aggregated across pixels. Later, when the results reference pixel-by-pixel changepoints (lines 200–214), the problem becomes more evident: performing changepoint detection independently for many pixels creates a multiple-testing issue, yet the paper does not mention any correction (e.g., false discovery rate, family-wise error control) or clarify how the changepoint probability maps were thresholded to identify significant changes.
• The MMK is stated, and equations are included (lines 175–191), but the manuscript does not state how many lags were considered significant, whether seasonality was removed before application (monthly series have strong seasonality), or whether methods were used. The Sen's slope is reported in per cent (Table 2) without units or base, per cent of what? This prevents interpretation.
• The manuscript attributes dramatic Tunisia groundwater decline after 2010 to anthropogenic pumping (lines 346–351, 374–377) based largely on GRACE monthly TWSA and GRACE-DA percentiles. GRACE is coarse and integrative (TWS includes soil moisture, surface water, snow); the authors note product differences (CSR vs JPL) and advise against averaging (lines 312–316) but then draw strong attributional conclusions. They provide no direct groundwater abstraction/usage data, well measurements, or socio-economic evidence.
• The manuscript states that pixel-level analyses show localised significant changes but "these changes are not spatially extensive enough to be statistically significant at larger scales, such as the national level" (lines 234–236, 280–284), yet later asserts "Since 1998 the region has experienced sustained drying" (lines 280–284, 382–388). The jump from localised

pixel changes to a statement about the entire region is not justified.
Specific Comments
• Table 2 shows extremely large Z values (e.g., 35.0) and Sen's slope as a percentage (e.g., 5.6%). The meaning of these percentages and how they were calculated (per cent of what baseline?) is not defined. Also, the p-value is shown as "0.0000" without precision.
• The text says no clear trends at the country level and BEAST/MK confirm no significant trends in some series (lines 234–240), yet claims an unprecedented drought beginning in 2019 that "intensified each year since" (lines 249–267) and asserts continuous drying since 1998 (lines 280–284, 382–388). Which is it? Localised vs regional? The statements conflict.
• The groundwater decline in Tunisia is linked to human extraction (lines 346–351, 374–381) despite the manuscript admitting a lack of access to observational data and heavy reliance on remote sensing (lines 54–56, 94–101). The evidence is suggestive but not sufficient for attribution.
• In lines 98–105, the manuscript notes that 280 GHCN stations were available and that 76 with more than 30 years of data were selected, but the criteria for "high quality" versus "low quality" remain vague. It is not clear whether the GHCN station series were actually used for trend tests or only shown in descriptive plots. The authors should clearly specify the selection process (e.g., minimum record length, completeness thresholds, quality flags), provide the list of station IDs used in analyses, and state explicitly which stations were included in statistical tests versus which were excluded. If in-situ data were not used for validation or formal testing, this needs to be stated plainly so as not to give the impression of independent observational support where none was applied.
• The GRACE-DA-DM product percentiles are said to be based on historical records from 1948–2014 (line 121). Yet analyses extend to 2024 (e.g., Fig.7 lines 331–341). Explain how percentiles and baselines were computed beyond 2014 and how you addressed differences between CSR and JPL signals. Please provide a sensitivity analysis using both products.
• Typos and inconsistent naming: e.g., "metrological" vs "meteorological" repeated (line 200 et seq.), inconsistent SSP notation (SSP2_4.5 vs SSP2-4.5) — tidy throughout. (Multiple places: lines 200–210, lines 270–285).
• Overstatements in Abstract/Conclusion: Abstract claims precise magnitudes (SPEI drop from −0.26 to −2.27) and "average decline exceeding 6% across all indices" (lines 27–36) without uncertainty or a method for computing "average decline in %". Provide CI and method.

Reviewer #2

(Remarks to the Author)
This study looks at how droughts have changed over time in North Africa, especially in Tunisia, Algeria, and Morocco. It shows that since 1998, the region has been getting drier, with less rainfall and higher temperatures. A particularly severe drought started around 2021 and is still ongoing in 2024 which is the worst in the past 60 years. Authors attributed these droughts to climate change and added that human dependence on groundwater, are making things worse, especially in Tunisia. The study mentions that looking at just one type of drought (like rainfall) isn't enough. A multi-dimensional approach considering both weather and water use is needed to understand and manage droughts better.
I have the following comments on the manuscript for authors consideration:
1. Section 2.4 can be avoided by simply citing the testes/parameters/indicators used, and keeping only the explaining how these indicators (Bayesian time-series decomposition using BEAST, The Mann–Kendall test, and the Sen's slope) are used for the study.
2. Line 206, 208: phrase 'this figure' used can cause confusion please try and be specific and indicate the figure number to avoid ambiguity.
3. Line 202: 'section e' please add the full figure number with the panel. Please be consistent with the terminology, 'section' here was earlier mentioned as 'parts' in line 205.
4. Line 202: 'other maps', please be specific about which maps you are comparing.
5. Section 3.1.1 while mentioning the comparison of maps, please mention how the maps were compared and what were the indicators used for the quantification of differences/similarities between the maps.
6. Line 230-231: 'These findings are consistent with results from other studies using different data sources', please a small section or few lines be added here explaining the similarities and differences with other studies cited.
7. Figure 3 and section 3.1.1: please explain how the time series are produced using the gridded data for better clarity.
8. Figure 4 and section 3.1.2: it would be great if the locations of the stations mentioned in the figure 4 can be added to a map for context. It will me much of meaningful and add a great deal of value to the comparisons made.
9. Section 3.1.2 in figure 4 the graphs shown were of individual stations, comparing those with an area averaged series of three countries (figure 5) doesn't seems sensible. I think it would be interesting to see the comparison with the area averages series over the individual countries.
10. Lines 295-316: majority of the section 3.2 should be the part of discussion section. The manuscript needs a bit of rearranging in terms of results and discussion sections.
11. The study mentioned that the results and finding the consistent with previous studies, please add a section in the discussion to explain the consistency, differences, nuances and novelty of this study in context with the existing literature.

Reviewer #3

(Remarks to the Author)
This manuscript presents an interesting contribution in the study of drought in the Mediterranean region and I appreciate the interest to monitor not only meteorological drought but also hydrological. Due to the lack of meteorological data recorded at the ground the authors combined information belonging from different sources. However, several aspects of the manuscript would benefit from clarification, reorganization, and methodological improvement:

Mandatory revisions include:
• Providing detailed information about the study area.
• Revising figure color scales and labels for clarity.
• Reorganising the Results, Discussion, and Conclusion sections.
• Improving the description of the datasets, with particular attention to the methodology adopted for each and common study period.
• Recalculating SPI and SPEI indices using the ERA5-Land product to avoid comparing indices derived from different databases with varying temporal and spatial resolutions, which is methodologically incorrect.

Study Area:
Suggest that the authors provide more information on the study area to better address the drought issue, including details on:
Geomorphology
Climatic characteristics
Vegetation distribution (cultivation and natural vegetation)
Population distribution and water consumption

Material and Methods.
The authors have adopted multiple datasets encompassing ground-based observations, reanalysis products, and remote sensing sources. It is recommended that the authors improve the description and methodological clarity regarding each dataset, providing sufficient detail to ensure the methodology flow.
For example, at line 100, the manuscript states that precipitation time series were employed to compute the SPI index. However, the drought time scale applied (e.g., 12-, 24-, or 36-month) is not specified. Moreover, the common period of study of the ground-based precipitation series remains unclear.
Going through the paper in the result section, the authors also analyse the SPEI index, which is derived from the SPEI Global Drought Monitor at a spatial resolution of 0.5°. Comparing indices computed from different data sources, at different spatial resolution and time period, represents a major methodological inconsistency that may compromise the robustness of the results. It is therefore strongly recommended that both SPI and SPEI indices be recalculated using the ERA5-Land database to ensure methodological consistency and reliability of the comparative analysis.
Table 1 and Line 207:
The authors should clarify what is meant by “temperature.” Does this refer to mean, maximum, or minimum temperature? Providing this specification is essential for the accurate interpretation of the results.

Figure 2 and Line 210:
The choice of colour scheme must be checked. It is unclear why significant changes are represented in red and non-significant changes in green. This convention may lead to misinterpretation, as red is typically associated with not significant trend.

Figure 2 (Slope colour scale):
The slope colour scale appears inverted for temperature and evapotranspiration, which complicates visual interpretation. The authors should harmonise the colour scale across all variables.

Line 224:
A clear negative trend in evapotranspiration (ET) is observed in southern Egypt. The authors, however, state that ET exhibits a positive trend. Please check this incongruity.

Line 225:
In this case precipitation recorded main negative changes for the southern Egypt region.

Line 226:
This sentence repeats information already provided a few lines earlier and should therefore be removed to avoid redundancy.

Line 227:
The content of this line is more appropriate for the Discussion section, as it involves interpretation rather than direct presentation of results. The authors are encouraged to relocate it accordingly.

Line 242:
The criteria used to select the eight representative stations are not clearly described. The authors should specify the selection base on geographic position, data completeness, climatic representativeness

Line 247: It’s discussion

Figure 4:
It is recommended to include, within the figure, the name of each station and the corresponding country. This addition would significantly improve the clarity and contextual understanding of the results.

Line 255 Why the authors decided to average SPEI over the entire NA and create a single SPEI series for the entire study area? It is clear that there are different drought behaviours for each country. Due to the complexity of drought events it is important investigate on the temporal variability, but the spatial is important too. As the authors did for precipitation,

temperature and ET form ERA5Land products

Line 273:
It is unclear how this conclusion was derived from the temporal series analysis. Did the authors achieved this result through comparison with the eight reference series? Further clarification of the methodology behind this statement is required. Therefore, I strongly emphasize the importance of analysing drought dynamics also at the spatial scale to capture the regional variability of the phenomenon.

Line 302:
The content presented here describes methodological procedures and should be relocated to the Materials and Methods section for better structural coherence.

Line 314:
This a discussion

Figure 6:
Please confirm figure refers to the TWSA series. If so, this should be explicitly stated in the figure legend to avoid ambiguity.

Line 343
It's discussion

Discussion
All discussion-related statements currently included in the Results section should be relocated to the Discussion section to improve the structural clarity of the manuscript.
Moreover, the authors are encouraged to develop this section further by exploring in greater depth the relationships between meteorological drought and the climatological variables. Incorporating statistical analyses, such as Pearson's correlation test between drought indices and climatological variables.

The results related to Groundwater Storage (GWS) are particularly important, and the authors are encouraged to place greater emphasis on this aspect, as it represents a valuable contribution to the understanding of drought processes in the study area. What about bibliography on human water consumption?

An important strength of the manuscript lies in the extensive effort devoted to the collection and processing of multiple datasets, each providing distinct and complementary types of information. This integrative approach is particularly valuable given the significant data gaps that characterise the study area. The authors are encouraged to emphasis this aspect more explicitly in the manuscript, as it represents a novel and commendable contribution to regional drought assessment and climatological research.

Conclusion:
The Conclusion section should be revised to reflect the new insights and interpretations arising from the expanded Discussion section.

Version 1:

Reviewer comments:

Reviewer #1

(Remarks to the Author)
I appreciate the authors' efforts to revise the manuscript and address many of the concerns raised in the first round. The revised version shows improvement in structure and clarity; however, several key issues remain insufficiently resolved, particularly regarding data consistency, methodological transparency, and the strength of attribution claims.

Remaining Major Concerns

Data Consistency and Baseline Definition
There are still inconsistencies regarding the temporal coverage and baseline periods used for ERA5/ERA5-Land and related datasets. Different sections and figures appear to rely on different reference periods without clear justification. This lack of consistency makes it difficult to fully assess the robustness of trends, percentiles, and drought classifications.

Use of Observational (GHCN) Data
While GHCN stations are mentioned, their exact role remains unclear. It is still not explicitly stated whether station data are used for validation, independent trend analysis, or illustrative purposes only. Given the importance of observational support, this point requires clearer explanation.

Changepoint Detection and Statistical Interpretation
The BEAST changepoint analysis is better described, but key implementation details (e.g. priors, convergence checks, interpretation of spatially extensive changepoints) remain limited. In addition, the manuscript does not sufficiently

acknowledge the implications of applying pixel-wise changepoint detection over large spatial domains.

Attribution to Anthropogenic Influences
The revised text still makes relatively strong claims linking drought amplification to anthropogenic drivers, including groundwater abstraction. While the patterns presented are suggestive, direct attribution remains unsupported by independent socio-hydrological or land-use data. Given that anthropogenic influence is highlighted in the title, these conclusions should be more cautiously framed or further substantiated.

Regional vs Local Interpretation
Some conclusions extrapolate from localised signals to regional-scale drying without sufficient justification. Statements regarding widespread or sustained regional drying should be more carefully aligned with the spatial extent of statistically robust results.

Minor Issues

Some statistical outputs (e.g. p-values reported as 0.0000, very large Z-values) remain difficult to interpret and should be presented more carefully.

Minor language and consistency issues persist and would benefit from a final editorial pass.

Overall,
The manuscript addresses an important topic and has improved since the first review. However, key methodological clarifications and a more cautious interpretation, especially regarding anthropogenic attribution, are still required. Addressing these points would substantially strengthen the credibility and impact of the study.

Reviewer #2

(Remarks to the Author)
Thank You for all your comments, the modified manuscript and comments seems satisfactory.

Reviewer #3

(Remarks to the Author)
The authors have incorporated the vast majority of the suggestions made by all reviewers in a satisfactory way, which has significantly strengthened the paper. The structure is clearer, the methodological explanations are more transparent. Overall, the manuscript reads much more cohesively and makes a stronger contribution to the field.

I have only minor revisions to suggest:

Figure1 Please add the toponyms mentioned in the manuscript (e.g., Nile, Medjerda, Moulouya) are not clearly located on the map, which may confuse readers unfamiliar with the area.

“2.5. Spatiotemporal processing of multi-source datasets the sentence “minimal data gaps” which percentage of missing data did the authors considered? Add reference

Version 2:

Reviewer comments:

Reviewer #1

(Remarks to the Author)
Thank you for revising the manuscript and addressing several of the earlier comments; however, there remain a number of important methodological, statistical, and interpretative issues that need to be resolved before the conclusions can be considered fully supported.

1-Inconsistent analysis periods and baselines. Key datasets (ERA5-Land, SPI, SPEI) are analysed over different and sometimes inconsistently stated periods (e.g., 1980–2024 vs 1981–2024), without a clearly defined, uniformly applied reference baseline, complicating trend and breakpoint interpretation.
2-Although many GHCN stations are available, it remains unclear which results are directly supported by station-based analyses versus gridded products, and whether station data provide validation or independent evidence for the main conclusions.
3-No formal comparison (e.g., bias, correlation, trend or changepoint agreement) is provided between station observations and reanalysis, limiting confidence in cross-dataset consistency.
4-The manuscript lacks clear operational descriptions of regridding, temporal aggregation, masking, and index computation across datasets with different resolutions and coverage, weakening reproducibility.
5- Essential implementation details (priors, MCMC settings, convergence checks, and treatment of multiple testing in pixel-wise analyses) are missing, making the robustness of detected changepoints difficult to assess.

6- Handling of serial correlation and seasonality is not fully specified, and Sen's slopes are reported in percent without clearly defining the reference quantity, hindering physical interpretation.
7-Attribution of Tunisia's groundwater decline to human pumping relies mainly on GRACE-based indicators without direct abstraction, well, land-use, or socio-economic data, so causality is inferred rather than demonstrated.
8-The manuscript alternates between localised pixel-scale changes and claims of sustained regional drying without a clear methodological bridge linking the two scales.
9-The term "amplified drought" is central to interpretation but lacks a formal operational definition or quantitative criteria.
10-Abstract and conclusions include strong quantitative and attributional statements without a clear explanation of how aggregate metrics were computed or how uncertainty was assessed.
11- Placing the legend inside the figure affects the aesthetic balance of the map; moving it outside the mapped area would enhance readability and visual quality
12-Minor but systematic presentation issues. Typos and inconsistent terminology reduce clarity, though they do not affect the core results.
e.g., IMMERG vs IMERG in the Fig. 3 caption; ERA5 vs ERA5-Land; change point vs changepoint/breakpoint)
Hargreaves metho" (typo) in the SPEI methods description (L170)

Reviewer #2

(Remarks to the Author)
I have reviewed the revised manuscript and the authors responses. I appreciate the efforts made to address the concerns raised during the earlier review. The revisions have improved the clarity and quality of the manuscript, and the authors have adequately responded to the comments and suggestions.

n my opinion, the concerns raised in the previous review have been satisfactorily addressed. Therefore, I am happy to recommend the manuscript for publication in its current form.

Open Access This Peer Review File is licensed under a Creative Commons Attribution 4.0 International License, which permits use, sharing, adaptation, distribution and reproduction in any medium or format, as long as you give appropriate credit to the original author(s) and the source, provide a link to the Creative Commons license, and indicate if changes were made.
In cases where reviewers are anonymous, credit should be given to 'Anonymous Referee' and the source.
The images or other third party material in this Peer Review File are included in the article's Creative Commons license, unless indicated otherwise in a credit line to the material. If material is not included in the article's Creative Commons license and your intended use is not permitted by statutory regulation or exceeds the permitted use, you will need to obtain permission directly from the copyright holder.
To view a copy of this license, visit https://creativecommons.org/licenses/by/4.0/

Dear Reviewers,

We would like to express our deepest gratitude for your thorough review and insightful comments on our manuscript entitled "*Spatiotemporal hydrometeorological drought variations in North Africa: indications of anthropogenic contributions to amplify drought*". We sincerely appreciate the considerable time and effort you devoted to providing such detailed and constructive feedback. Your comments and suggestions have been invaluable in improving the quality, clarity, and scientific rigor of our work.

Below, we provide a detailed, point-by-point **response** (in black) to each of the **reviewers**' comments (**in blue**). For your convenience, each response is numbered as a heading and organized according to the corresponding comment in the "**Reviewers' Responses to Questions**." All modifications are clearly indicated with highlighting in yellow for ease of review.

Following your valuable feedback, we thoroughly revised and expanded the manuscript. Major modifications include substantial revisions to the Abstract, Materials and Methods, Results, and Conclusion sections. In particular, new analyses were conducted incorporating the SPI-12 and SPI-48 indices using ERA5 data. A new figure and its associated discussion have been added to the Results section.

To further enhance transparency and reproducibility, we have also deposited an extended set of supplementary datasets and materials in an open repository (DOI:10.6084/m9.figshare.30582071). Consequently, the Materials and Methods, Results and Discussion sections underwent the most substantial restructuring. Within the "Materials and Methods", the subsections "2.1. Data: Observation, Global Datasets and Remote Sensing" and "2.2. Study Area" were revised to integrate all reviewer suggestions. Several equations were moved to the supplementary information for improved readability, and new explanatory text was added. Additionally, a new subsection titled "2.5. Spatiotemporal processing of multi-source datasets" was introduced to describe the analytical framework in greater detail. Finally, the Results and Discussion sections have been reorganized into a single integrated narrative to improve logical flow and clarity.

We believe these comprehensive revisions have significantly strengthened the manuscript and aligned it more closely with the reviewers' valuable recommendations.

With our sincere appreciation,

The Authors

Reviewers’ Responses to Questions:

1) Reviewer #1:

The manuscript treats an important topic and uses many powerful datasets (ERA5-Land, IMERG, GRACE, SPEI, etc.), but major methodological, data-consistency, statistical, and presentation problems undermine the conclusions. Substantial revision is needed before the work can be considered reliable. Major scientific weaknesses:

1.1- Conflicting statements about ERA5 / ERA5-Land data period and baseline. The text says ERA5-Land provides “high-resolution land surface reanalysis data from 1980 to 2024” (lines 110–112), yet Table 1 (and other places) lists ERA5-Land as 1950-present (lines 114–116). Different figures/analyses reference different baseline periods (e.g., Fig.5 uses 1954–2024; elsewhere, 1980–2024 is used). These inconsistencies cast doubt on which baseline was used.

Response: We thank the reviewer for drawing our attention to this issue and to the confusion it causes. The reported data periods are actually correct, but require additional clarification and text revisions. As discussed throughout the manuscript, the study area suffers from a severe lack of observational data, and in some cases, inconsistencies have been reported in the available ground observations. Therefore, our important objective was to make full use of all available datasets to achieve a comprehensive assessment. However, each dataset covers a specific temporal range period. For example, satellite products are only available from 2002 onward, whereas reanalysis data extend back to the 1940s. We were faced with two options: 1) either to adopt a short common baseline period across all data sources, which would have caused the loss of a substantial portion of the reanalysis record; or 2) to analyze each dataset separately. Since this study focuses not on the exact estimating value of variables but on time-series behavior, extreme variations, and changepoint analysis in nonstationary series, analyzing different databases with varying temporal lengths allowed us not only to identify critical periods but also to compare multiple data sources and avoid misinterpretation due to source-specific errors or nonstationary in any single dataset.

Because reanalysis products for the early decades exhibit significantly higher uncertainty, and to preserve statistical representativeness, adopting a very short reference period was not appropriate. Therefore, for each analytical stage, we selected the time period that provided the best balance between data length and reliability while ensuring the statistical validity of the applied methods. Consequently, for each product or database, the “Period Used” may differ from the “Period Available.” Specifically, when using ERA5-Land for changepoint analysis, the applied temporal coverage in this study was 1981–2024 (Figure 2), consistent with the availability of ERA5-Land and the WMO 30-year climatological baseline (1991–2020), although the dataset itself spans 1950–present (lines 114–116). For the long-term drought-trend and extreme-event analysis across the entire region using the SPEIbase dataset, we employed the 1954–2024 period to preserve the robustness of multi-decadal variability assessment, while the dataset is available from 1950–present.

The Data and Methodology sections have been revised and corrected accordingly. Specifically, to address this comment and improve clarity for readers, a new column titled "Period Used" has been added alongside "Period Available" in Table 1, and this rationale has been explicitly described in Section 2.2, Data: Observation, Global Datasets, and Remote Sensing. Again, the authors thank the reviewer for having pointed out these details and the confusion they may cause.

Edited manuscript (line 88-96):

"The study area is characterized by a pronounced scarcity of ground-based observational data. To address this limitation and ensure a comprehensive evaluation, a broad suite of global databases encompassing the maximum available temporal coverage was employed. As a result, uniform baseline periods were not imposed across all datasets, since doing so would have excluded valuable temporal information. Given the objectives of this research, defining a fixed reference period was unnecessary, as the primary aim was to detect major fluctuations and assess the non-stationarity of spatiotemporal hydro-meteorological drought variations through a comparative framework, rather than to quantify absolute variable magnitudes. Accordingly, multiple datasets with varying temporal spans were integrated into the analysis. As summarized in Table 1, the "period used" in this study may differ from the "period available" originally provided by each data source."

1.2- The manuscript reports 280 GHCN stations, 131 active to 2024 and 76 with >30 years (lines 98–104), but elsewhere it states the analysis used ERA5/remote products due to sparse observational records (lines 236–238, 236–240), and many figures use gridded/remote outputs. It's unclear whether station observations were used for validation, for independent trend analysis, or only for illustrative plots. Please, state which analyses used in-situ GHCN station observations (provide station IDs/time coverage), which used gridded products (ERA5, IMERG), and whether station time series were interpolated or compared directly. Provide station-by-station validation statistics, if any.

Response: We appreciate the reviewer's thorough review and careful attention to this point. As mentioned earlier, our aim was to maximize the scientific use of all available ground observations. Accordingly, the GHCN station data were employed for both validating the SPI-12 drought index and for conducting independent trend analyses of the in-situ time-series records. However, since GHCN observations are directly assimilated into the ERA5-Land reanalysis, a point-to-point comparison between ERA5-Land and GHCN cannot be considered an independent validation. Although this comparison was initially performed, the resulting point-based accuracy was already very high; therefore, we decided not to include it as a formal validation result. Consequently, no spatial interpolation was applied, and all GHCN station observations were analyzed and used individually.

Because the main objective of this study was not to estimate absolute magnitudes of variables but to analyze asymmetric temporal variations, none of the gridded products (ERA5, IMERG) were

separately validated against in-situ data; instead, they were analyzed as independent sources. For both SPI-12 validation and independent trend analyses, all comparisons were conducted on a point-to-point or point-to-grid basis, and their results are fully provided. Given the limited spatial density and uneven distribution of ground stations, no spatial interpolation of the station time series was performed.

To avoid any misunderstanding, the Data and Methodology sections have been thoroughly revised for clarity. In accordance with the reviewer's recommendation ("Provide station-by-station validation statistics, if any"), we have now included complete station-based time-series analyses (trend, seasonal, and residual components) for all 131 active stations, as well as the SPI-12 series for the 30 long-term stations, in the Supplementary Information. These additions improve transparency and reproducibility and provide a valuable reference for future research in a region where in-situ observations are often sparse or incomplete. Each figure file (.jpg) is named according to its corresponding station ID listed in Supplementary Table S3, which also includes the station identifiers, geographic coordinates, and record lengths.

Edited manuscript (line 104-109):

"Due to the limited spatial coverage and uneven distribution of GHCN stations across North Africa, no spatial interpolation of the station time series was performed. Instead, all analyses were conducted on a point-to-grid basis. The GHCN observations were used both to validate the SPI-based drought indices and to perform independent trend analyses using in situ series. Comprehensive plots showing the trend, seasonal, and residual components for all 131 active stations (by station name), as well as SPI time series for 35 selected stations (red point at Fig.1), are provided in the Supplementary Files."

Links:

– 131 Stations_decomposition-timeseries: [https://figshare.com/s/57248f9d919bf1f46eab]

– 35 Stations_SPI-3-6-9-12-24-48: [https://figshare.com/s/57248f9d919bf1f46eab]

1.3- Multiple datasets with different spatial resolutions and temporal coverage are used (GPM IMERG, ERA5-Land, GRACE JPL/CSR, GRACE-DA-DM, SPEIbase), but the manuscript lacks detail on regrading, temporal aggregation (monthly vs seasonal), masking, and how percentiles/indices were computed on different grids. Without this, comparisons and trend tests may be invalid (dataset list and table lines 94–127; GRACE/percentiles description lines 116–124).

Response: We thank the reviewer for this comment and agree that this information needs to be made clearer. We believe this concern arose from the structure of the previous Data and Methodology sections, which have now been revised for clarity. We hope that the changes brought to these sections provide all this information with more clarity.

Edited manuscript (line 235-265):

“2.5. Spatiotemporal processing of multi-source datasets

Initially, the BEAST algorithm was applied to all ERA5 datasets, without altering their native spatial or temporal resolutions, to analyze pixel-wise time series of three variables (precipitation, temperature, and evapotranspiration) across NA for the period 1981–2024. Each pixel’s time series was individually evaluated to identify climatic breakpoints based on both the slope and the probability of abrupt changes. Subsequently, monthly GPM precipitation data (1998–2024) were spatially averaged at the national scale for each of the five NA countries. The resulting time series were compared to assess inter-country differences in breakpoints and long-term trends. As in the ERA5 analysis, no artificial resampling or temporal adjustment was performed, except for spatial averaging of the gridded monthly data within each national boundary. Complementary analyses were performed using in-situ observations from 131 active meteorological stations (see Supplementary Material). Among these, 35 stations meeting four rigorous criteria, (1) the longest continuous observation records, (2) minimal data gaps, (3) stable instrumentation and consistent measurement protocols, and (4) uninterrupted reporting up to the present, were classified as best-quality stations. The SPI was computed for all selected stations over 1984–2024, and each point-based series was evaluated independently (Supplementary Material). To further characterize regional-scale drought evolution, ERA5-Drought datasets were employed to derive national-scale monthly SPI-12 and SPI-48 indices for each country over 1954–2024, followed by detailed time-series analyses. In parallel, the SPEIbase dataset was used to compute regional mean SPEI values across Algeria, Tunisia, and Libya, treated as a single area zone, over four accumulation periods (1954–2024) to examine large-scale temporal patterns of drought intensity and persistence. For hydrological drought assessment, satellite gravimetry products were incorporated in a comparative framework to detect breakpoints and long-term variations across NA countries. TWS anomaly variations were computed at a seasonal scale for each pixel and analyzed using the BEAST algorithm for 2002–2024, preserving the native spatial resolution. Results were obtained separately for the CSR and JPL GRACE products. Finally, temporal trends in three key hydrological components, SFSM, RZSM, and GWS, were analyzed using the GRACE-DA-DM dataset for each country over 2003–2024, with particular focus on GWS depletion. In this regard, no datasets in this study were subjected to cross-product spatial or temporal fusion; standard regridding procedures were applied only when strictly necessary. For temporal analyses, data were aggregated to monthly, seasonal, or annual scales according to the comparison objectives. For national-scale spatial assessments, all variables were resampled to the finest common grid (0.1°) using the nearest-neighbor method, and spatial means were computed within national boundaries. A uniform boundary shapefile was used for all averaging procedures, with fractional weighting of boundary pixels according to their overlap with national polygons. Consequently, all spatial and temporal analyses were conducted in a methodologically consistent and internally coherent manner.”

1.4- The manuscript describes BEAST and its equations (lines 148–174), but important implementation details are missing. Specifically, the authors do not report the MCMC settings (e.g., number of iterations, burn-in), the priors used, or any convergence diagnostics. It also remains unclear how the changepoint probabilities were calculated and how results were aggregated across pixels. Later, when the results reference pixel-by-pixel changepoints (lines 200–214), the problem becomes more evident: performing changepoint detection independently for many pixels creates a multiple-testing issue, yet the paper does not mention any correction (e.g., false discovery rate, family-wise error control) or clarify how the changepoint probability maps were thresholded to identify significant changes.

Response: We thank the reviewer for this valuable suggestion. All the mentioned details have now been incorporated into the Methodology section, and this portion of the manuscript has been fully revised. Following your recommendation (and those of the other reviewers), we moved less critical content, such as the Modified Mann–Kendall and Sen's slope equations, to the Supplementary Information and expanded the main text discussion of the BEAST model, including its implementation and statistical configuration, to enhance reproducibility.

The revised sections now explicitly report the MCMC settings, priors and hyperparameters, computation of changepoint probabilities, thresholding procedures, multiple-testing considerations, and pixel-level aggregation methods. A concise summary is provided in the manuscript, while full computational details are given in the Supplementary document.

Supplementary document (lines 15–34):

1) MCMC settings and convergence:

Four parallel RJ-MCMC chains were executed for each pixel, each consisting of 60,000 iterations, with the first 20,000 discarded as burn-in and a thinning factor of 10, resulting in 16,000 posterior samples for inference. Random seeds were recorded for reproducibility. Convergence was evaluated through trace plots, the Gelman–Rubin statistic ($\hat{R} \leq 1.05$), and effective sample size (ESS > 500) diagnostics. These settings follow the recommendations of Zhao, et al. [1],Zhao, et al. [2] and Hu, et al. [3].

2) Priors and hyperparameters:

We now include the explicit prior forms: (1) a discrete uniform prior on the number of changepoints (0–5 per layer), (2) conditional uniform priors on changepoint locations, (3) Gaussian priors with broad variances for the trend and seasonal coefficients, and (4) a weak inverse-gamma prior for the noise variance.

3) Computation of changepoint probabilities:

The posterior inclusion probability (PIP) for each time step is defined as the proportion of posterior samples in which that time is identified as a changepoint. This metric is directly derived from the RJ-MCMC sampling process, following Zhao, et al. [2].

Edited manuscript (Lines: 205-216):

4) Thresholding and multiple-testing control and Spatial aggregation:

"In BEAST, each pixel is analyzed independently as a separate time series. For each series, the model allows up to five changepoints, producing up to five posterior probabilities, five years of occurrence, and the corresponding segment parameters (e.g., slopes and intercepts). The algorithm directly computes the posterior inclusion probability for each potential changepoint, meaning that each year (or time step) receives an explicit posterior probability of being an abrupt change. In this framework, no fixed probability threshold is required, because all changepoints are ranked and reported according to their posterior probability. The changepoint with the highest posterior probability (typically > 0.90–0.95 in our analysis) represents the most likely abrupt change within that pixel. This value inherently conveys the confidence level of detection. By comparing the corresponding trend slope at that changepoint, we can determine whether the change was positive or negative and identify its exact year. Accordingly, instead of applying a global cutoff, we compared two complementary maps: Slope of abrupt change, showing the magnitude and direction of the detected shift, and Probability of abrupt change, showing the corresponding posterior probability. Together, these two maps provide a complete spatial representation of where, when, and with what intensity abrupt changes occurred."

1.5- The MMK is stated, and equations are included (lines 175–191), but the manuscript does not state how many lags were considered significant, whether seasonality was removed before application (monthly series have strong seasonality), or whether methods were used. The Sen's slope is reported in per cent (Table 2) without units or base, per cent of what? This prevents interpretation.

Response: We sincerely thank the reviewer for their careful and constructive comments regarding the methodological implementation of the Modified Mann–Kendall (MMK) test and Sen's slope estimator. The Methodology section has been revised accordingly. Specifically, the equations have been relocated to the Supplementary Information to streamline the main text, while additional explanations have been added to clarify the following points:

1) The procedure used to evaluate and correct for time-series autocorrelation prior to applying the MMK test.
2) The removal of seasonality from monthly datasets to avoid bias arising from periodic fluctuations.
3) The criterion used to identify statistically significant lag values.
4) The interpretation of Sen's slope expressed in percentage units, which represents the relative rate of change (%) with respect to the long-term mean value of each variable.
5) These revisions ensure full methodological transparency and address the reviewer's concerns regarding the reproducibility and interpretability of the statistical trend analyses.

Mnuscript (lines 218-234):

“The Mann–Kendall test is a widely used nonparametric method for detecting monotonic trends in environmental and hydrological time series. A key assumption of the test is temporal independence. To ensure accurate trend detection in the presence of serial correlation, we applied the MMK test, which adjusts the variance of the test statistic to account for autocorrelation. Prior to testing, we computed the autocorrelation function for each soil-moisture and groundwater-storage series across multiple lags to identify statistically significant dependencies; a lag was deemed significant if its autocorrelation exceeded the two-sided 95% confidence bounds. The maximum significant lag was then used either to adjust the MMK variance or, where appropriate, to remove first-order (and higher-order, if present) autocorrelation through pre-whitening. This procedure minimized bias from serial dependence. Monthly hydrological data often exhibit pronounced seasonal cycles that can obscure or distort long-term trends. To isolate low-frequency variability and eliminate periodic seasonal effects, the monthly data (expressed as percentages) were first deseasonalized by subtracting the climatological monthly means; trend analysis was then conducted on the resulting anomaly series. Performing this step on percentage-based data does not introduce unit inconsistency, as the anomalies remain in percent units. Sen’s slope was used to quantify trend magnitude and is reported as the median linear rate of change, expressed here as percent per unit time. Given the monthly resolution, the initial Sen’s slope was calculated as percent per month and then converted to percent per year to aid interpretation. Accordingly, throughout the Results section, Sen’s slopes are reported as “percent change per year,” indicating both magnitude and direction. Complete mathematical formulations for the MMK test and Sen’s slope are provided in the Supplementary document.”

1.6- The manuscript attributes dramatic Tunisia groundwater decline after 2010 to anthropogenic pumping (lines 346–351, 374–377) based largely on GRACE monthly TWSA and GRACE-DA percentiles. GRACE is coarse and integrative (TWS includes soil moisture, surface water, snow); the authors note product differences (CSR vs JPL) and advise against averaging (lines 312–316) but then draw strong attributional conclusions. They provide no direct groundwater abstraction/usage data, well measurements, or socio-economic evidence.

Response: We thank the reviewer for raising this important point, which in fact highlights the very motivation for conducting this research.

In this region, we faced two major challenges: first, the lack of accessible and reliable observational data on agricultural water use and groundwater abstraction, or the restriction on their publication; and second, the fact that satellite observations represented the only consistent and publicly available source of hydrological information. The second challenge concerns the coarse spatial resolution and potential bias inherent in satellite products.

As correctly noted, GRACE data provide coarse, mass-integrated measurements that include changes in vegetation, snow and ice, surface water, hydrocarbons, and groundwater. We are fully aware of this limitation and did not attempt to isolate groundwater storage directly from GRACE

data. Rather, the study was designed to evaluate the spatiotemporal behavior of total water storage (TWS) and Groundwater storage percentile (GWS) across all North African countries and to identify abrupt change points through a comparative framework. Because all countries experience similar large-scale mass components (surface water, vegetation, snow, ice), the comparative approach allows us to identify which regions exhibit abnormal or intensified changes under the same observational uncertainty.

In this context, North Africa, particularly the arid and warm environment of Tunisia, shows negligible changes in vegetation, snow, ice, or oil mass, implying that TWS variations primarily reflect groundwater storage changes. However, we did not explicitly quantify groundwater levels, as we are aware of the uncertainty associated with directly deriving groundwater storage from GRACE. Although several previous studies have attempted to estimate groundwater changes from GRACE, our objective here is different: to detect the timing and magnitude of abrupt hydrological transitions, not to calculate absolute groundwater volumes or explain their underlying causes.

To strengthen our findings, we also used GRACE-DA percentile data, which incorporate land surface models and soil-moisture assimilation schemes, providing an independent indicator of the relative magnitude of groundwater storage anomalies. Hence, our focus is on identifying "when and where" major shifts occurred rather than "how and why" they happened. The latter question remains open and will be addressed in a forthcoming study dedicated to groundwater dynamics and governance in Tunisia.

As reflected in the manuscript:

Lines 344–352: "Therefore, droughts driven by climatic changes in the Mediterranean region tend to affect the entirety of Tunisia, whereas only portions of Algeria, Morocco, or Libya may be impacted. However, when comparing Figures 7 and 8 with the plots in the previous sections (Figures 3, 4, and 5), it becomes evident that meteorological drought alone cannot account for the observed hydrological drought in Tunisia. Precipitation levels between 2002 and 2010 were relatively favorable, yet a marked breakpoint and sharp decline in groundwater storage appear around 2010–2011 (Figure 7, purple curve). Thus, it can be concluded that the hydrological drought in Tunisia cannot be solely attributed to meteorological factors. Instead, anthropogenic influences likely play a key role in this dramatic decline. The discussion section examines the underlying causes of this phenomenon in Tunisia in greater detail", state that meteorological drought alone cannot explain Tunisia's post-2010 decline in water storage, as precipitation remained relatively favorable between 2002 and 2010, yet a distinct breakpoint occurred around 2010–2011.

And in lines 373–377: "These challenges are expected to have profound consequences on all aspects of life, particularly for marginalized farmers whose livelihoods are directly tied to groundwater availability 58. Time series analysis also identified a statistically significant breakpoint in 2010, accompanied by a negative trend that cannot be fully explained by meteorological variables. Although drought occurred during this period, our findings indicate that climatic drivers alone do not account for the extent and severity of Tunisia's groundwater

depletion", emphasize that a statistically significant breakpoint accompanied by a strong negative trend cannot be fully explained by meteorological variables.

Similarly, in the conclusion (lines 398–400), we already noted that "However, the important question remains as to whether anthropogenic changes could have contributed to the occurrence of amplified drought. Additionally, the analysis highlights that hydrological drought has affected the entire North African region, primarily driven by the unprecedented meteorological drought from 2021 to 2024"

Thus, at no point does the manuscript draw a direct causal conclusion or direct attributes; rather, it infers a plausible anthropogenic influence based on comparative hydrological evidence. Meteorological drought analyses show that despite favorable precipitation conditions around 2010, Tunisia experienced a pronounced decline in TWS (both CSR and JPL products) and a corresponding drop in GWS (GRACE-DA data). These changes are consistent across independent GRACE solutions and are validated by GRACE-DA trends. When compared with neighboring countries, this anomaly is unique to Tunisia and, without question, most plausibly linked to excessive groundwater extraction.

We also had access to groundwater data in some fractions, including Tunisia, and used it in the assessment. Nevertheless, we did not include direct groundwater abstraction, well-measurement, or socio-economic evidence in the present study for three key reasons:

1. Reliable and comprehensive groundwater-use data are unavailable across North Africa and are classified as confidential even in Tunisia, where access is partial.
2. Although we possess limited observational records from Tunisian stations, we cannot publish or cite them due to verification uncertainty and the potential personal or institutional risks such publication could pose to local collaborators.
3. Most importantly, the current paper does not aim to analyze groundwater withdrawal directly. Its purpose is to detect hydrological regime shifts observed in satellite data, while the question of causation ("why and how") will be explored in future research specifically focused on Tunisian groundwater resources, abstraction rates, and governance mechanisms.

Accordingly, the conclusion has been revised to clearly emphasize this point in edited manuscript (lines 507–508):

", particularly concerning why and how such severe transformations have occurred in Tunisia after 2010."

1.7- The manuscript states that pixel-level analyses show localised significant changes but "these changes are not spatially extensive enough to be statistically significant at larger scales, such as the national level" (lines 234–236, 280–284), yet later asserts "Since 1998 the region has experienced sustained drying" (lines 280–284, 382–388). The jump from localised pixel changes to a statement about the entire region is not justified.

Response: We agree with the reviewer that this point could initially appear contradictory. However, this perception arises mainly from the earlier phrasing, for which we apologize, and which has now been revised for clarity. As noted, the two statements refer to different analytical levels, datasets, time periods, and variables within the manuscript.

In lines 234–240, the text reads: "It is worth noting that these changes are not spatially extensive enough to be statistically significant at larger scales, such as the national level. In other words, when the analysis shifts from pixel-scale to country-scale, extreme changes tend to disappear. Due to the lack of access to observational data, this study utilized multiple data sources, including satellite precipitation data (Fig. 3). Fig. 3 shows the monthly precipitation time series for each of the five countries. As illustrated, no clear trends are visually apparent, and the results of both the BEAST and MK tests confirm the absence of statistically significant trends or change points in any of the time series."

refers to the satellite-based precipitation data (1998–2024) and specifically to the short-term analysis of breakpoints and trend shifts before and after the detected changepoints. This section highlights that no statistically significant or abrupt nonstationarities were found in the precipitation time series during this short period, even though slight declining trends are visually noticeable.

In contrast, lines 280–284 state: "In summary, based on the results presented in this section (Figures 4 and 5), the onset of severe and prolonged droughts in North Africa can be traced back to the mid-1990s. Since 1998, the region has experienced sustained drying. During this period, three major droughts have been identified, with the current ongoing drought, beginning in 2019 and reaching record-breaking severity in 2021, representing the most critical in terms of spatial extent and historical intensity."

and lines 382–388 add: "The results of the study indicate that since 1998, the entire North African region has been experiencing continuous drying, marked by decreasing precipitation and rising temperatures. The findings suggest that in recent years, particularly since 2021, an exceptionally severe drought has emerged and is still ongoing in 2024, an event unprecedented in the past 60 years. In this context, Tunisia, Algeria, and Morocco are the most critically affected countries in terms of both the extent and severity of meteorological drought, experiencing critical to extreme conditions."

By contrast, lines 280–284 and 382–388 discuss the long-term evolution of drought conditions based on the SPEIbase dataset, which spans approximately 70 years (1950–2024). Here, the analysis focuses on multi-decadal variability, identifying sustained drying across the entire North African region since the late 1990s. From a statistical perspective, it is fully plausible for short-

term precipitation series to exhibit no significant trend, while the derived long-term drought indices, which integrate cumulative climatic effects, display clear and significant drying trends. This distinction underlines the necessity of using standardized drought metrics rather than relying solely on short-term precipitation data. Thus, the apparent difference arises because the precipitation trend analysis (short-term, 1998–2024) focuses on identifying abrupt changes or structural breaks, while the drought-index analysis (long-term, 1950–2024) captures cumulative climatic drying trends across decades. This difference underscores the necessity of using standardized drought indices rather than relying solely on short-term precipitation trends, as has also been shown in other studies. Accordingly, we revised the relevant passage in the manuscript to clearly distinguish these analytical scales and avoid misinterpretation.

Edited manuscript (lines 302-317):

"It is noteworthy that the breakpoints detected in a few pixels in Fig. 2 may not be spatially extensive enough to achieve statistical significance at broader scales, such as the national level. For example, as shown in Fig. 2a, precipitation exhibits localized significant breakpoints in parts of Egypt and Algeria; however, this does not necessarily imply corresponding, statistically significant changes in the drought indices for those areas. To address these spatial limitations and the paucity of in situ observations, we drew on multiple open data sources, including satellite-based precipitation datasets (Fig. 3). Figure 3 presents the monthly precipitation time series for each of the five countries. As indicated, no significant trend except light visually trend discernible isn't apparent, and both the BEAST and MMK tests confirm the absence of statistically significant breakpoints or changepoints in any precipitation series. By contrast, as discussed in the following section, the drought indices reveal distinct long-term patterns, including statistically significant trends and breakpoints, that provide deeper insight into the region's hydroclimatic variability.

These findings are consistent with previous studies that did not identify substantial precipitation declines or statistically significant breakpoints across NA, yet documented episodes of severe drought, particularly during 1998–2001[4-7]. Accordingly, the next section examines drought behavior using multiple indices derived from diverse datasets. Moreover, unlike earlier research that focused primarily on meteorological indicators, our analysis integrates satellite-based groundwater storage variations with conventional drought metrics, providing a more comprehensive perspective that links climatic variability with anthropogenic influences."

1.8- Table 2 shows extremely large Z values (e.g., 35.0) and Sen's slope as a percentage (e.g., 5.6%). The meaning of these percentages and how they were calculated (per cent of what baseline?) is not defined. Also, the p-value is shown as "0.0000" without precision.

Response:

The authors would like to thank the reviewer for having pointed out this missing information and the lack of clarity of the text. The Z values were recalculated using the variance-corrected formulation that accounts for autocorrelation within the time series, resulting in moderate

magnitudes (|Z| ≤ 6.2). The percentage values now represent the relative magnitude of Sen's slope compared to the mean, thus providing a normalized indication of the rate of change. The p-values are now reported with four-decimal precision.

1.9- The text says no clear trends at the country level and BEAST/MK confirm no significant trends in some series (lines 234–240), yet claims an unprecedented drought beginning in 2019 that "intensified each year since" (lines 249–267) and asserts continuous drying since 1998 (lines 280–284, 382–388). Which is it? Localised vs regional? The statements conflict.

Response: The authors understand that the information above might sound confusing. However, as explained earlier in Comment 1.7, there is no contradiction. The apparent inconsistency arises because the variables, spatial scales, time spans, analytical frameworks, and datasets referenced in these sections are entirely different.

Specifically, lines 234–240 refer to: "It is worth noting that these changes are not spatially extensive enough to be statistically significant at larger scales, such as the national level... Figure 3 shows the monthly precipitation time series for each of the five countries. As illustrated, no clear trends are visually apparent, and the results of both the BEAST and MK tests confirm the absence of statistically significant trends or change points in any of the time series."

This part refers to the short-term breakpoint and trend analysis of monthly precipitation (1998–2024) at the national scale. The objective here was to identify abrupt shifts or nonstationarities in recent precipitation patterns, and the results indicated no statistically significant breakpoints at this scale during that limited period.

In contrast, lines 280–284 and 382–388 describe the results derived from the long-term drought indices (SPEI-12, SPEI-24, and SPEI-48) for the 1954–2024 period, focusing on the regional scale across Tunisia, Algeria, and Libya. These analyses capture multi-decadal cumulative trends rather than short-term precipitation variability. Thus, while the precipitation data show no significant short-term change, the drought indices, integrating long-term climatic deficits, reveal a persistent and intensifying drying trend since the late 1990s, culminating in the unprecedented drought beginning in 2019 and continuing through 2024. Each analysis must therefore be interpreted within its respective temporal and spatial context. However, when integrated, these complementary results form a coherent narrative: local and short-term precipitation variations (1998–2024) do not contradict, but rather support the broader pattern of long-term regional drying identified through multi-decadal drought indicators.

To further clarify this relationship and reduce the risk of misinterpretation, we revised the relevant text accordingly. In addition, ERA5-based drought indices were incorporated into the revised manuscript to provide a more comprehensive and realistic assessment of regional hydroclimatic conditions.

Edited manuscript (lines 332-349):

"This temporal behavior is corroborated by ERA5 reanalysis data (Fig. 5). Figures 5a–c presents the national-scale time series of SPI-12 and SPI-48 derived from ERA5 for Algeria, Libya, and Tunisia, respectively, while Figures 5d–e displays the spatial distribution of both indices across North Africa for March 2024. The national-scale results (Fig. 5a–c) confirm the occurrence of intense and unprecedented droughts during 2021–2024 (red dashed circles) and near-normal conditions between 2001 and 2010 (green rectangles). Because ERA5 products are partly constrained by GHCN ground-based observations, their temporal behavior closely aligns with the station-based SPI results (Fig. 4 and Supplementary Material). As shown in Figures 5d–e, the selected accumulation period (or timescale) plays a critical role in interpreting spatial SPI results and must be evaluated jointly, as it can substantially alter the spatial classification of drought severity even for the same date. For instance, in Tunisia, the SPI-12 map for March 2024 (Fig. 5d) classifies the country as experiencing moderate drought, whereas the SPI-48 map (Fig. 5e) categorizes nearly the entire territory under extreme drought, consistent with widespread aridity across NA. This comparison underscores the necessity of analyzing multiple accumulation periods simultaneously to capture both short- and long-term drought dynamics. Also, integrating multiple data sources is likewise essential for a comprehensive understanding of the spatial and temporal variability of drought conditions across complex climatic regimes.

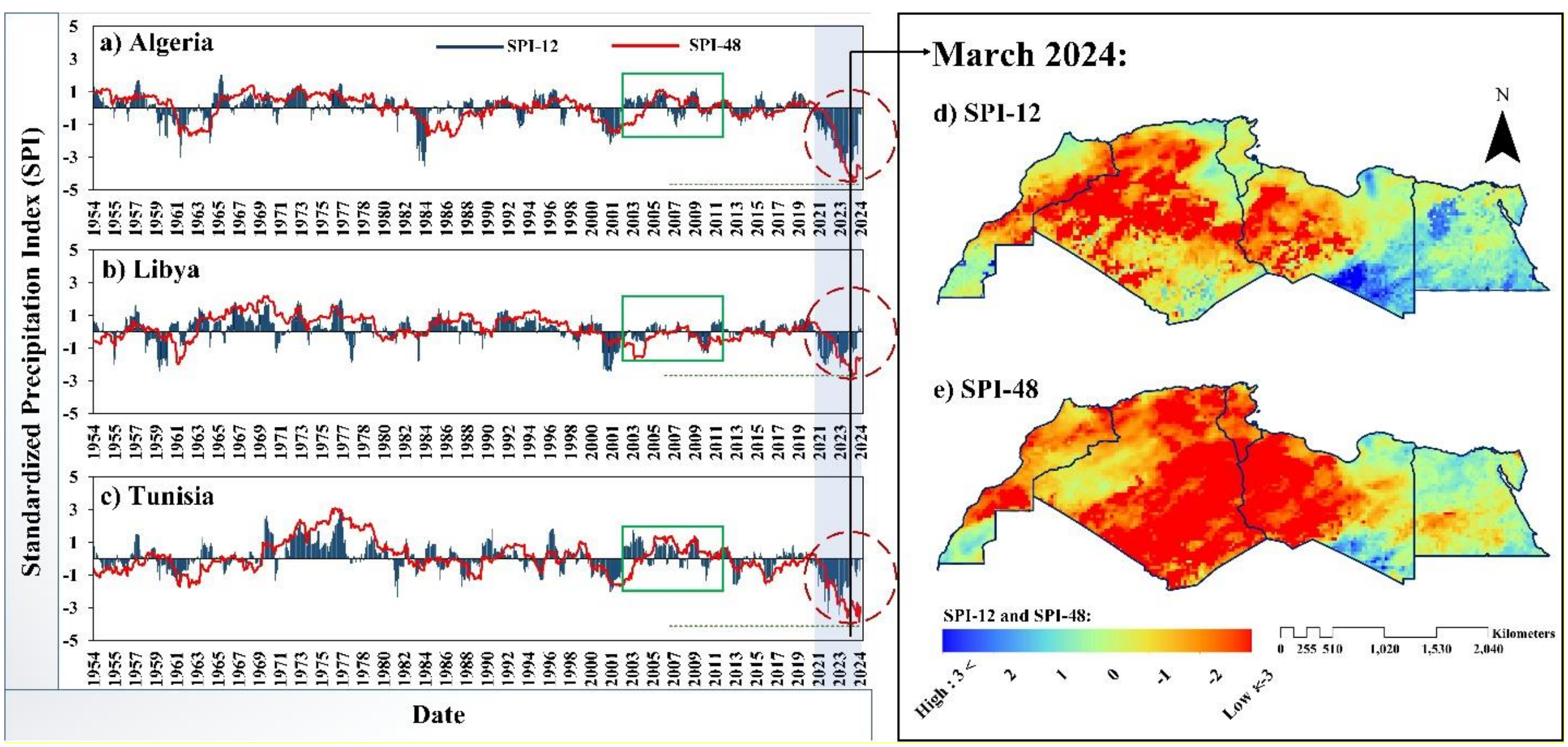

Fig. 5. Time-series variations of SPI-12 and SPI-48 based on ERA5 data at the national scale for Algeria, Libya, and Tunisia (a, b, c), and spatial distribution maps of both accumulation period (-12 and -48) across North Africa for March 2024 (d, e)."

1.10- The groundwater decline in Tunisia is linked to human extraction (lines 346–351, 374–381) despite the manuscript admitting a lack of access to observational data and heavy reliance on remote sensing (lines 54–56, 94–101). The evidence is suggestive but not sufficient for attribution.

Response: As mentioned early (comment 1.6), this is precisely the core motivation of the present study: to evaluate how, despite the lack of access to in situ groundwater data, remote sensing products can be used to reveal spatiotemporal patterns of change with the lowest possible uncertainty.

Recently, satellite-based observations have made it feasible to assess hydrological variability even in regions where direct measurements are inaccessible, which justifies the relevance and contribution of this study. As stated throughout the manuscript (e.g., lines 54–56, 94–101), we explicitly acknowledge the absence of direct groundwater abstraction data and our reliance on satellite-based indicators. It is important to emphasize that our objective was not to estimate precise groundwater withdrawal rates or compute exact groundwater level changes. Rather, our goal was to identify and document significant spatiotemporal variations and to discuss their most plausible explanations through comparative hydrological evidence.

Indeed, as the reviewer notes, "the evidence is suggestive" and that is the intended scope of this study. The post-2010 decline in groundwater storage (GWS) observed in Tunisia appears only in the GRACE and GRACE-DA datasets for that country, but not in any of its neighboring states, under the same climatic and methodological conditions. Given the absence of other regional mechanisms (e.g., snowmelt, ice, or vegetation changes), the most reasonable explanation for this isolated and abrupt decline is intensified human groundwater extraction. Nevertheless, we refrained from making any definitive attribution. All interpretations in the manuscript are carefully phrased as evidence-based indications, not deterministic statements. This caution is evident in several key excerpts:

Lines 343–351: "Several factors may contribute to this disparity… However, when comparing Figures 7 and 8 with the plots in the previous sections (Figures 3, 4, and 5), it becomes evident that meteorological drought alone cannot account for the observed hydrological drought in Tunisia. Precipitation levels between 2002 and 2010 were relatively favorable, yet a marked breakpoint and sharp decline in groundwater storage appear around 2010–2011 (Figure 7, purple curve). Thus, it can be concluded that the hydrological drought in Tunisia cannot be solely attributed to meteorological factors. Instead, anthropogenic influences likely play a key role in this dramatic decline."

Edited manuscript (lines 435-445):

"Several factors may account for this discrepancy. The first concerns differences in spatial scale, which are critical in explaining the contrasting drought behaviors observed in Tunisia relative to other North African countries. As illustrated in Fig. 1, Tunisia is geographically smaller than its neighbors, such as Libya and Algeria. Consequently, droughts driven by climatic variability in the

Mediterranean region often encompass the entire Tunisian territory, whereas only portions of the much larger Algeria, Morocco, or Libya are affected. However, comparison of Fig. 7 and Fig. 8 with the plots presented earlier (Figs. 3, 4, and 5) clearly indicates that meteorological drought alone cannot account for the hydrological drought observed in Tunisia. Between 2002 and 2010, precipitation remained relatively favorable (Fig. 3a), and drought conditions were largely normal (green rectangles in Figs. 4e–h and 5c). Nevertheless, a distinct breakpoint and sharp decline in groundwater storage appear around 2010–2011 (purple curve in Fig. 7). These findings suggest that the hydrological drought in Tunisia cannot be attributed solely to meteorological drivers; rather, anthropogenic factors likely played a significant role in intensifying this pronounced decline."

As mentioned, two of the co-authors are Tunisian researchers residing in Tunisia and are directly familiar with local water management practices. Their field experience supports the plausibility of human-driven groundwater depletion, yet we deliberately avoided asserting causality and instead presented these as observational indications.

Moreover, the Discussion section explicitly continues this cautious framing (lines 351–352):

"The discussion section examines the underlying causes of this phenomenon in Tunisia in greater detail."

Finally, in response to this helpful comment, we revised the manuscript title to more accurately reflect the scope and strength of evidence, changing it from:

"Spatiotemporal hydrometeorological drought variations in North Africa: Amplified Drought Linked to Anthropogenic Influences"

to:

Edited manuscript (1-2): "Spatiotemporal hydrometeorological drought variations in North Africa: Indications of Anthropogenic Contributions to Amplify Drought."

This modification ensures that the phrasing conveys suggestive but not conclusive attribution, fully addressing the reviewer's concern.

1.11- In lines 98–105, the manuscript notes that 280 GHCN stations were available and that 76 with more than 30 years of data were selected, but the criteria for "high quality" versus "low quality" remain vague. It is not clear whether the GHCN station series were actually used for trend tests or only shown in descriptive plots. The authors should clearly specify the selection process (e.g., minimum record length, completeness thresholds, quality flags), provide the list of station IDs used in analyses, and state explicitly which stations were included in statistical tests versus which were excluded. If in-situ data were not used for validation or formal testing, this needs to be stated plainly so as not to give the impression of independent observational support where none was applied.

Response: We appreciate the reviewer's valuable comment and agree that the quality criteria for GHCN station selection required clearer definition. Although this was implicitly reflected in Figure 1, the criteria were not explicitly stated in the text.

Edited manuscript (104-109):

"Due to the limited spatial coverage and uneven distribution of GHCN stations across North Africa, no spatial interpolation of the station time series was performed. Instead, all analyses were conducted on a point-to-grid basis. The GHCN observations were used both to validate the SPI-based drought indices and to perform independent trend analyses using in situ series. Comprehensive plots showing the trend, seasonal, and residual components for all 131 active stations (by station name), as well as SPI time series for 35 selected stations (red point at Fig.1), are provided in the Supplementary Files."

Edited manuscript (244-246):

Among these, 35 stations meeting four rigorous criteria, (1) the longest continuous observation records, (2) minimal data gaps, (3) stable instrumentation and consistent measurement protocols, and (4) uninterrupted reporting up to the present, were classified as best-quality stations."

Additionally, we have included in the Supplementary Files detailed metadata and SPI results for all GHCN stations. This addition not only addresses the reviewer's concern but also significantly improves transparency and reproducibility for future studies in data-scarce regions.

1.12- The GRACE-DA-DM product percentiles are said to be based on historical records from 1948–2014 (line 121). Yet analyses extend to 2024 (e.g., Fig.7 lines 331–341). Explain how percentiles and baselines were computed beyond 2014 and how you addressed differences between CSR and JPL signals. Please provide a sensitivity analysis using both products.

Response: We thank the reviewer for raising this important point. It is worth clarifying that in our analyses we did not directly derive groundwater storage (GWS) from the raw GRACE CSR or JPL solutions. Nowhere in the manuscript do we attempt to reconstruct GWS from the mascon

products. Instead, as stated in the text, all groundwater-related analyses (Figure 8 and Table 2) rely on the GRACE-Data Assimilation for Drought Monitoring (GRACE-DA-DM) product.

This assimilation framework integrates GRACE/GRACE-FO observations with ground- and satellite-based meteorological forcing data into a physically based land-surface model, yielding spatially and temporally continuous estimates of soil moisture, groundwater, and other hydrological fluxes. From these modeled states, three drought indicators are generated, groundwater, root-zone soil moisture, and surface soil moisture, each expressed as percentiles relative to a historical climatology (1948–2014). These percentiles represent the probability that the current condition is equal to or drier than the corresponding week of the reference record.

To extend the time series beyond the 1948–2014 baseline, we followed the standard procedure used by the product developers: for each grid cell and variable, values after 2014 were expressed as relative deviations from the historical mean and variance computed over 1948–2014. In other words, post-2014 anomalies are referenced to the long-term climatological distribution but do not alter the baseline itself.

For example, the Groundwater Storage Percentage (GWS %) index is calculated as:

$$\mathrm{GWS\%} = \frac{\mathrm{GWS}_t - \mathrm{G\bar{W}S}}{\mathrm{G\bar{W}S}} \times 100 \quad (1)$$

where GWS_t denotes the groundwater storage at time t(e.g., 2024) and $\mathrm{G\bar{W}S}$is the long-term mean groundwater storage over 1948–2014. This index quantifies the relative deviation of groundwater storage from its climatological mean, providing an effective measure for assessing temporal fluctuations and the magnitude of groundwater depletion or recovery.

Note: the number of figures had been changed, Fig. 8. (Fig.7 lines 331–341) Time series of groundwater storage percentiles for the five individual countries and the area-averaged value across all countries (in red) in North Africa (2003–2024).

1.13- Typos and inconsistent naming: e.g., "metrological" vs "meteorological" repeated (line 200 et seq.), inconsistent SSP notation (SSP2_4.5 vs SSP2-4.5) — tidy throughout. (Multiple places: lines 200–210, lines 270–285).

Response: We thank the reviewer for noting these inconsistencies. The typographical error "metrological" has been corrected to "meteorological" in both instances identified.

Regarding the reviewer's observation about inconsistent SSP notation ("SSP2_4.5" vs. "SSP2-4.5"), we carefully rechecked the entire manuscript to make sure the notation is consistent. The entire manuscript has been re-reviewed to ensure consistent terminology and notation throughout.

1.14- Overstatements in Abstract/Conclusion: Abstract claims precise magnitudes (SPEI drop from −0.26 to −2.27) and "average decline exceeding 6% across all indices" (lines 27–36) without uncertainty or a method for computing "average decline in %". Provide CI and method.

Response: We appreciate the reviewer's insightful comment. The reported numerical values in the Abstract (e.g., the SPEI decrease from −0.26 to −2.27 and the average decline of about 6% across all indices) were directly obtained from the statistical analyses applied to the three reference periods using the standard procedures outlined in the Methods section.

To enhance transparency and address the reviewer's concern, the full SPEIbase datasets and corresponding statistical outputs have been provided in the Supplementary Information, enabling independent verification of all reported magnitudes.

Additionally, the Abstract has been revised to remove specific numerical claims and to present the results in a more general and balanced manner.

Links: SPEIbase_Data_and_Spatiotemporal_variations/ trend analysis and identifying breakpoints [https://figshare.com/s/57248f9d919bf1f46eab].

2) Reviewer #2:

This study looks at how droughts have changed over time in North Africa, especially in Tunisia, Algeria, and Morocco. It shows that since 1998, the region has been getting drier, with less rainfall and higher temperatures. A particularly severe drought started around 2021 and is still ongoing in 2024 which is the worst in the past 60 years. Authors attributed these droughts to climate change and added that human dependence on groundwater, are making things worse, especially in Tunisia. The study mentions that looking at just one type of drought (like rainfall) isn't enough. A multi-dimensional approach considering both weather and water use is needed to understand and manage droughts better. I have the following comments on the manuscript for authors consideration:

2.1- Section 2.4 can be avoided by simply citing the testes/parameters/indicators used, and keeping only the explaining how these indicators (Bayesian time-series decomposition using BEAST, The Mann–Kendall test, and the Sen's slope) are used for the study.

Response: We thank the reviewer for this helpful suggestion. In the revised version, Section 2.4 has been condensed to remove redundant theoretical background and now focuses primarily on the methodological and practical aspects of the applied techniques, particularly the BEAST algorithm. The detailed mathematical formulations, especially the equations for the MMK test and Sen's slope estimator, have been moved to the Supplementary Information, where interested readers can consult them for technical depth.

2.2- Line 206, 208: phrase 'this figure' used can cause confusion please try and be specific and indicate the figure number to avoid ambiguity.

Response: We thank the reviewer for this clarification. All generic references such as "this figure" have been replaced with explicit figure numbers throughout the manuscript to eliminate ambiguity and improve readability.

2.3- Line 202: 'section e' please add the full figure number with the panel. Please be consistent with the terminology, 'section' here was earlier mentioned as 'parts' in line 205.

Response: We thank the reviewer for the thorough review and apologize for the editing blunders. We believe a minor oversight occurred, as the reference in the previous version was located in line 220 (not line 202). Specifically, the phrase "section e" has now been replaced with the complete figure and panel reference ("Fig. 2e"). In addition, the entire manuscript has been carefully reviewed to ensure consistent terminology across all figure references, maintaining uniform usage of "Fig." and panel labels, and removing inconsistent terms such as "section," "panel," or "part."

2.4- Line 202: 'other maps', please be specific about which maps you are comparing.

Response: We thank the reviewer for pointing out this issue. The text has been revised to explicitly identify the maps being compared, which now reads "other maps (Fig. 2a and c)" in the manuscript.

2.5- Section 3.1.1 while mentioning the comparison of maps, please mention how the maps were compared and what were the indicators used for the quantification of differences/similarities between the maps.

Response: We thank the reviewer for raising this valuable point. The methodology section has been revised to clarify how the maps were compared, and which indicators were used to quantify spatial differences and similarities. The following paragraph has been added to the edited manuscript (Lines: 205-216):

"In BEAST, each pixel is analyzed independently as a separate time series. For each series, the model allows up to five changepoints, producing up to five posterior probabilities, five years of occurrence, and the corresponding segment parameters (e.g., slopes and intercepts). The algorithm directly computes the posterior inclusion probability for each potential changepoint, meaning that each year (or time step) receives an explicit posterior probability of being an abrupt change. In this framework, no fixed probability threshold is required, because all changepoints are ranked and reported according to their posterior probability. The changepoint with the highest posterior probability (typically > 0.90–0.95 in our analysis) represents the most likely abrupt change within that pixel. This value inherently conveys the confidence level of detection. By comparing the corresponding trend slope at that changepoint, we can determine whether the change was positive or negative and identify its exact year. Accordingly, instead of applying a global cutoff, we compared two complementary maps: Slope of abrupt change, showing the magnitude and direction of the detected shift, and Probability of abrupt change, showing the corresponding posterior probability. Together, these two maps provide a complete spatial representation of where, when, and with what intensity abrupt changes occurred."

2.6- Line 230-231: 'These findings are consistent with results from other studies using different data sources', please a small section or few lines be added here explaining the similarities and differences with other studies cited.

Response: We appreciate this constructive suggestion. A concise comparative discussion has been added to clarify how our findings align with and differ from previous studies. Specifically, the new text emphasizes that while earlier works primarily documented episodes of severe drought between 1998 and 2001, without detecting statistically significant breakpoints or pronounced precipitation declines across North Africa, the present study extends these analyses by integrating satellite-derived groundwater storage variations and anthropogenic dimensions to provide a broader hydroclimatic context.

edited manuscript (lines 312–317):

"These findings are consistent with previous studies that did not identify substantial precipitation declines or statistically significant breakpoints across NA, yet documented episodes of severe drought, particularly during 1998–2001[4-7]. Accordingly, the next section examines drought behavior using multiple indices derived from diverse datasets. Moreover, unlike earlier research that focused primarily on meteorological indicators, our analysis integrates satellite-based groundwater storage variations with conventional drought metrics, providing a more comprehensive perspective that links climatic variability with anthropogenic influences."

2.7- Figure 3 and section 3.1.1: please explain how the time series are produced using the gridded data for better clarity.

Response: We appreciate the reviewer's insightful comment. To enhance clarity, we added a new subsection titled "2.5. Spatiotemporal processing of multi-source datasets" in the methodology, which describes all spatial and temporal procedures applied throughout the study, including those used to generate Fig. 3. The time series in Fig. 3 were produced by spatially averaging monthly precipitation values from the GPM IMERG product within each country's administrative boundaries. The averaging was performed using area-weighted means based on the proportion of grid-cell coverage within national borders. This procedure ensures that each data point represents the national mean precipitation for a given month, providing consistent and comparable estimates across variables and countries.

Edited manuscript (line 235-265):

"2.5. Spatiotemporal processing of multi-source datasets

Initially, the BEAST algorithm was applied to all ERA5 datasets, without altering their native spatial or temporal resolutions, to analyze pixel-wise time series of three variables (precipitation, temperature, and evapotranspiration) across NA for the period 1981–2024. Each pixel's time series was individually evaluated to identify climatic breakpoints based on both the slope and the probability of abrupt changes. Subsequently, monthly GPM precipitation data (1998–2024) were spatially averaged at the national scale for each of the five NA countries. The resulting time series were compared to assess inter-country differences in breakpoints and long-term trends. As in the ERA5 analysis, no artificial resampling or temporal adjustment was performed, except for spatial averaging of the gridded monthly data within each national boundary. Complementary analyses were performed using in-situ observations from 131 active meteorological stations (see Supplementary Material). Among these, 35 stations meeting four rigorous criteria, (1) the longest continuous observation records, (2) minimal data gaps, (3) stable instrumentation and consistent measurement protocols, and (4) uninterrupted reporting up to the present, were classified as best-quality stations. The SPI was computed for all selected stations over 1984–2024, and each point-based series was evaluated independently (Supplementary Material). To further characterize regional-scale drought evolution, ERA5-Drought datasets were employed to derive national-scale monthly SPI-12 and SPI-48 indices for each country over 1954–2024, followed by detailed time-series analyses. In parallel, the SPEIbase dataset was used to compute regional mean SPEI values

across Algeria, Tunisia, and Libya, treated as a single area zone, over four accumulation periods (1954–2024) to examine large-scale temporal patterns of drought intensity and persistence. For hydrological drought assessment, satellite gravimetry products were incorporated in a comparative framework to detect breakpoints and long-term variations across NA countries. TWS anomaly variations were computed at a seasonal scale for each pixel and analyzed using the BEAST algorithm for 2002–2024, preserving the native spatial resolution. Results were obtained separately for the CSR and JPL GRACE products. Finally, temporal trends in three key hydrological components, SFSM, RZSM, and GWS, were analyzed using the GRACE-DA-DM dataset for each country over 2003–2024, with particular focus on GWS depletion. In this regard, no datasets in this study were subjected to cross-product spatial or temporal fusion; standard regridding procedures were applied only when strictly necessary. For temporal analyses, data were aggregated to monthly, seasonal, or annual scales according to the comparison objectives. For national-scale spatial assessments, all variables were resampled to the finest common grid (0.1°) using the nearest-neighbor method, and spatial means were computed within national boundaries. A uniform boundary shapefile was used for all averaging procedures, with fractional weighting of boundary pixels according to their overlap with national polygons. Consequently, all spatial and temporal analyses were conducted in a methodologically consistent and internally coherent manner."

2.8- Figure 4 and section 3.1.2: it would be great if the locations of the stations mentioned in the figure 4 can be added to a map for context. It will me much of meaningful and add a great deal of value to the comparisons made.

Response: We sincerely thank the reviewer for this valuable suggestion. In Fig. 4, due to space limitations, only eight representative station plots were initially shown. Following this comment, we have now provided the complete set of time-series plots for all 35 high-quality stations, which are already displayed as red points across the study area in Fig. 1. These additional plots are now included in the Supplementary Information to enhance spatial context and allow readers to visually link each station's behavior to its geographic location.

Manuscript (lines 107–109):

"Comprehensive plots showing the trend, seasonal, and residual components for all 131 active stations (by station name), as well as SPI time series for 35 selected stations (red point at Fig.1), are provided in the Supplementary Files."

Linke: 35_Stations_SPI-3-6-9-12-24-48: [https://figshare.com/s/57248f9d919bf1f46eab]

2.9- Section 3.1.2 in figure 4 the graphs shown were of individual stations, comparing those with an area averaged series of three countries (figure 5) doesn't seems sensible. I think it would be interesting to see the comparison with the area averages series over the individual countries.

Response: We sincerely thank the reviewer for this valuable comment. Following this suggestion, an additional analysis was performed and incorporated into the revised manuscript. While we retained the original figure (previously Fig. 5, now renumbered as Fig. 6), we also added a new figure (Fig. 5) using an independent dataset (ERA5). This new analysis presents the comparison between the area-averaged drought indices for each individual country, rather than a regional composite. Specifically, for each country, the SPI-12 and SPI-48 indices were recalculated from the ERA5 dataset and compared to capture national-level drought dynamics. This addition enhances the spatial representativeness and aligns the analysis with the reviewer's recommendation for a country-based assessment of drought behavior.

Edited manuscript (lines 332-349):

"This temporal behavior is corroborated by ERA5 reanalysis data (Fig. 5). Figures 5a–c presents the national-scale time series of SPI-12 and SPI-48 derived from ERA5 for Algeria, Libya, and Tunisia, respectively, while Figures 5d–e displays the spatial distribution of both indices across North Africa for March 2024. The national-scale results (Fig. 5a–c) confirm the occurrence of intense and unprecedented droughts during 2021–2024 (red dashed circles) and near-normal conditions between 2001 and 2010 (green rectangles). Because ERA5 products are partly constrained by GHCN ground-based observations, their temporal behavior closely aligns with the station-based SPI results (Fig. 4 and Supplementary Material). As shown in Figures 5d–e, the selected accumulation period (or timescale) plays a critical role in interpreting spatial SPI results and must be evaluated jointly, as it can substantially alter the spatial classification of drought severity even for the same date. For instance, in Tunisia, the SPI-12 map for March 2024 (Fig. 5d) classifies the country as experiencing moderate drought, whereas the SPI-48 map (Fig. 5e) categorizes nearly the entire territory under extreme drought, consistent with widespread aridity across NA. This comparison underscores the necessity of analyzing multiple accumulation periods simultaneously to capture both short- and long-term drought dynamics. Also, integrating multiple data sources is likewise essential for a comprehensive understanding of the spatial and temporal variability of drought conditions across complex climatic regimes.

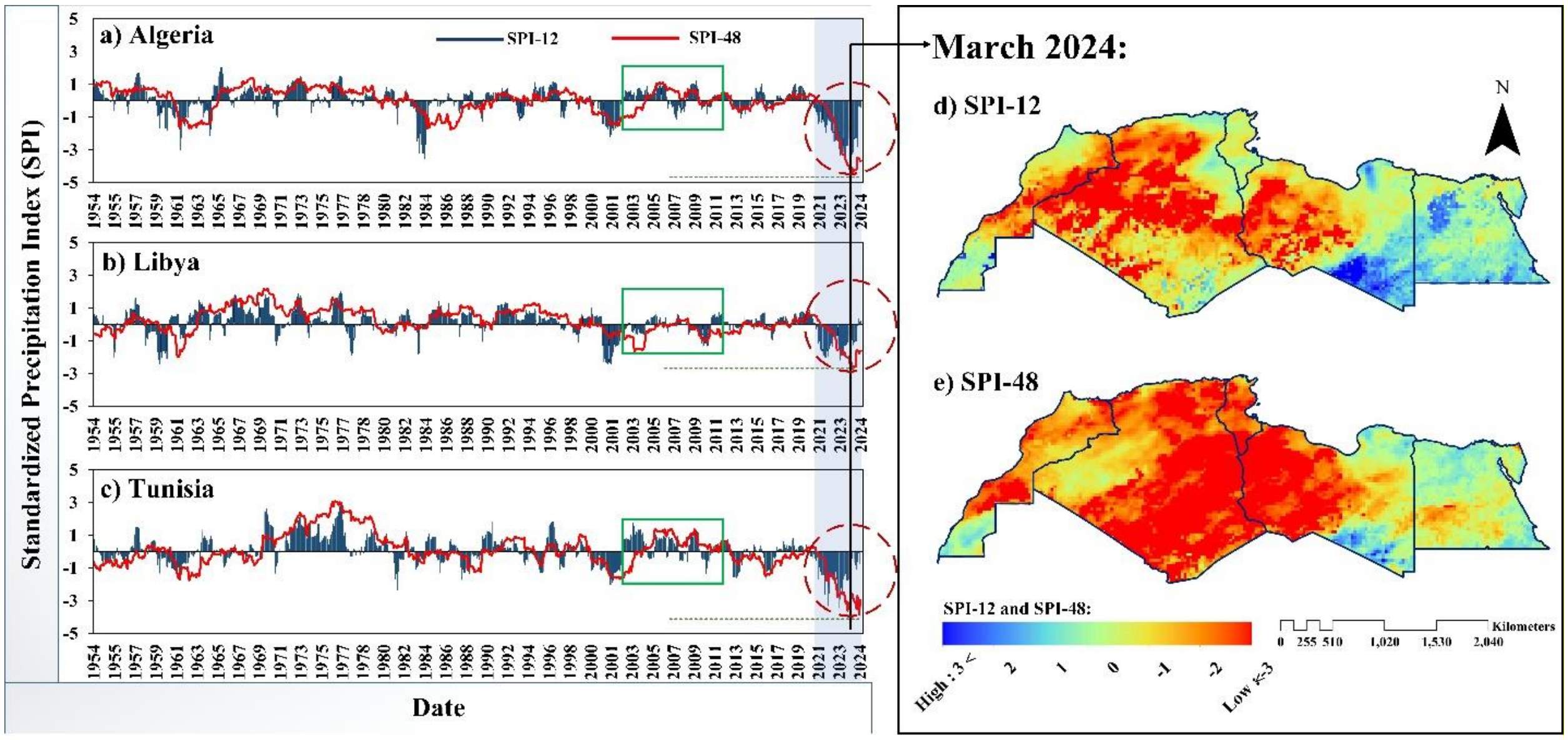

Fig. 5. Time-series variations of SPI-12 and SPI-48 based on ERA5 data at the national scale for Algeria, Libya, and Tunisia (a, b, c), and spatial distribution maps of both accumulation period (-12 and -48) across North Africa for March 2024 (d, e)."

2.10- Lines 295-316: majority of the section 3.2 should be the part of discussion section. The manuscript needs a bit of rearranging in terms of results and discussion sections.

Response: We appreciate this structural feedback and fully agree with the reviewer on this point. Considering the analytical nature of the study and the way results are interpreted, separating the discussion section from the results would create a duality between the hydroclimatic interpretation and the attributional analysis of GWS changes related to human influences. Therefore, we decided to present the Results and Discussion together as an integrated section. Due to the challenges involved in addressing the topic of Amplified Drought Linked to Anthropogenic Influences, it was necessary to dedicate a specific subsection to this theme. Keeping this subsection separate ensures that the main discussion remains focused and avoids distracting the reader with unrelated topics. Accordingly, we now present the paper with a unified Results and Discussion section, including a distinct subsection on Breakpoints and Indications of Anthropogenic Contributions to Drought Amplification.

2.11- The study mentioned that the results and finding the consistent with previous studies, please add a section in the discussion to explain the consistency, differences, nuances and novelty of this study in context with the existing literature.

Response: We sincerely thank the reviewer for this constructive suggestion. As previously mentioned, we revised the structure and headings of the Results and Discussion sections. Following this comment and Comment 2.6, we added a dedicated paragraph in Sections 3.1.1 and

3.3 of the revised manuscript. These new additions explain how our approach, by integrating remote sensing, reanalysis data, and GRACE-based groundwater indicators, extends the findings of previous studies to reveal the amplification of hydrological drought induced by human activities, an aspect that has rarely been addressed in earlier assessments across North Africa.

Edited manuscript (lines 312-317):

"These findings are consistent with previous studies that did not identify substantial precipitation declines or statistically significant breakpoints across NA, yet documented episodes of severe drought, particularly during 1998–2001[4-7]. Accordingly, the next section examines drought behavior using multiple indices derived from diverse datasets. Moreover, unlike earlier research that focused primarily on meteorological indicators, our analysis integrates satellite-based groundwater storage variations with conventional drought metrics, providing a more comprehensive perspective that links climatic variability with anthropogenic influences."

Edited manuscript (lines 466-469):

"These challenges, particularly in the aftermath of the unprecedented emerging drought (2021–2024) documented here for the first time, are expected to have profound and far-reaching consequences across all sectors of society. Marginalized farmers, whose livelihoods depend directly on groundwater availability, are likely to be the most severely affected[8]."

3) Reviewer #3:

This manuscript presents an interesting contribution in the study of drought in the Mediterranean region and I appreciate the interest to monitor not only meteorological drought but also hydrological. Due to the lack of meteorological data recorded at the ground the authors combined information belonging from different sources. However, several aspects of the manuscript would benefit from clarification, reorganization, and methodological improvement;

3.1- Mandatory revisions include:

Thank you very much for the thorough and constructive review of the manuscript and for all the comments.

3.1.1- Providing detailed information about the study area.

Response: All the requested details have been added to the Study Area section.

3.1.2- Revising figure color scales and labels for clarity.

Response: The authors are afraid that altering the color schemes and legends could potentially confuse readers and compromise the visual consistency of the figures. Some minor adjustments have been brought up in some of the figures. However, if the reviewer still prefers the adjustment, we will gladly implement the final modifications in the next version.

3.1.3- Reorganising the Results, Discussion, and Conclusion sections.

Response: Following the reviewer's recommendation, the overall structure has been revised. Given that the Discussion in this study forms an integral part of the analytical results, the authors proposed to merge the Results and Discussion sections to preserve narrative coherence and ensure smooth flow of interpretation.

3.1.4- Improving the description of the datasets, with particular attention to the methodology adopted for each and common study period.

Response: We fully agree with this valuable comment. The dataset descriptions and methodological details have been carefully revised and updated in accordance with this recommendation and those from other reviewers.

3.1.5- Recalculating SPI and SPEI indices using the ERA5-Land product to avoid comparing indices derived from different databases with varying temporal and spatial resolutions, which is methodologically incorrect.

Response: The authors thank the reviewer for this judicious comment. In line with this suggestion, we analyzed the SPI for two corresponding time scales (-12 and -48 month) using the ERA5 dataset and incorporated the resulting analyses into the Results section of the revised manuscript.

3.2- Study Area: Suggest that the authors provide more information on the study area to better address the drought issue, including details on: Geomorphology, Climatic characteristics, Vegetation distribution (cultivation and natural vegetation), Population distribution and water consumption

Response: This revision has been done.

Edited manuscript (lines 127-156):

"North Africa constitutes the northernmost region of the African continent, situated above the Sahara Desert and bordered by the Mediterranean Sea to the north. Although its exact boundaries vary across definitions, the region is generally considered to extend from the Atlantic coast of Morocco in the west to Egypt and the Red Sea coast of Sudan in the east. In this study, NA refers specifically to five countries, Morocco, Algeria, Tunisia, Libya, and Egypt, which collectively define the study domain (Fig. 1). These countries share strong geographic proximity, comparable climatic and hydrological regimes, and similar demographic, political, social, cultural, and economic characteristics[4].

The climate of NA is predominantly Mediterranean along the narrow coastal belts of Morocco, Algeria, Tunisia, and parts of Libya, where the mean annual precipitation ranges between 400- and 600-mm yr^{-1}. Inland regions exhibit semi-arid climates (200–400 mm yr^{-1}) that gradually transition to arid desert zones receiving less than 100 mm yr^{-1} of rainfall[4,9,10]. Three major physiographic features govern the regional climate system: the Mediterranean Sea to the north, the Sahara Desert to the south, and the Atlas Mountains to the west. From a geomorphology the Atlas Mountains extend across northern Morocco, Algeria, and Tunisia, reaching their highest elevation in central Morocco[9]. From a limnological and hydrological perspective, the region exhibits distinctive characteristics. The hydrology of NA is defined by several major river systems that sustain human and ecological life in an otherwise dry and semi-arid environment. The most important of these include the Nile, Medjerda, Moulouya, Oued Draa, and Oum Er-Rbia rivers, each playing a crucial role in supporting agriculture, urban settlements, and ecosystems across Egypt, Sudan, Tunisia, Algeria, and Morocco[4,9]. The Nile River, the longest river in the world, forms the backbone of Egypt's economy and food security, while smaller rivers such as the Medjerda in Tunisia and the Moulouya in Morocco remain vital to local economies and regional irrigation networks[9].

The total area of the study domain covers approximately 5.8 million km² and hosts about 223.6 million inhabitants, equivalent to roughly 2.8 % of the global population. A substantial share of this population resides in rural areas, where livelihoods depend directly on agriculture and water availability[4,9]. Consequently, variations in climate and the occurrence of droughts have immediate implications for water accessibility, agricultural productivity, and socioeconomic stability. Understanding spatiotemporal hydro-meteorological drought variations across this region is therefore essential, as such analyses not only clarify drought dynamics but also provide critical insight into broader challenges related to agriculture, water security, and societal resilience. However, this endeavor is constrained by limited observational networks, data scarcity, and restricted data accessibility, which collectively hinder regional climate and hydrological

assessment. These limitations underscore the necessity of the present research, which seeks to analyze hydro-meteorological drought events in a region where livelihoods and food security remain tightly interlinked with climate variability and water-resource availability."

3.3- Material and Methods: The authors have adopted multiple datasets encompassing ground-based observations, reanalysis products, and remote sensing sources. It is recommended that the authors improve the description and methodological clarity regarding each dataset, providing sufficient detail to ensure the methodology flow.

Response: The authors agree with the reviewer and would like to thank them for this comment. The Materials and Methods section has been thoroughly revised based on this and other reviewers' comments. Redundant equations have been removed, and greater emphasis has been placed on methodological flow. A step-by-step workflow is now provided to enhance description and methodological clarity for each dataset. Specifically, a new subsection titled "2.5. Spatiotemporal processing of multi-source datasets" has been added, where we clearly describe the preprocessing, temporal aggregation, unit conversion, regridding, and the role of each dataset in specific analyses (trend detection, changepoint analysis, and correlation.

Edited manuscript (line 235-265):

"2.5. Spatiotemporal processing of multi-source datasets

Initially, the BEAST algorithm was applied to all ERA5 datasets, without altering their native spatial or temporal resolutions, to analyze pixel-wise time series of three variables (precipitation, temperature, and evapotranspiration) across NA for the period 1981–2024. Each pixel's time series was individually evaluated to identify climatic breakpoints based on both the slope and the probability of abrupt changes. Subsequently, monthly GPM precipitation data (1998–2024) were spatially averaged at the national scale for each of the five NA countries. The resulting time series were compared to assess inter-country differences in breakpoints and long-term trends. As in the ERA5 analysis, no artificial resampling or temporal adjustment was performed, except for spatial averaging of the gridded monthly data within each national boundary. Complementary analyses were performed using in-situ observations from 131 active meteorological stations (see Supplementary Material). Among these, 35 stations meeting four rigorous criteria, (1) the longest continuous observation records, (2) minimal data gaps, (3) stable instrumentation and consistent measurement protocols, and (4) uninterrupted reporting up to the present, were classified as best-quality stations. The SPI was computed for all selected stations over 1984–2024, and each point-based series was evaluated independently (Supplementary Material). To further characterize regional-scale drought evolution, ERA5-Drought datasets were employed to derive national-scale monthly SPI-12 and SPI-48 indices for each country over 1954–2024, followed by detailed time-series analyses. In parallel, the SPEIbase dataset was used to compute regional mean SPEI values across Algeria, Tunisia, and Libya, treated as a single area zone, over four accumulation periods (1954–2024) to examine large-scale temporal patterns of drought intensity and persistence. For

hydrological drought assessment, satellite gravimetry products were incorporated in a comparative framework to detect breakpoints and long-term variations across NA countries. TWS anomaly variations were computed at a seasonal scale for each pixel and analyzed using the BEAST algorithm for 2002–2024, preserving the native spatial resolution. Results were obtained separately for the CSR and JPL GRACE products. Finally, temporal trends in three key hydrological components, SFSM, RZSM, and GWS, were analyzed using the GRACE-DA-DM dataset for each country over 2003–2024, with particular focus on GWS depletion. In this regard, no datasets in this study were subjected to cross-product spatial or temporal fusion; standard regridding procedures were applied only when strictly necessary. For temporal analyses, data were aggregated to monthly, seasonal, or annual scales according to the comparison objectives. For national-scale spatial assessments, all variables were resampled to the finest common grid (0.1°) using the nearest-neighbor method, and spatial means were computed within national boundaries. A uniform boundary shapefile was used for all averaging procedures, with fractional weighting of boundary pixels according to their overlap with national polygons. Consequently, all spatial and temporal analyses were conducted in a methodologically consistent and internally coherent manner."

3.4- For example, at line 100, the manuscript states that precipitation time series were employed to compute the SPI index. However, the drought time scale applied (e.g., 12-, 24-, or 36-month) is not specified. Moreover, the common period of study of the ground-based precipitation series remains unclear.

Response: Using the ground-based observational data, the SPI index was computed for multiple accumulation periods (3-, 6-, 12-, 24-, and 48-month time scales). However, as the overall temporal behavior across these scales was consistent and did not yield additional interpretive value, we presented only the SPI-12 results in the main text as a representative example.

To ensure transparency and reproducibility, all computed SPI indices for the 35 selected stations have now been made available in the Supplementary Files.

Linke: 35_Stations_SPI-3-6-9-12-24-48: [https://figshare.com/s/57248f9d919bf1f46eab]

Sample, for station GAFSA (TSM00060745):

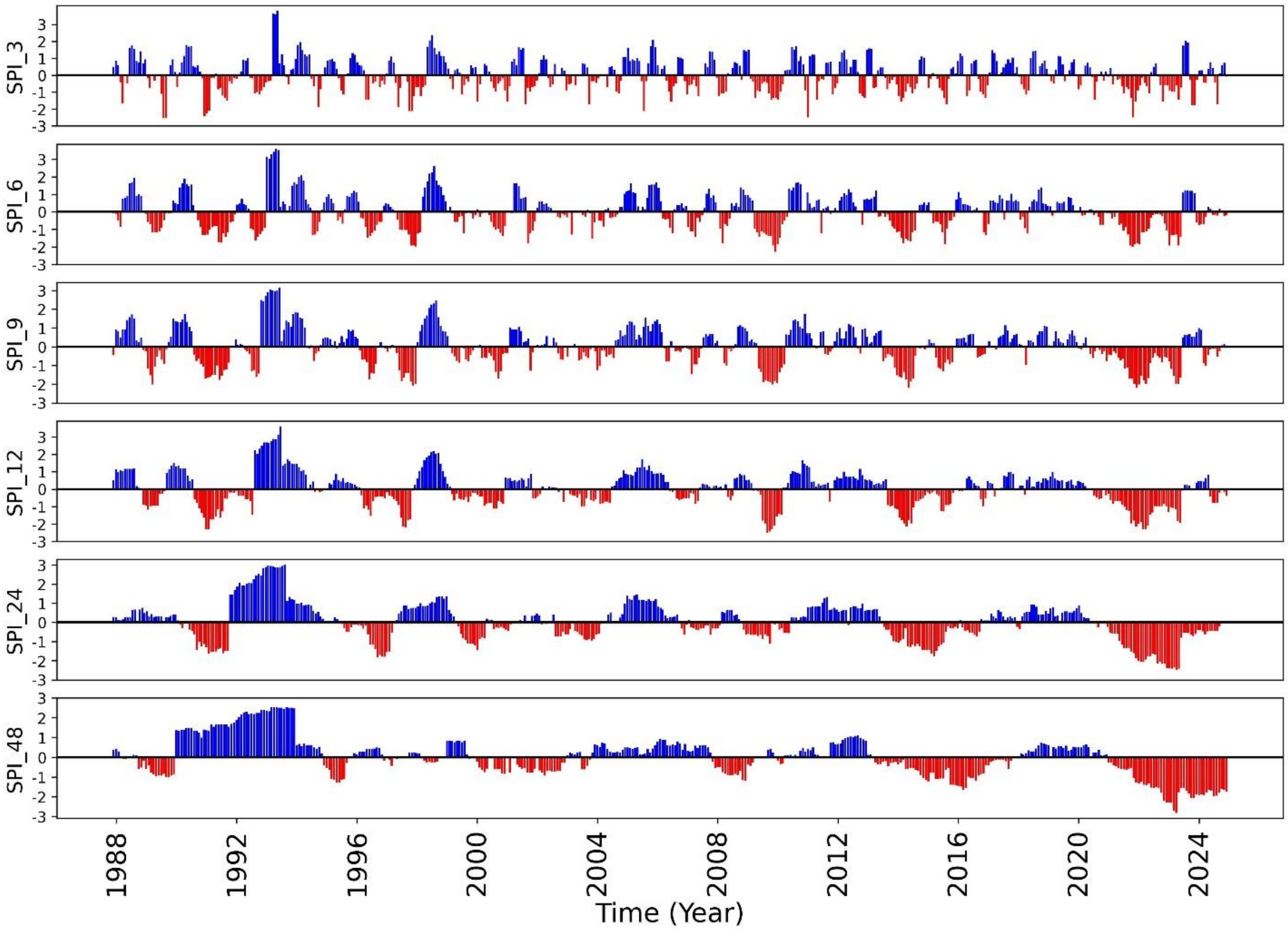

Additionally, the Data and Methodology section, including line 100, has been thoroughly revised to specify that the SPI-12 values were calculated for all observational stations over the period 1984–2024.

Edited manuscript (line 97-99):

"In the present study, the latest version (Version 3) of the Global Historical Climatology Network (GHCN) monthly precipitation dataset [11] was used to calculate and investigate SPI-12 across NA (see metadata in Table S1 and Supplementary Files)."

3.5- Going through the paper in the result section, the authors also analyse the SPEI index, which is derived from the SPEI Global Drought Monitor at a spatial resolution of 0.5°. Comparing indices computed from different data sources, at different spatial resolution and time period, represents a major methodological inconsistency that may compromise the robustness of the results. It is therefore strongly recommended that both SPI and SPEI indices be recalculated using the ERA5-Land database to ensure methodological consistency and reliability of the comparative analysis.

Response: The authors thank the reviewer for the judicious comment. Done.

Edited manuscript (lines 332-349):

"This temporal behavior is corroborated by ERA5 reanalysis data (Fig. 5). Figures 5a–c presents the national-scale time series of SPI-12 and SPI-48 derived from ERA5 for Algeria, Libya, and Tunisia, respectively, while Figures 5d–e displays the spatial distribution of both indices across North Africa for March 2024. The national-scale results (Fig. 5a–c) confirm the occurrence of intense and unprecedented droughts during 2021–2024 (red dashed circles) and near-normal conditions between 2001 and 2010 (green rectangles). Because ERA5 products are partly constrained by GHCN ground-based observations, their temporal behavior closely aligns with the station-based SPI results (Fig. 4 and Supplementary Material). As shown in Figures 5d–e, the selected accumulation period (or timescale) plays a critical role in interpreting spatial SPI results and must be evaluated jointly, as it can substantially alter the spatial classification of drought severity even for the same date. For instance, in Tunisia, the SPI-12 map for March 2024 (Fig. 5d) classifies the country as experiencing moderate drought, whereas the SPI-48 map (Fig. 5e) categorizes nearly the entire territory under extreme drought, consistent with widespread aridity across NA. This comparison underscores the necessity of analyzing multiple accumulation periods simultaneously to capture both short- and long-term drought dynamics. Also, integrating multiple data sources is likewise essential for a comprehensive understanding of the spatial and temporal variability of drought conditions across complex climatic regimes.

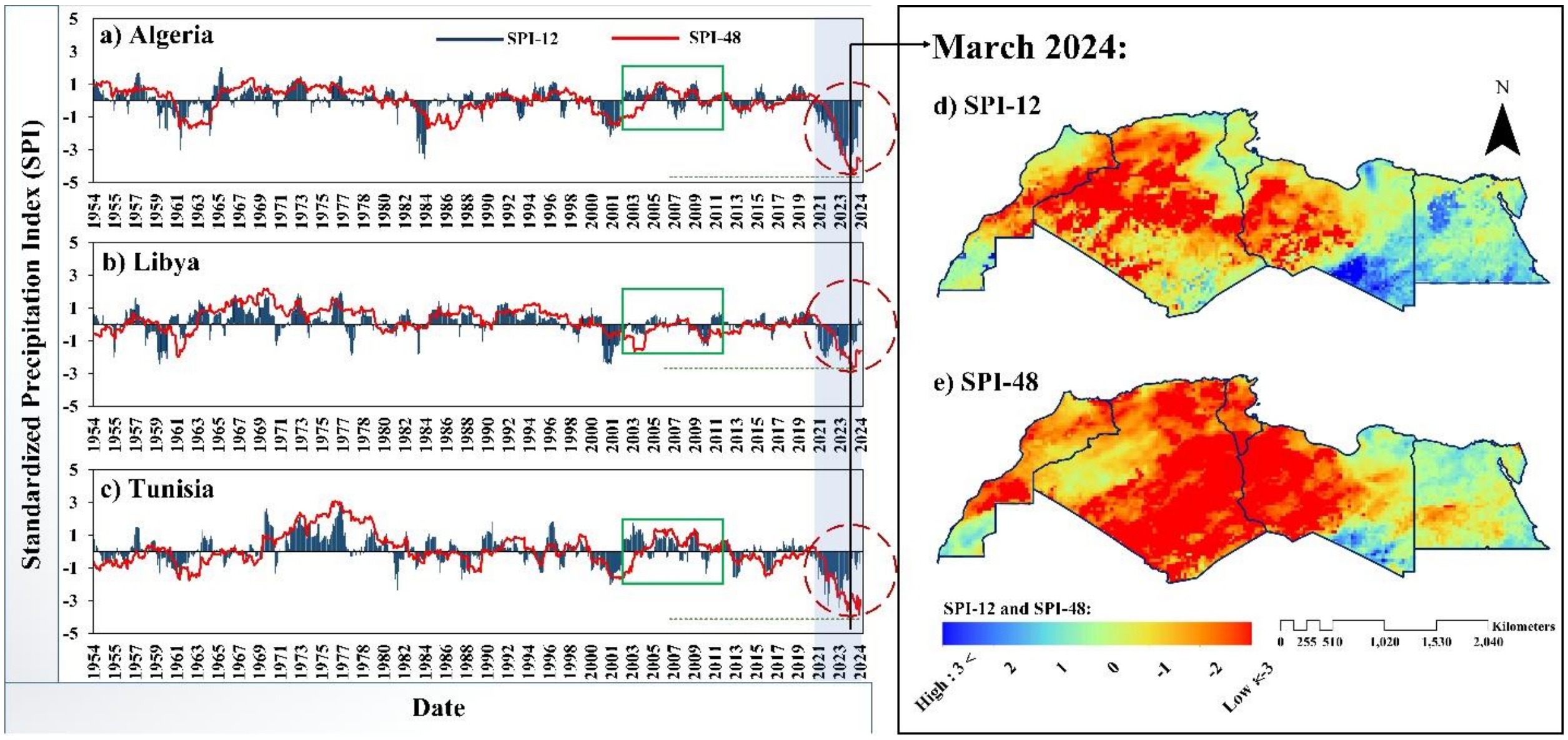

Fig. 5. Time-series variations of SPI-12 and SPI-48 based on ERA5 data at the national scale for Algeria, Libya, and Tunisia (a, b, c), and spatial distribution maps of both accumulation period (-12 and -48) across North Africa for March 2024 (d, e)."

3.6- Table 1 and Line 207: The authors should clarify what is meant by "temperature." Does this refer to mean, maximum, or minimum temperature? Providing this specification is essential for the accurate interpretation of the results.

Response: The authors thank the reviewer for this observation. We specified that "temperature" refers to monthly mean 2-m air temperature from ERA5-Land.

Edited manuscript (line 113):

"Mean surface air temperature (at 2-meter)"

3.7- Figure 2 and Line 210: The choice of colour scheme must be checked. It is unclear why significant changes are represented in red and non-significant changes in green. This convention may lead to misinterpretation, as red is typically associated with not significant trend.

Response: We have carefully reviewed the color legend and confirmed that all labeling is correct. Since the main objective of this figure is to highlight the significance and intensity of abrupt changes, the use of red to represent significant variations was intentional. This color configuration enhances visual contrast and enables readers to easily identify areas undergoing strong or critical transitions.

Edited manuscript (lines 271-272):

"In all maps depicting these three variables (Figure 2), the color legend is standardized so that red corresponds to the most pronounced transitions toward drier conditions."

As noted, we used the inverse scheme for temperature maps to maintain consistency with the variable's directionality. Therefore, we believe the current representation provides the clearest visual interpretation. Nonetheless, if the editorial team prefers an alternative color adjustment, we will be pleased to implement it in the next version.

3.8- Figure 2 (Slope colour scale): The slope colour scale appears inverted for temperature and evapotranspiration, which complicates visual interpretation. The authors should harmonise the colour scale across all variables.

Response: As explained above (Response 3.7), the slope color configuration was intentionally designed to simplify interpretation by ensuring that red consistently denotes regions of intensified hydroclimatic stress, whether caused by decreasing precipitation or increasing temperature and evapotranspiration. In this framework, red hues represent areas of warming and drying (positive slopes or high probabilities of change), while blue/green shades correspond to stability or wetting tendencies. This design allows for an intuitive interpretation: pixels appearing red across multiple variables (precipitation, temperature, evapotranspiration) clearly signify regions shifting toward drier and warmer conditions. We therefore prefer to retain the current legend for coherence and interpretive clarity. However, should the reviewer or editors consider harmonization of the color scales essential, we will readily make the adjustment in the final version, acknowledging that this may slightly reduce the visual distinctiveness of hydroclimatic contrasts.

3.9- Line 224: A clear negative trend in evapotranspiration (ET) is observed in southern Egypt. The authors, however, state that ET exhibits a positive trend. Please check this incongruity.

3.10- Line 225: In this case precipitation recorded main negative changes for the southern Egypt region.

Line 224-225: "Additionally, positive slope changes in evapotranspiration are observed in southern Egypt at critical breakpoints, particularly in areas where increasing precipitation trends have been detected."

Response 3.9 and 3.10: We appreciate the reviewer's thorough review and careful observation. After re-examining the maps, we confirmed that evapotranspiration (ET) trends in southern Egypt display a mixed "salt-and-pepper" pattern, where some pixels indicate positive slopes while others show negative ones. Therefore, we revised the corresponding text to clarify this spatial heterogeneity and to avoid overgeneralization.

Edited manuscript (lines 275-280):

"These fig.s 2 e,f, shown evapotranspiration in southern Egypt exhibits a heterogeneous pattern, with both positive and negative slope changes across adjacent pixels. Nevertheless, the number of

pixels displaying decreasing evapotranspiration coinciding with increasing precipitation appears greater, suggesting localized hydroclimatic recovery in some parts of the region. Comparison of the maps in Figures 2a and 2b reveals that no significant change points are observed in the precipitation time series across a broad spatial extent encompassing a large number of pixels within the studied countries."

3.11- Line 226: This sentence repeats information already provided a few lines earlier and should therefore be removed to avoid redundancy.

Response: We agree with the reviewer's observation. In accordance with this comment, the redundant sentence at line 226 has been removed to avoid repetition and improve the conciseness of the text. The authors would like to thank the reviewer for the detailed review of the manuscript.

3.12- Line 227: The content of this line is more appropriate for the Discussion section, as it involves interpretation rather than direct presentation of results. The authors are encouraged to relocate it accordingly.

Response:

We appreciate this valuable observation. The sentence in lines 227–229: "The rise in temperature, combined with increased evapotranspiration, has led to higher water demands for crops, indicating that more water resources are needed for agriculture compared to the past.", indeed contains interpretative content rather than direct results.

Considering the importance of interpreting results in relation to previous studies, we have restructured the manuscript to present the Results and Discussion sections in an integrated manner. This approach allows us to discuss the findings while they are introduced, thereby enhancing the logical flow and emphasizing how the observed trends contribute to understanding amplified drought linked to anthropogenic influences.

3.13- Line 242: The criteria used to select the eight representative stations are not clearly described. The authors should specify the selection base on geographic position, data completeness, climatic representativeness

Response: We appreciate the reviewer's observation. In fact, all available stations were used for the full analysis, while the eight stations shown in Figure 4 were selected as representative examples. These stations were chosen because they exhibited the most complete and continuous records with minimal missing data, and they provide balanced geographical coverage across all North African countries. To address this concern and enhance transparency, we have now clarified the selection criteria in the revised manuscript and included the full set of time-series plots for all

35 stations, corresponding to the red points shown in Figure 1, as supplementary material in addition to Figure 4.

Linke: 35_Stations_SPI-3-6-9-12-24-48: [https://figshare.com/s/57248f9d919bf1f46eab]

Edited manuscript (lines 243-244):

"', selected as a sample from all available stations (refer to the Supplementary File for additional details)."

3.14- Line 247: It's discussion

Response: We agree with the reviewer. In the revised version, the sections addressing meteorological and hydrological drought variations have been presented jointly under an integrated Results and Discussion structure. A dedicated subsection has been added and directly linked to the results to discuss the Indications of Anthropogenic Contributions to Drought Amplification, explaining in detail why the evidence suggests amplified drought linked to human influences.

3.15- Figure 4: It is recommended to include, within the figure, the name of each station and the corresponding country. This addition would significantly improve the clarity and contextual understanding of the results.

Response: Yes, we fully agree with the reviewer's suggestion, and this modification has been implemented.

Before:

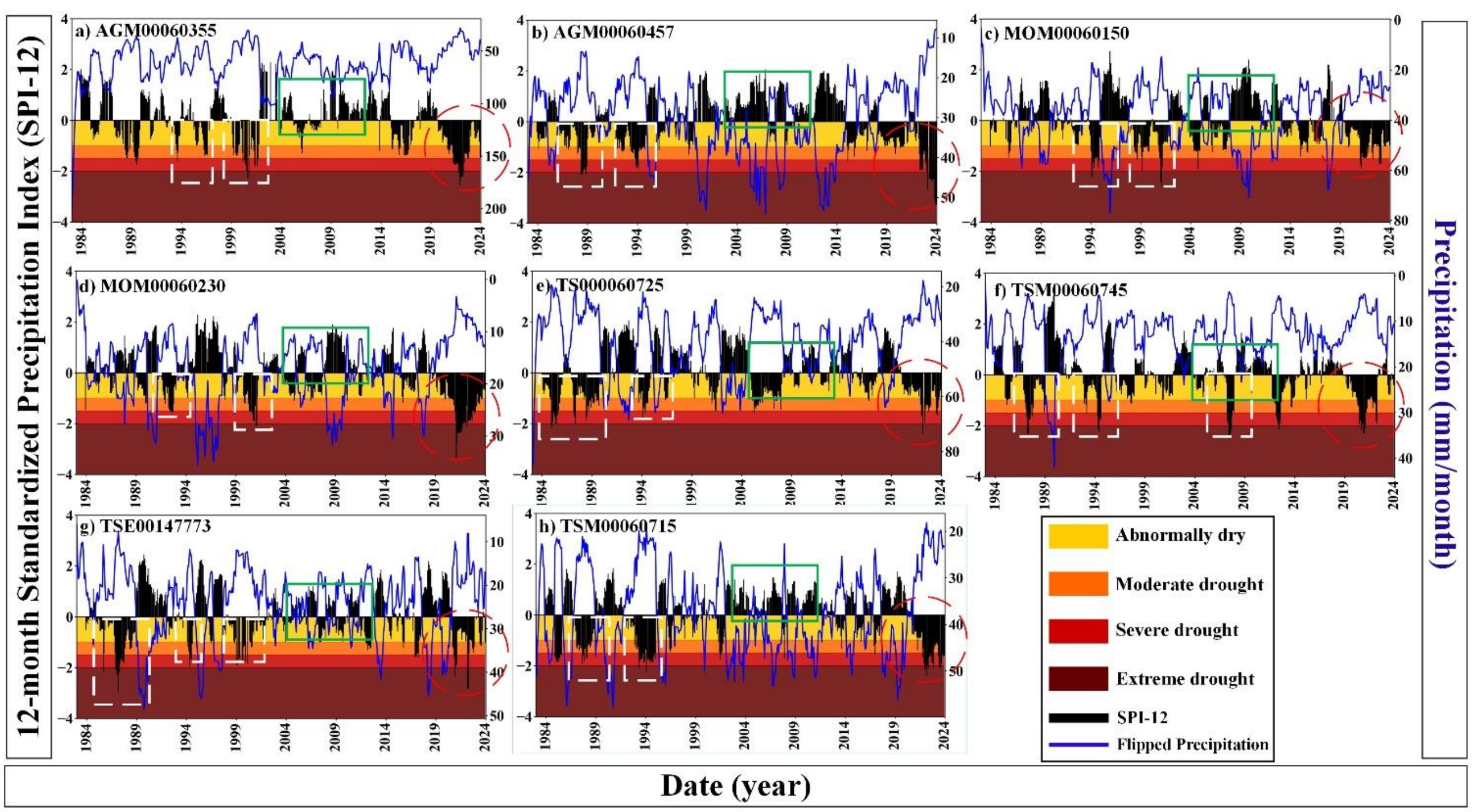

After:

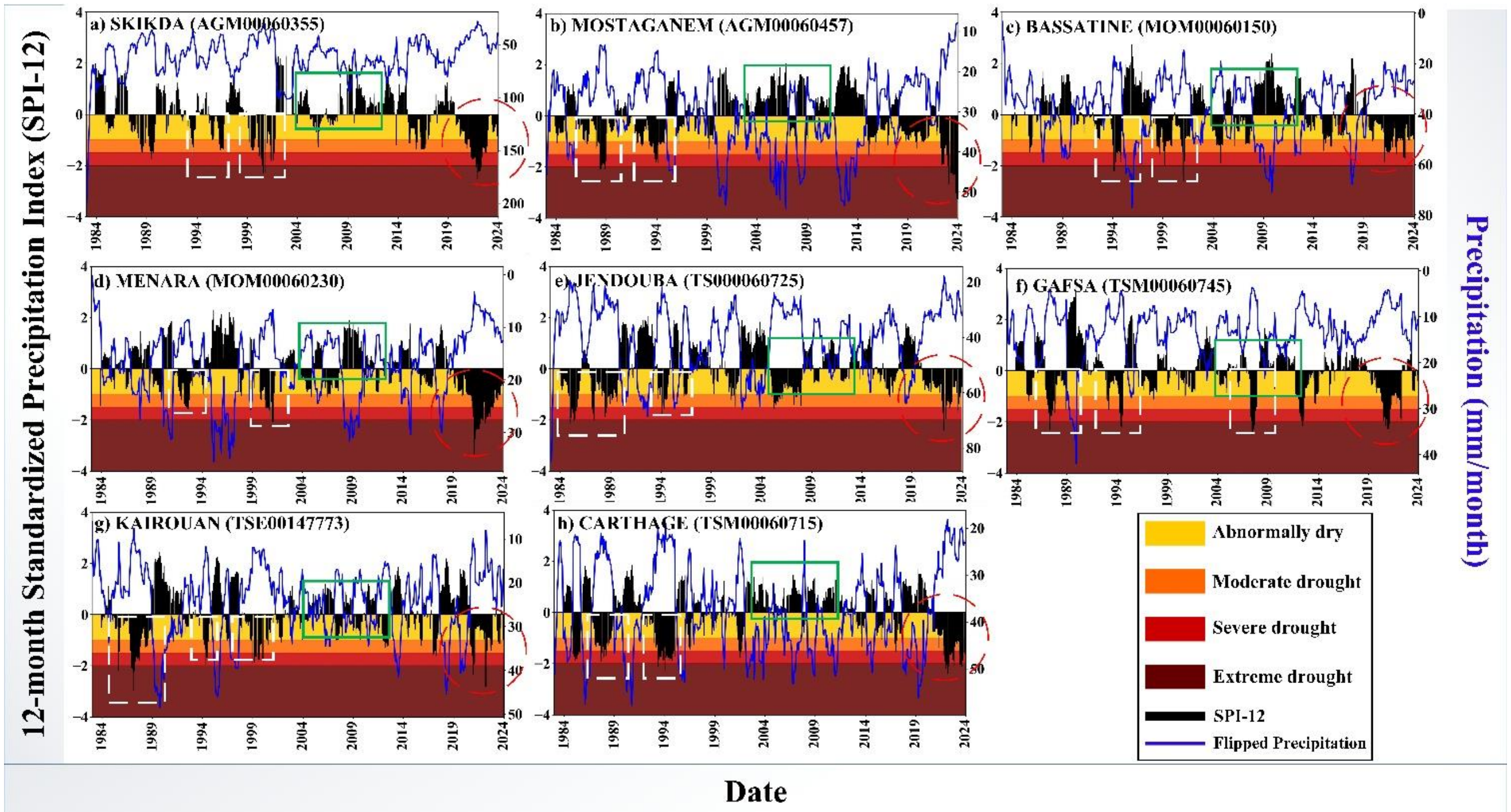

3.16- Line 255 Why the authors decided to average SPEI over the entire NA and create a single SPEI series for the entire study area? It is clear that there are different drought behaviours for each country. Due to the complexity of drought events it is important investigate on the temporal variability, but the spatial is important too. As the authors did for precipitation, temperature and ET form ERA5Land products

Response: Since our analysis began at the station level and interpretations were provided for each country individually before reaching regional conclusions, we retained the aggregated SPEI series to represent the overall regional behavior. However, following the reviewer's recommendation, we have also added drought index variations derived from the ERA5 dataset (as mentioned early at Response 3.5).

In addition, the detailed SPEI results have been made publicly available for interested readers.

Linke:SPEIbase_Data_and_Spatiotemporal_variations:[https://figshare.com/s/57248f9d919bf1f46eab]

3.17- Line 273: It is unclear how this conclusion was derived from the temporal series analysis. Did the authors achieved this result through comparison with the eight reference series? Further clarification of the methodology behind this statement is required. Therefore, I strongly emphasize the importance of analysing drought dynamics also at the spatial scale to capture the regional variability of the phenomenon.

Response:

lines 272-273: "Widespread extent: The drought spans a vast region, particularly affecting three countries, Tunisia, Algeria, and Morocco; in a synchronized pattern. This broad extent aligns with regional temperature and precipitation anomalies (see Fig. 2)"

Please note that this conclusion represents a synthesis of multiple analyses rather than an inference drawn solely from the eight representative stations. As indicated, this summary statement appears at the end of the Results section and is derived from the combined interpretation of all analyses, particularly Figures 4, 5, 6, and 8, to present a comprehensive and integrated understanding of regional drought evolution. The detailed workflow has been described in the Methods subsection "Spatiotemporal processing of multi-source datasets." In accordance with the reviewer's comment, we also added a new figure (Fig. 5 in the revised version) to strengthen the spatial interpretation.

In this study, spatiotemporal variations were analyzed simultaneously. For example, based on the SPEIbase dataset, the results for SPEI-12, SPEI-24, and SPEI-48 were jointly presented and analyzed in Figure 6 (formerly Fig. 5). Each point in this figure represents an area-weighted mean of all pixels located within Tunisia, Algeria, and Libya. Consequently, each time step in the figure reflects aggregated spatial information over a broad regional domain, summarizing multiple figures into a single comprehensive time-series representation. Moreover, 35 ground-based observational stations were analyzed individually to validate and complement these gridded results.

To further enhance transparency and reproducibility, the complete outputs from all analyses and corresponding datasets have been made available in the Supplementary Information.

Linke: 35_Stations_SPI-3-6-9-12-24-48:[https://figshare.com/s/57248f9d919bf1f46eab]

Linke: 131_Stations_decomposition-timeseries:[https://figshare.com/s/57248f9d919bf1f46eab]

Linke:SPEIbase_Data_and_Spatiotemporal_variations:[https://figshare.com/s/57248f9d919bf1f46eab]

3.18- Line 302: The content presented here describes methodological procedures and should be relocated to the Materials and Methods section for better structural coherence.

Response: lines 302-304: "Therefore, in this section, the analysis begins with a spatiotemporal evaluation of TWSA using satellite observations from GRACE. This is followed by a time series assessment of drought conditions using three key indices SFSM, RTZSM, and GWS, estimated at three different soil depths across each of the five countries."

We appreciate the reviewer's observation. Yes, we agree, and done.

3.19- Line 314: This a discussion

Response: We appreciate the reviewer's observation. Yes, we agree, given the revised structure of the manuscript, the Results and Discussion sections have been integrated.

3.20- Figure 6: Please confirm figure refers to the TWSA series. If so, this should be explicitly stated in the figure legend to avoid ambiguity.

Response: Yes, this clarification has been made. The figure caption has been revised to explicitly state the variable represented:

Edited manuscript (lines 409-411):

"Fig. 7. Spatiotemporal distribution of the slopes corresponding to the points of maximum probability of abrupt change in the monthly total water storage (TWS) time series, based on CSR and JPL GRACE datasets for the period 2002–2024."

3.21- Line 343: It's discussion

3.22- Discussion:All discussion-related statements currently included in the Results section should be relocated to the Discussion section to improve the structural clarity of the manuscript.

Response 3.21 and 3.22: As mentioned in Response 3.19, given the analytical nature of the results, we intentionally adopted an integrated Results and Discussion structure. This approach allows the interpretation of findings to be presented directly alongside the corresponding analyses, ensuring that key concepts and implications are clearly conveyed.

3.23- Moreover, the authors are encouraged to develop this section further by exploring in greater depth the relationships between meteorological drought and the climatological variables. Incorporating statistical analyses, such as Pearson's correlation test between drought indices and climatological variables.

Response: We appreciate the reviewer's suggestion. Although examining the relationships between meteorological drought and climatological variables can provide useful statistical insights for hydrological analysis, the authors are afraid that placing extensive emphasis on such correlations would divert attention from the main objectives of this study. The objective of the study is not to quantify the strength of these relationships, but rather to identify spatiotemporal drought patterns and their potential anthropogenic amplification.

3.24- The results related to Groundwater Storage (GWS) are particularly important, and the authors are encouraged to place greater emphasis on this aspect, as it represents a valuable contribution to the understanding of drought processes in the study area. What about bibliography on human water consumption?

Response: Thank you for this suggestion. It is indeed a valuable recommendation that we plan to pursue in future research. In the present study, our primary objective was to analyze the spatiotemporal behavior of hydro-meteorological variables and to highlight the major shifts detected in groundwater storage. While the current work focuses on how GWS has changed, understanding why it has changed requires further in-depth investigation. Future studies will therefore aim to integrate both bibliographic and quantitative data on human water consumption to more explicitly link groundwater depletion with anthropogenic drivers.

3.25- An important strength of the manuscript lies in the extensive effort devoted to the collection and processing of multiple datasets, each providing distinct and complementary types of information. This integrative approach is particularly valuable given the significant data gaps that 40haracterize the study area. The authors are encouraged to emphasis this aspect more explicitly in the manuscript, as it represents a novel and commendable contribution to regional drought assessment and climatological research.

Response: We sincerely appreciate the reviewer's insightful and positive comments highlighting one of the key strengths of our study. We fully agree that the integrative and multi-dataset approach represents an essential and novel contribution to regional drought and climatological research, particularly in data-scarce environments such as North Africa.

Accordingly, the Abstract, Introduction, and Conclusion sections have been revised to more explicitly emphasize this point, namely, that the present study integrates multiple datasets through a comparative framework to identify spatiotemporal distinctions and breakpoints among different hydro-meteorological variables. This integrative strategy not only compensates for the substantial data gaps across the region but also establishes a robust foundation for future drought-related investigations.

Edited manuscript:

Abstract (line 22): "a region characterized by pronounced data scarcity and observation gaps.".

Introduction (lines 76–77): "Where severe data scarcity and limited ground-based observations pose major challenges to conventional drought assessment[4]."

Conclusion (lines 484–485): "This region, characterized by severe data scarcity, observational gaps, and limitations in documenting human withdrawals,"

Additionally, we have made available a broad range of supplementary datasets to support reproducibility and to assist future studies facing similar data challenges.

Linke: datasets:[https://figshare.com/s/57248f9d919bf1f46eab]

3.26- Conclusion: The Conclusion section should be revised to reflect the new insights and interpretations arising from the expanded Discussion section.

Response:

We thank the reviewer for this valuable suggestion. The Conclusion section has been revised to integrate the new insights and interpretations emerging from the expanded Discussion. The revised version now provides a more coherent synthesis of the key findings and their broader implications for sustainable water resource management. It explicitly acknowledges existing uncertainties, highlights the role of anthropogenic drivers in amplifying hydrological drought, and outlines remaining research gaps that define future directions for continued investigation.

4) Reference

1 Zhao, K., Valle, D., Popescu, S., Zhang, X. & Mallick, B. Hyperspectral remote sensing of plant biochemistry using Bayesian model averaging with variable and band selection. *Remote Sensing of Environment* **132**, 102-119 (2013). https://doi.org/https://doi.org/10.1016/j.rse.2012.12.026

2 Zhao, K. *et al.* Detecting change-point, trend, and seasonality in satellite time series data to track abrupt changes and nonlinear dynamics: A Bayesian ensemble algorithm. *Remote Sensing of Environment* **232**, 111181 (2019). https://doi.org/https://doi.org/10.1016/j.rse.2019.04.034

3 Hu, T. *et al.* Mapping fine-scale human disturbances in a working landscape with Landsat time series on Google Earth Engine. *ISPRS Journal of Photogrammetry and Remote Sensing* **176**, 250-261 (2021). https://doi.org/https://doi.org/10.1016/j.isprsjprs.2021.04.008

4 Tanarhte, M., De Vries, A. J., Zittis, G. & Chfadi, T. Severe droughts in North Africa: A review of drivers, impacts and management. *Earth-Science Reviews* **250**, 104701 (2024). https://doi.org/https://doi.org/10.1016/j.earscirev.2024.104701

5 Alvarez, I., Diaz-Poso, A., Lorenzo, M. N. & Roye, D. Heat index historical trends and projections due to climate change in the Mediterranean basin based on CMIP6. *Atmospheric Research* **308**, 107512 (2024). https://doi.org/https://doi.org/10.1016/j.atmosres.2024.107512

6 Bazza, M., Kay, M. & Knutson, C. Drought characteristics and management in North Africa and the Near East. (2018).

7 Fragaszy, S. R. *et al.* Drought monitoring in the Middle East and North Africa (MENA) region: participatory engagement to inform early warning systems. *Bulletin of the American Meteorological Society* **101**, E1148-E1173 (2020).

8 Bhalla, S. *et al.* The role of interacting social and institutional norms in stressed groundwater systems. *Journal of Environmental Management* **356**, 120389 (2024). https://doi.org/https://doi.org/10.1016/j.jenvman.2024.120389

9 Valjarević, A., Šiljeg, A., Šiljeg, S., Vujović, F. & Sahay, A. GIS-based water stress analysis in North African drylands. *Journal of Arid Environments* **230**, 105427 (2025). https://doi.org/https://doi.org/10.1016/j.jaridenv.2025.105427

10 Prospero, J. M. & Nees, R. T. Impact of the North African drought and El Niño on mineral dust in the Barbados trade winds. *Nature* **320**, 735-738 (1986). https://doi.org/10.1038/320735a0

11 Applequist, S., Durre, I. & Vose, R. The Global Historical Climatology Network Monthly Precipitation Dataset, Version 4. *Scientific Data* **11**, 633 (2024). https://doi.org/10.1038/s41597-024-03457-z

Rebuttal Letter-2 for

Spatiotemporal hydrometeorological drought variations in North Africa: indications of anthropogenic contributions to amplify drought

Dear Editor and Reviewers,

We would like to express our deepest gratitude for your thorough review and insightful comments on our manuscript entitled "*Spatiotemporal hydrometeorological drought variations in North Africa: indications of anthropogenic contributions to amplify drought*" in ID: COMMSENV-25-3877A. We thank the Editor and all three reviewers for their careful evaluation of our manuscript and for the constructive comments that have substantially improved its scientific rigor, clarity, and transparency.

In the revised manuscript, we have added a new dedicated subsection entitled "Uncertainties and Limitations." This section explicitly addresses dataset-specific reference periods, the limitations associated with pixel-wise changepoint detection over large spatial domains, and the interpretative boundaries related to linking drought amplification to anthropogenic influences. In addition, we have provided an open-access link to the BEAST code to facilitate closer examination and reproducibility of the methodological framework.

We appreciate the opportunity to revise our manuscript in light of the reviewers' valuable feedback and hope that the revised version is now suitable for further consideration.

Sincerely,

The Authors

Reviewers' Responses to Questions (2):

1) Reviewer #1:

I appreciate the authors' efforts to revise the manuscript and address many of the concerns raised in the first round. The revised version shows improvement in structure and clarity; however, several key issues remain insufficiently resolved, particularly regarding data consistency, methodological transparency, and the strength of attribution claims.

Response: We thank the reviewer for the careful re-evaluation of the revised manuscript and for acknowledging the improvements in its structure and clarity. We recognize that such a thorough review is time-consuming, and we appreciate the time and effort you devoted to improving this manuscript. The reviewer's constructive comments and suggestions have been invaluable in improving the quality of the manuscript. Below, we address each of the remaining concerns in detail.

Remaining Major Concerns:

1.1- Data Consistency and Baseline Definition: There are still inconsistencies regarding the temporal coverage and baseline periods used for ERA5/ERA5-Land and related datasets. Different sections and figures appear to rely on different reference periods without clear justification. This lack of consistency makes it difficult to fully assess the robustness of trends, percentiles, and drought classifications.

Response: We thank the reviewer for raising this important point again. ERA5-based drought indices (SPI) were calculated at multiple accumulation scales (12 and 48 months) using the 1991–2020 reference period, which corresponds to the longest continuous overlap between reanalysis and satellite-based products.

We fully agree that the absence of a single unified baseline for directly comparing absolute values across multiple indices over a fixed study domain may introduce uncertainty in aggregated interpretations. However, it is important to clarify that the primary objective of this study is not to intercompare absolute magnitudes across different indices, but rather to analyze the temporal behavior and trend characteristics of each index across different countries.

Accordingly, within each figure and analytical subsection, comparisons are performed consistently across countries using a fixed reference period specific to that dataset. This section-by-section approach ensures internal consistency and allows a robust assessment of trend behavior, percentiles, and drought classifications at the national scale. Under this framework, the central finding, that Tunisia exhibits a distinctly different temporal behavior compared to neighboring countries, remains robust across datasets and indices.

This strategy was deliberately adopted to minimize methodological bias while maximizing the use of all available data. In each analytical section result, a clearly defined baseline is applied, and for synthesis purposes, the common period (2002–2024) can be regarded as a broad comparative

window across sections. However, imposing a single baseline across all indices and datasets would, in our view, be methodologically inappropriate, as it would (i) substantially restrict the temporal coverage to a short common period, (ii) discard a large portion of valuable information, (iii) reduce the statistical robustness of trend detection due to shortened records, and (iv) introduce additional uncertainties arising from intrinsic differences among data sources.

By analyzing each index over its full available record and comparing its behavior spatially across countries, we aimed to (i) reduce source-specific measurement and retrieval uncertainties, (ii) exploit the full informational content of each dataset, and (iii) focus on relative temporal dynamics rather than absolute values. Notably, despite differences in baselines and data sources, the indices consistently reveal coherent patterns of change, as discussed in detail in the manuscript.

We acknowledge that this approach requires transparent communication of its limitations. Therefore, a dedicated subsection on "uncertainties and limitations" has been added to explicitly address baseline differences and their implications for interpretation.

Revised manuscript: "In this study, each dataset and drought index was analyzed independently using its native temporal coverage and a dataset-specific reference period at either the national or regional scale. This methodological choice was deliberately adopted to avoid discarding valuable long-term information that would otherwise be lost if all analyses were constrained to a single, shorter common period across heterogeneous data sources. As a result, the temporal behavior of each country's time series was examined over the full available record of the corresponding dataset, enabling robust identification of country-specific temporal patterns, breakpoints, and inter-country similarities and differences without relying on direct comparisons of absolute index magnitudes. While this approach minimizes information loss and enhances the detection of long-term variability and non-stationarity, it also introduces uncertainty related to differences in dataset-specific reference periods, which is acknowledged as an inherent limitation of multi-source drought analyses.

Importantly, this strategy does not compromise the robustness of the main conclusions. Despite differences in native temporal coverage, a sufficiently long common overlapping period spanning 2002–2024 exists across all major datasets employed in this study. This shared window encompasses the most critical hydroclimatic features discussed, including the pronounced post-2010 shifts and the unprecedented emerging drought observed between 2021 and 2024, and provides an independent basis for cross-validation of temporal coherence across datasets. Moreover, interpretations are based primarily on relative temporal dynamics and trend behavior rather than on absolute magnitudes, thereby reducing sensitivity to source-specific biases and measurement uncertainties."

1.2- Use of Observational (GHCN) Data: While GHCN stations are mentioned, their exact role remains unclear. It is still not explicitly stated whether station data are used for validation, independent trend analysis, or illustrative purposes only. Given the importance of observational support, this point requires clearer explanation.

Response: We acknowledge the reviewer's concern and note that this issue was addressed in Sections 2.5 (Spatiotemporal processing of multi-source datasets) and 2.1 (Data: Observation, Global Datasets and Remote Sensing) of the revised manuscript.

Specifically, Section 2.5 states:

"Among these, 35 stations meeting four rigorous criteria, (1) the longest continuous observation records, (2) minimal data gaps, (3) stable instrumentation and consistent measurement protocols, and (4) uninterrupted reporting up to the present, were classified as best-quality stations. The SPI was computed for all selected stations over 1984–2024, and each point-based series was evaluated independently (Supplementary Files)."

Section 2.1 clarifies also the role of the observational data:

"The GHCN observations were used both to validate the SPI-based drought indices and to perform independent trend analyses using in situ series."

However, the authors agree that this is an important point, and it needs to be presented clearly. In response to the reviewer's request for greater clarity, this point has now been further emphasized in Section 2.1 to explicitly state that station data were used for independent trend analysis of the SPI drought index, rather than for illustrative purposes only.

Revised manuscript: "GHCN station data were used to compute the SPI and to conduct independent trend analyses of drought variability based on in situ precipitation series."

1.3- Changepoint Detection and Statistical Interpretation: The BEAST changepoint analysis is better described, but key implementation details (e.g. priors, convergence checks, interpretation of spatially extensive changepoints) remain limited. In addition, the manuscript does not sufficiently acknowledge the implications of applying pixel-wise changepoint detection over large spatial domains.

Response: We thank the reviewer for this important comment. Following the recommendation of another reviewer, we previously condensed the methodological description in the main text and moved detailed implementation settings to the Supplementary Information. In response to the present comment, we confirm that the following information has now been explicitly documented in the Supplementary Materials.

"2.1 Bayesian time-series decomposition using BEAST

2.1.1 MCMC settings and convergence:

The revised text now specifies that four parallel RJ-MCMC chains were run for each pixel, each consisting of 60,000 iterations, with the first 20,000 discarded as burn-in and a thinning factor of 10. This yielded 16,000 posterior draws used for inference. Random seeds were recorded for reproducibility. Convergence was evaluated through trace plots, the Gelman–Rubin statistic ($\hat{R} \leq 1.05$), and effective sample sizes (ESS > 500). These settings are consistent with recommendations from Zhao, et al. [1,]Zhao, et al. [2] and Hu, et al. [3].

2.1.2 Priors and hyperparameters:

We now include the explicit prior forms: (1) a discrete uniform prior on the number of changepoints (0–5 per layer), (2) conditional uniform priors on changepoint locations, (3) Gaussian priors with broad variances for the trend and seasonal coefficients, and (4) a weak inverse-gamma prior for the noise variance.

1.1.1 Computation of changepoint probabilities:

The posterior inclusion probability (PIP) for each time step is defined as the proportion of posterior samples in which that time is identified as a changepoint. This metric is directly derived from the RJ-MCMC sampling process, following Zhao, et al. [2]."

Please note that we are not the developers of the BEAST model, and all implementations in this study strictly follow the recommendations provided by the original model developers. For instance, as you are aware, convergence assessment is one of the most challenging aspects of MCMC-based inference, and monitoring convergence using simple diagnostic statistics is often difficult. As a practical remedy, the BEAST developers recommend running the hybrid MCMC sampler for a sufficiently long duration to safeguard against instability. Accordingly, the length of each individual chain in our experiments was set to 60,000 iterations, with the first 10,000 samples discarded as burn-in. We further thinned the chains by retaining only every fifth sample. In addition, a total of five such chains were run in parallel, thinned, and finally merged into a single posterior chain. During the iterative process, all chains were initialized from the null model (i.e., no covariates selected), although alternative initializations are also permissible within the BEAST algorithm.

We intentionally refrained from providing additional low-level implementation details in the main manuscript for two reasons. First, extensive technical elaboration could give the impression that we developed or modified the BEAST algorithm, which is not the case. Second, an overly detailed methodological exposition would detract from the primary scientific objectives of the study. Nevertheless, the full methodological setup is clearly documented, and all relevant references are provided for readers interested in further technical details.

In addition, we have explicitly included the public access link to the BEAST code in the manuscript, stating that:

Revised manuscript: "The Bayesian Estimator of Abrupt change, Seasonal change, and Trend (BEAST) algorithm is publicly available at [https://github.com/beast-dev/beast-mcmc] [44,45].".

Furthermore, the authors thank the reviewer for their comment that "the manuscript does not sufficiently acknowledge the implications of applying pixel-wise changepoint detection over large spatial domains," this issue is now explicitly addressed in the "Uncertainty and Limitations" section. Specifically, we acknowledge that:

Revised manuscript: "Furthermore, the analyses combined large-scale spatial averaging with pixel-wise statistical evaluations, each introducing its own analytical constraints. In particular, pixel-level changepoint detection across large spatial domains may produce statistical uncertainties and spatial artifacts, especially in heterogeneous hydroclimatic regions. These potential limitations were anticipated and are explicitly acknowledged within the uncertainty framework of this study."

The authors reiterate their thanks to the reviewer for their judicious comment and hope they find the way we addressed it acceptable. We tried to make sure the information is presented clearly, while avoiding making the manuscript overly long, and managing the incompatible comments provided by different reviewers.

1.4- Attribution to Anthropogenic Influences: The revised text still makes relatively strong claims linking drought amplification to anthropogenic drivers, including groundwater abstraction. While the patterns presented are suggestive, direct attribution remains unsupported by independent socio-hydrological or land-use data. Given that anthropogenic influence is highlighted in the title, these conclusions should be more cautiously framed or further substantiated.

Response: This is indeed a very important point and the authors thank the reviewer for highlighting it. We have addressed this issue in detail and, in response to related concerns, have already revised the manuscript title to adopt a more cautious framing. Following the reviewer's comment, we have carefully re-examined the entire manuscript and further refined the language wherever possible to ensure that interpretations regarding anthropogenic influences are expressed with appropriate caution.

In addition, we have introduced a dedicated subsection entitled "Uncertainty and Limitations", in which we explicitly acknowledge the assumptions, constraints, and uncertainties associated with our interpretations. This section clearly states that the observed patterns are indicative rather than deterministically attributable, and that direct attribution is limited by the availability of independent socio-hydrological and land-use data.

Revised manuscript: "The spatiotemporal hydrometeorological drought results reveal pronounced changes after 2010, suggesting potential links between drought intensification and anthropogenic drivers such as groundwater abstraction. However, despite the focus on anthropogenic influences, this study remains strictly confined to hydrological and hydroclimatic evidence. Therefore, these interpretations should be approached with caution. The limitations of the present research underscore the need for future studies to adopt integrated socio-hydrological approaches that investigate groundwater governance, land-use change, and water consumption, particularly

agricultural water use, in Tunisia, where the most pronounced post-2010 anomalies were observed."

We emphasize that the current version reflects our most balanced and conservative interpretation of the available evidence. However, should the reviewer wish to see specific sentences, phrases, or sections further modified or removed, we would be grateful for explicit guidance and will implement such changes accordingly. We are also open to further revising the manuscript title, if deemed necessary by the reviewer or editor.

At this stage, we believe the revised manuscript represents the most appropriate balance between scientific rigor and interpretative caution, and that additional reductions may risk removing key contextual insights derived from the analyses. Again, the authors thank the reviewer for having pointed out this important point.

1.5- Regional vs Local Interpretation: Some conclusions extrapolate from 7ocalized signals to regional-scale drying without sufficient justification. Statements regarding widespread or sustained regional drying should be more carefully aligned with the spatial extent of statistically robust results.

Response: This comment most likely refers to Section 3.1.2 (Meteorological drought index results). In this section, our analysis is based exclusively on the SPEIbase dataset, and no extrapolation beyond the spatially aggregated evidence provided by this dataset was intended. To further enhance transparency, we have made the raw SPEIbase data publicly available and provided the corresponding access link in the manuscript and Supplementary Information.

We acknowledge the reviewer's concern regarding the alignment between localized signals and regional-scale interpretations. We emphasize that statements referring to regional or sustained drying are derived from spatially aggregated SPEI analyses rather than from isolated local signals. Nevertheless, should the reviewer consider that any specific sentence, phrasing, or interpretation overstates the spatial extent or robustness of the results, we would be grateful for explicit guidance and will readily revise or remove the relevant text accordingly.

Nevertheless, in this regard, we have revised the caption of Figure 6 and added the corresponding clarifications to the Uncertainties and Limitations section.

Revised manuscript:

"Fig. 6. Average SPEIbase time series over the North Africa for 3-, 12-, 24-, and 48-month timescales (1954–2024)"

and

"An additional limitation concerns the interpretation of localized versus regional-scale drought signals. In Section 3.1.2, meteorological drought variability is analyzed exclusively using the SPEIbase dataset, and all conclusions referring to widespread or sustained drying are derived from

spatially aggregated SPEI fields rather than from individual stations or localized anomalies. While local-scale variability is intrinsic to drought processes across North Africa, the regional interpretations presented here are based on statistically coherent patterns observed at aggregated spatial scales, thereby reducing the risk of extrapolating isolated signals to the regional domain. Nevertheless, spatial aggregation may partially obscure subregional heterogeneity, and caution is therefore warranted when interpreting regional-scale summaries in areas characterized by strong climatic and physiographic gradients. To enhance transparency and enable independent assessment of spatial variability, the raw SPEIbase data used in this analysis have been made publicly available (see Supplementary Files)."

1.6- Minor Issues: Some statistical outputs (e.g. p-values reported as 0.0000, very large Z-values) remain difficult to interpret and should be presented more carefully.

Response: We thank the reviewer for pointing out this issue. After re-examining the statistical outputs, we confirm that the reported values are correct. Nevertheless, to avoid potential misinterpretation and to improve clarity, we have decided to remove the p-value and trend columns from the table in question. This revision ensures that the presentation of results remains clear, robust, and consistent with best practices for statistical reporting.

1.7- Minor language and consistency issues persist and would benefit from a final editorial pass.

Response: In response to this comment, the manuscript has been carefully reread by a native English speaking scientist, and thoroughly edited to improve language quality, clarity, and internal consistency. If the reviewer identifies any specific remaining issues or passages requiring further revision, we would be grateful for their guidance and will address them promptly.

1.8- Overall: The manuscript addresses an important topic and has improved since the first review. However, key methodological clarifications and a more cautious interpretation, especially regarding anthropogenic attribution, are still required. Addressing these points would substantially strengthen the credibility and impact of the study.

Response: We sincerely thank the reviewer for the time and effort devoted to evaluating this manuscript, for the positive assessment of its relevance, and for the constructive identification of remaining shortcomings. Regarding the methodology, we have provided all essential details required for reproducibility and robustness, while deliberately avoiding excessive technical expansion that could give the misleading impression that the proposed methods were developed by the authors or distract readers from the primary scientific objectives of the study. Nevertheless, if the reviewer considers that any specific methodological aspect requires further clarification, we would be pleased to provide the additional details.

With respect to anthropogenic attribution, we have adopted a deliberately cautious and conservative interpretation throughout the revised manuscript. Our focus has been strictly on statistically supported hydroclimatic evidence, avoiding speculative or overstated claims and refraining from socio-political interpretations that fall beyond the scope of the present analysis. We agree that the human dimensions of drought amplification constitute an important and promising direction for future research, and this perspective has been explicitly acknowledged in the revised discussion.

Once again, we are grateful to the reviewer for their careful evaluation and valuable suggestions, which have helped to strengthen the scientific rigor and clarity of this work.

2) Reviewer #2:

Thank You for all your comments, the modified manuscript and comments seems satisfactory.

Response: We sincerely thank the reviewer for their positive assessment and for the time and valuable comments that contributed to improving the quality, clarity, and rigor of this manuscript. We truly appreciate their careful evaluation and wish them all the best.

3) Reviewer #3:

The authors have incorporated the vast majority of the suggestions made by all reviewers in a satisfactory way, which has significantly strengthened the paper. The structure is clearer, the methodological explanations are more transparent. Overall, the manuscript reads much more cohesively and makes a stronger contribution to the field.

Response: We are very grateful for your positive feedback. We appreciate the time you devoted to reviewing our manuscript and the detailed and constructive comments you provided, which significantly contributed to improving both the scientific content and editorial quality of the article.

I have only minor revisions to suggest:

3.1.1- Figure1 Please add the toponyms mentioned in the manuscript (e.g., Nile, Medjerda, Moulouya) are not clearly located on the map, which may confuse readers unfamiliar with the area.

Response: We thank the reviewer for this careful and helpful observation. The requested changes have been implemented, and Figure 1 has been revised accordingly to clearly indicate the rivers mentioned in the manuscript (e.g., the Nile, Medjerda, and Moulouya).

This is a new figure:

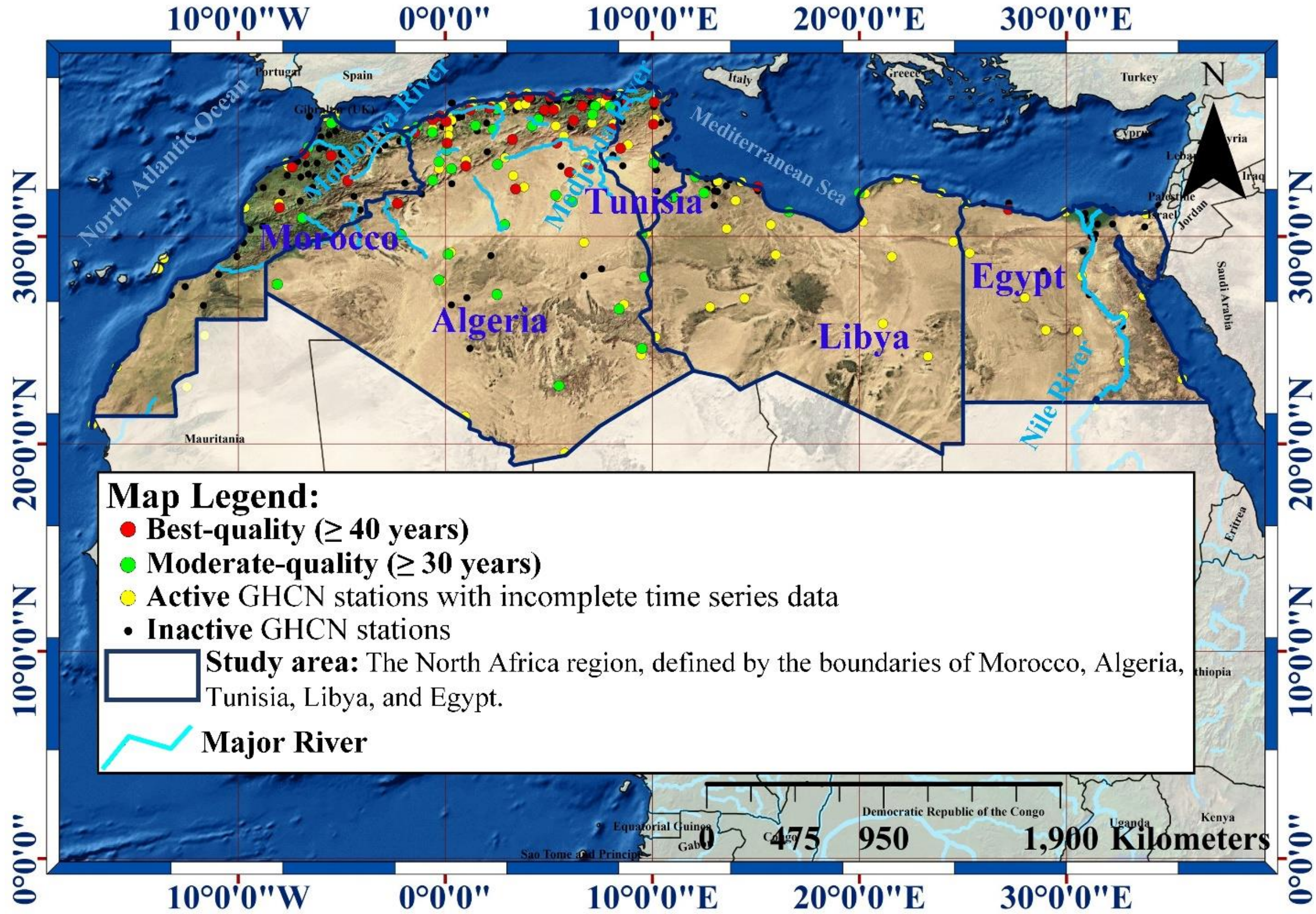

Replace with last figuer:

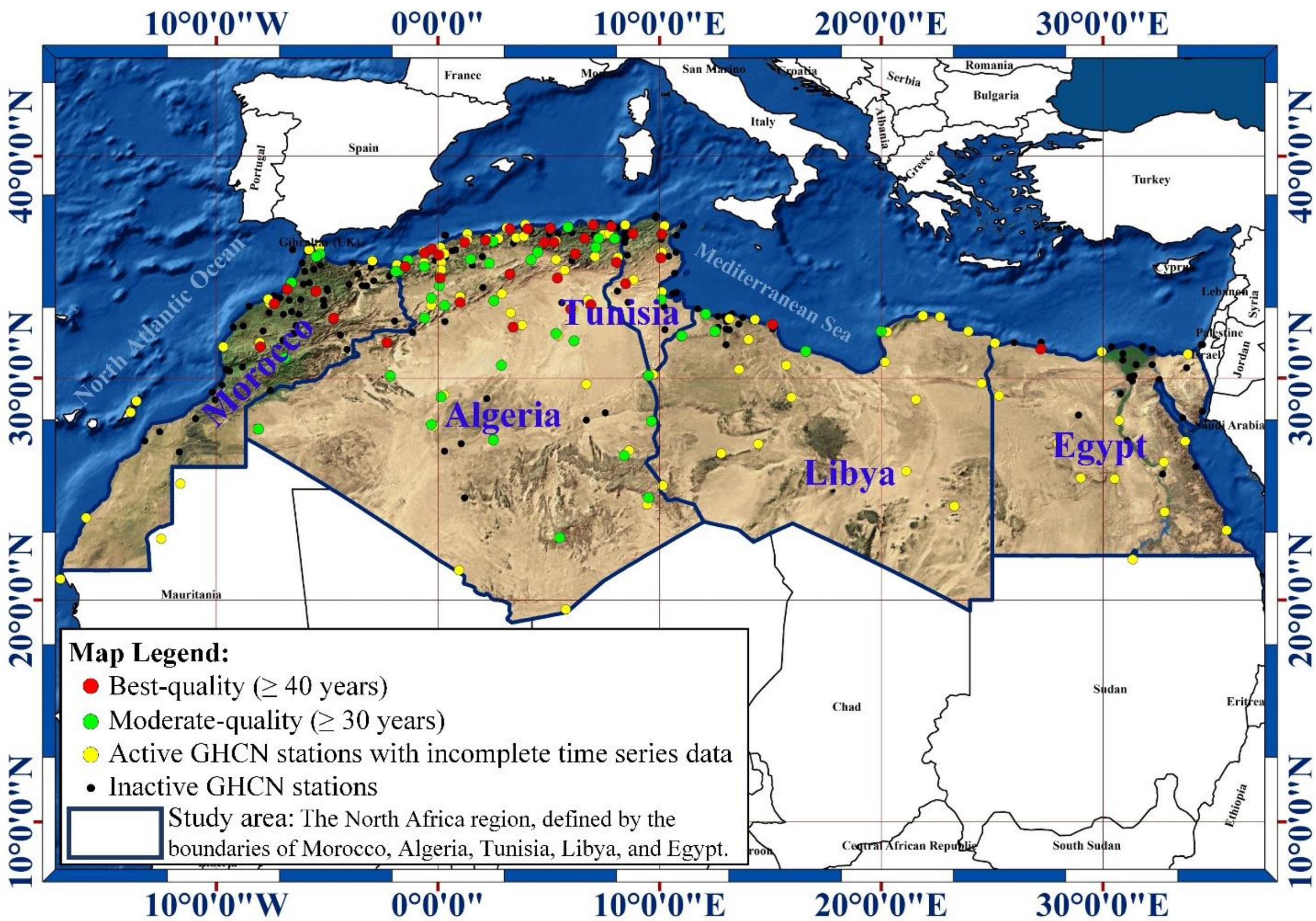

3.1.2- "2.5. Spatiotemporal processing of multi-source datasets the sentence "minimal data gaps" which percentage of missing data did the authors considered? Add reference

Response: We thank the reviewer for this important clarification request. In this study, stations were considered to have minimal data gaps if missing data did not exceed 30% of the total record length over the period 1994–2024, or if there were no continuous gaps longer than five consecutive years. These criteria were applied to ensure temporal continuity and robustness in trend and drought-index analyses. This definition has now been explicitly stated in Section 2.5, and an appropriate reference has been added to support the adopted threshold.

"minimal data gaps, if missing data did not exceed 30% of the total record length over the period 1994–2024, or if there were no continuous gaps longer than five consecutive years [4]."

4) Reference

1 Zhao, K., Valle, D., Popescu, S., Zhang, X. & Mallick, B. Hyperspectral remote sensing of plant biochemistry using Bayesian model averaging with variable and band selection.

Remote Sensing of Environment **132**, 102-119 (2013). https://doi.org/https://doi.org/10.1016/j.rse.2012.12.026

2 Zhao, K. *et al.* Detecting change-point, trend, and seasonality in satellite time series data to track abrupt changes and nonlinear dynamics: A Bayesian ensemble algorithm. *Remote Sensing of Environment* **232**, 111181 (2019). https://doi.org/https://doi.org/10.1016/j.rse.2019.04.034

3 Hu, T. *et al.* Mapping fine-scale human disturbances in a working landscape with Landsat time series on Google Earth Engine. *ISPRS Journal of Photogrammetry and Remote Sensing* **176**, 250-261 (2021). https://doi.org/https://doi.org/10.1016/j.isprsjprs.2021.04.008

4 Ben Aissia, M.-A., Chebana, F. & Ouarda, T. B. M. J. Multivariate missing data in hydrology – Review and applications. *Advances in Water Resources* **110**, 299-309 (2017). https://doi.org/https://doi.org/10.1016/j.advwatres.2017.10.002

Communication Earth & Environment

Rebuttal Letter-3 for

Spatiotemporal hydrometeorological drought variations in North Africa: indications of anthropogenic contributions to amplify drought

Reviewers' Responses to Questions (2):

1) Reviewer #1:

Thank you for revising the manuscript and addressing several of the earlier comments; however, there remain a number of important methodological, statistical, and interpretative issues that need to be resolved before the conclusions can be considered fully supported.

1.1- Inconsistent analysis periods and baselines. Key datasets (ERA5-Land, SPI, SPEI) are analysed over different and sometimes inconsistently stated periods (e.g., 1980–2024 vs 1981–2024), without a clearly defined, uniformly applied reference baseline, complicating trend and breakpoint interpretation.

Response: We thank the reviewer for highlighting this issue. The typographical inconsistency noted in line 272 (e.g., 1980–2024 vs. 1981–2024) has been corrected in the revised manuscript. In addition, all analysis periods for each dataset are now explicitly summarized in Table 1 in the Data section to ensure clarity and consistency.

1.2- Although many GHCN stations are available, it remains unclear which results are directly supported by station-based analyses versus gridded products, and whether station data provide validation or independent evidence for the main conclusions.

Response: We thank the reviewer for this clarification. In the revised manuscript, we now explicitly distinguish between results derived from station-based analyses and those based on gridded or reanalysis products.

GHCN station data are used to (i) compute SPI directly from in situ precipitation records and (ii) perform independent trend analyses at station locations. These station-based analyses provide independent observational evidence of drought variability and are presented separately from gridded-product results. They are not used for calibration, bias correction, or spatial interpolation of reanalysis datasets.

To ensure full transparency, the manuscript now states:

"Due to the limited spatial coverage and uneven distribution of GHCN stations across North Africa, no spatial interpolation of the station time series was performed. Instead, all analyses were conducted on a point-to-grid basis. GHCN station data were used to compute the SPI and to perform independent trend analyses of drought variability based on in situ precipitation records.

Comprehensive plots showing the trend, seasonal, and residual components for all 131 active stations (identified by station name), as well as SPI time series for 35 selected stations (indicated by red points in Fig. 1), are provided in the Supplementary Files."

1.3- No formal comparison (e.g., bias, correlation, trend or changepoint agreement) is provided between station observations and reanalysis, limiting confidence in cross-dataset consistency.

Response: We thank the reviewer for this comment. However, in the context of North Africa, an independent formal statistical comparison between GHCN station data and ERA5-based reanalysis products would not be methodologically appropriate.

ERA5 products are partially constrained by assimilated ground-based observations, including GHCN station data. As a result, station-based SPI series and ERA5-derived SPI are not statistically independent. Performing formal metrics such as bias, correlation, or changepoint agreement between these datasets would therefore not provide a meaningful assessment of cross-dataset consistency, as the two data sources are inherently dependent.

This dependence is explicitly acknowledged in the manuscript. As stated:

"Because ERA5 products are partly constrained by GHCN ground-based observations, their temporal behavior closely aligns with the station-based SPI results (Fig. 4 and Supplementary Material)."

Thus, rather than treating station data as an external validation dataset, we use them as independent point-based analyses that complement the spatially aggregated reanalysis results. The strong visual agreement between Figures 4 and 5 reflects this shared observational foundation.

1.4- The manuscript lacks clear operational descriptions of regridding, temporal aggregation, masking, and index computation across datasets with different resolutions and coverage, weakening reproducibility.

Response: We thank the reviewer for this comment. In the revised manuscript, we have expanded Section 2.1 in Supplementary document to provide a clear operational description of all preprocessing and harmonization steps applied across datasets with different spatial resolutions and temporal coverage.

"2.1 Operational description

All datasets were harmonized prior to analysis to ensure spatial and temporal comparability. Spatial regridding was performed using bilinear interpolation to a common 0.25° latitude–longitude grid. For datasets with coarser native resolution, interpolation was applied conservatively to preserve large-scale variability without introducing artificial fine-scale structure.

Temporal aggregation was conducted at the monthly scale. Daily datasets (e.g., ERA5-Land) were first aggregated to monthly means (for temperature and evapotranspiration) or monthly totals (for precipitation).

A consistent land mask was applied across all datasets to ensure spatial comparability within the North Africa domain. Missing values were excluded prior to statistical analysis.

The Standardized Precipitation Index (SPI) was computed using a gamma distribution fitted to monthly precipitation totals, following standard procedures. Accumulation periods of 12 and 48 months were selected to capture interannual and multi-year drought variability.

Because the GWS, RZSM, and SSM time series are weekly and may contain both seasonal cycles and temporal persistence, trend estimation was performed after explicitly accounting for seasonality and serial correlation. First, the seasonal component was removed using weekly climatology. For each variable and region, the long-term mean for each week of the year was calculated across the full study period and subtracted from the corresponding weekly observations. This produced deseasonalized anomaly series and prevented the regular intra-annual cycle from being interpreted as a long-term trend. Second, serial correlation was assessed using the autocorrelation function of the deseasonalized and detrended residual series for lags from 1 to 52 weeks. Autocorrelation coefficients exceeding $\pm 1.96/\sqrt{N}$ were considered statistically significant at the approximate 95% confidence level. Where significant lag-1 autocorrelation was detected, a trend-free pre-whitening procedure was applied before estimating the final trend magnitude. Sen's slope was then calculated from the deseasonalized and serial-correlation-adjusted series"

1.5- Essential implementation details (priors, MCMC settings, convergence checks, and treatment of multiple testing in pixel-wise analyses) are missing, making the robustness of detected changepoints difficult to assess.

Response: We thank the reviewer for this important comment. The essential implementation details of the BEAST changepoint analysis have now been explicitly provided in the Supplementary document to ensure full transparency and reproducibility:

"In a Bayesian framework, all unknown quantities are treated as random variables, including the model structure M, regression coefficients $\boldsymbol{\beta}_M$, and noise variance σ^2. For a given time, series $\mathcal{D} = \{t_i, y_i\}_{i=1,\dots,n}$, the objective is not limited to point estimation but extends to the full posterior distribution $p(\boldsymbol{\beta}_M, \sigma^2, M \mid \mathcal{D})$. According to Bayes' theorem, this posterior is proportional to the product of the likelihood and prior distributions:

$$p(\beta_M, \sigma^2, M \mid D) \propto p(D \mid \beta_M, \sigma^2, M)\pi(\beta_M, \sigma^2, M) \quad (1)$$

The observation model is expressed using the general linear representation:

$$y = X_M \beta_M + \varepsilon \quad (2)$$

where $\mathbf{y} = (y_1, \dots, y_n)^\top$is the response vector, $X_M$is the design matrix implied by model structure M, $\boldsymbol{\beta}_M$is the corresponding coefficient vector, and $\boldsymbol{\varepsilon}$ denotes the residual errors. Assuming independent Gaussian residuals, the likelihood becomes:

$$p(D \mid \beta_M, \sigma^2, M) = \prod_{i=1}^{n} N(y_i; X_M(t_i)\beta_M, \sigma^2) \tag{3}$$

Equivalently, in vector form:

$$y \mid \beta_M, \sigma^2, M \sim N(X_M \beta_M, \sigma^2 I_n) \tag{4}$$

The joint prior distribution is factorized as:

$$\pi(\beta_M, \sigma^2, M) = \pi(\beta_M, \sigma^2 \mid M)\pi(M) \tag{5}$$

such that the parameter prior and the structural prior can be specified separately. In the absence of strong prior information, weakly informative priors were adopted. Conditional on model structure M, a normal–inverse-gamma prior was assigned to $(\boldsymbol{\beta}_M, \sigma^2)$:

$$\pi(\beta_M, \sigma^2 \mid M) = \pi_\beta(\beta_M \mid \sigma^2, \nu, M)\pi_{\sigma^2}(\sigma^2 \mid a, b), \tag{6}$$

With $\pi_\beta(\beta_M \mid \sigma^2, \nu, M) = N(\beta_M; 0_M, \sigma^2 \nu I_M),$ (7)

And $\pi_{\sigma^2}(\sigma^2 \mid a, b) = IG(\sigma^2; a, b)$ (8)

Here, $\mathbf{0}_M$ is a zero vector and I_M is an identity matrix whose dimension depends on the model structure M. The scalar ν controls the prior dispersion of the regression coefficients. Because suitable fixed values for ν are generally unavailable a priori, ν was also treated as unknown and assigned an inverse-gamma hyperprior:

$$\pi_\nu(\nu \mid c, d) = IG(\nu; c, d) \tag{9}$$

Accordingly, the hierarchical prior for the model parameters becomes:

$$\pi(\beta_M, \sigma^2, \nu \mid M; a, b, c, d) = \pi_{\beta_M}(\beta_M \mid \sigma^2, \nu, M)\pi_{\sigma^2}(\sigma^2 \mid a, b)\pi_\nu(\nu \mid c, d) \tag{10}$$

The structural prior $\pi(M)$is specified to be intentionally vague, reflecting limited prior knowledge regarding the number and timing of abrupt changes in the observed signal. Because BEAST parameterizes the trend and seasonal components separately, the model prior is expressed as:

$$\pi(M) = \pi(\{m\} \cup \{\tau_j\}_{j=1,\dots,m})\pi(\{p\} \cup \{\xi_k\}_{k=1,\dots,p} \cup \{L_k\}_{k=0,\dots,p}) \tag{11}$$

where $m$and $\{\tau_j\}$denote the number and locations of trend changepoints, respectively; $p$and $\{\xi_k\}$denote the number and locations of seasonal changepoints; and $L_k$denotes the harmonic order within each seasonal segment. For the trend component, the prior is decomposed as:

$$\pi(\{m\} \cup \{\tau_j\}_{j=1,\dots,m}) = \pi(\{\tau_j\}_{j=1,\dots,m} \mid m)\pi(m) \tag{12}$$

The number of trend changepoints is assigned a discrete uniform prior over the admissible range $\{0,1,\ldots,M_{tcp}\}$:

$$\pi(m) = \begin{cases} \frac{1}{M_{tcp}+1}, \text{ if } 0 \le m \le M_{tcp} \\ 0, \text{ if m} > M_{tcp} \end{cases} \tag{13}$$

Conditional on m, the changepoint locations are assumed to lie on the set of observation times $\{t_i\}_{i=1}^{n}$, subject to a minimum separation constraint h_{tcp} to avoid implausibly dense changepoint configurations. A coherent formulation is therefore:

$$\pi(\{\tau_j\}_{j=1,\ldots,m} | m) \propto 1(\min_{i \ne j} | \tau_i - \tau_j | \ge h_{tcp}) \tag{14}$$

where $\mathbf{1}(\cdot)$ is the indicator function. If changepoints are ordered, the same condition may equivalently be written as:

$$\pi(\{\tau_j\}_{j=1,\ldots,m} | m) \propto 1(\tau_j - \tau_{j-1} \ge h_{tcp}, \forall j = 2,\ldots,m) \tag{15}$$

This part is important: the formula visible in your screenshot is not reliable enough to use as-is. The version above is the one that is logically consistent with the stated minimum-spacing assumption. The seasonal structural prior is specified analogously:

$$\pi(\{p\} \cup \{\xi_k\}_{k=1,\ldots,p} \cup \{L_k\}_{k=0,\ldots,p}) = \pi(\{\xi_k\}_{k=1,\ldots,p} | p)\pi(p)\prod_{k=0}^{p}\pi(L_k) \tag{16}$$

The number of seasonal changepoints $p$is assigned the same form of prior as the trend component, but with an upper bound M_{scp}:

$$\pi(p) = \begin{cases} \frac{1}{M_{tcp}+1}, \text{ if } 0 \le p \le M_{tcp} \\ 0, \text{ if p} > M_{tcp} \end{cases} \tag{17}$$

Similarly, the seasonal changepoint locations satisfy a minimum separation constraint h_{scp}:

$$\pi(\{\xi_k\}_{k=1,\ldots,p} | p) \propto 1(\min_{r \ne s} | \xi_r - \xi_s | \ge h_{scp}) \tag{18}$$

The harmonic order in each seasonal segment, L_k, is assigned a discrete uniform prior over the allowable interval $[L_{\min}, L_{\max}]$:

$$\pi(L_k) = \begin{cases} \frac{1}{L_{\max} - L_{\min} + 1}, \text{ if } L_{\max} \le \mathrm{L_k} \le L_{\min} \\ 0, \textit{ otherwise} \end{cases} \tag{19}$$

By combining the likelihood and prior components, the full posterior distribution can be written as:

$$p(\beta_M, \sigma^2, \nu, M \mid D) \propto p(\beta_M, \sigma^2, M \mid D)\ \pi_\beta(\beta_M \mid \sigma^2, \nu, M)\pi_{\sigma^2}(\sigma^2 \mid a, b)\pi_\nu(\nu \mid c, d)\pi(M) \qquad (20)$$

Expanding the structural prior yields:

$$p(\beta_M, \sigma^2, \nu, M \mid D) \propto \prod_{i=1}^{n} N(y_i; X_M(t_i)\beta_M, \sigma^2)\ \pi_\beta(\beta_M \mid \sigma^2, \nu, M)\pi_{\sigma^2}(\sigma^2 \mid a, b)\pi_\nu(\nu \mid c, d)$$
$$\pi(\{\tau_j\}_{j=1,\ldots,m}, m \mid \mathrm{M_{tcp}}, \mathrm{h_{tcp}})\pi(\{\xi_k\}_{k=1,\ldots,p} \mid p)\pi(p)\prod_{k=0}^{p}\pi(L_k \mid p, \mathrm{L_{min}}, \mathrm{L_{max}}) \qquad (21)$$

Under the conjugate hierarchical structure, posterior sampling can be conducted through a hybrid Gibbs–MCMC scheme. The posterior can be factorized into three principal conditional distributions. First, the conditional posterior of the model structure is:

$$p(M \mid \nu, D) \propto p(D \mid \nu, M)\pi(M) \qquad (22)$$

where $p(\mathcal{D} \mid \nu, M)$ denotes the marginal likelihood after integrating out $\boldsymbol{\beta}_M$ and σ^2.

Second, the joint conditional posterior of $(\boldsymbol{\beta}_M, \sigma^2)$ is:

$$p(\beta_M, \sigma^2 \mid \nu, M, D) = N(\beta_M; V_M^* X_M^T y, \sigma^2 V_M^*) IG(\sigma^2; a + \frac{n}{2}, b + [y^T y - y^T X_M V_M^* X_M^T y])$$

With

$$V_M^* = (\nu^{-1} I_M + X_M^T X_M)^{-1} \qquad (23)$$

Third, the conditional posterior of ν is:

$$p(\nu \mid \beta_M, \sigma^2, M, D) = IG\left(\nu; c + \frac{p_M}{2}, d + \frac{\sum_{k=1}^{p_M} \beta_{k,M}^2}{2}\right) \qquad (24)$$

where p_M denotes the total number of coefficients across all linear and harmonic segments under structure M. These conditional posterior distributions are sampled iteratively to generate a Markov chain of posterior realizations, from which changepoint probabilities, trend estimates, seasonal components, and associated uncertainty measures can be derived."

All parameter settings follow published implementation guidance for the BEAST framework, and the publicly available code is referenced in the manuscript.

1.6- Handling of serial correlation and seasonality is not fully specified, and Sen's slopes are reported in percent without clearly defining the reference quantity, hindering physical interpretation.

Response: We thank the reviewer for this important and constructive comment. We agree that the original version did not sufficiently specify how seasonality and serial correlation were handled before trend estimation. To address this issue, we added a detailed operational description in Section 2.1, Operational description, of the Supplementary Data.

Specifically, because the GWS, RZSM, and SSM time series are weekly and may contain both seasonal cycles and temporal persistence, we first removed the seasonal component using weekly climatology. For each variable and region, the long-term mean value for each week of the year was calculated across the full study period and subtracted from the corresponding weekly observations. This produced deseasonalized anomaly series and prevented the regular intra-annual cycle from being interpreted as a long-term monotonic trend.

We then assessed serial correlation using the autocorrelation function of the deseasonalized and detrended residual series for lags from 1 to 52 weeks. Autocorrelation coefficients exceeding $\pm 1.96/\sqrt{N}$ were considered statistically significant at the approximate 95% confidence level. Where significant lag-1 autocorrelation was detected, a trend-free pre-whitening procedure was applied before estimating the final trend magnitude. Sen's slope was subsequently calculated from the deseasonalized and serial-correlation-adjusted series.

We also revised the reporting of Sen's slopes to avoid ambiguity in physical interpretation. In the original version, the slopes were reported in percent, which could be misleading because the analyzed GWS, RZSM, and SSM variables are percentile-based indicators on a 0–100 scale. Therefore, the revised manuscript now reports Sen's slopes as percentile points yr^{-1}, not as simple percentage changes. This clarification makes clear that the slopes represent temporal changes in percentile-based drought indicators, rather than direct volumetric changes in groundwater storage or soil moisture.

We added the following explanation in Section 2.1, Operational description, of the Supplementary Data:

"Because the GWS, RZSM, and SSM time series are weekly and may contain both seasonal cycles and temporal persistence, trend estimation was performed after explicitly accounting for seasonality and serial correlation. First, the seasonal component was removed using weekly climatology. For each variable and region, the long-term mean for each week of the year was calculated across the full study period and subtracted from the corresponding weekly observations. This produced deseasonalized anomaly series and prevented the regular intra-annual cycle from being interpreted as a long-term trend. Second, serial correlation was assessed using the autocorrelation function of the deseasonalized and detrended residual series for lags from 1 to 52 weeks. Autocorrelation coefficients exceeding $\pm 1.96/\sqrt{N}$ were considered statistically significant at the approximate 95% confidence level. Where significant lag-1 autocorrelation was detected, a

trend-free pre-whitening procedure was applied before estimating the final trend magnitude. Sen's slope was then calculated from the deseasonalized and serial-correlation-adjusted series."

In addition, Table 2 has been revised accordingly to:

Table 1. Trend analysis results: bold values indicate trends significant at 95% and 99% confidence levels across five countries in North Africa.

Variables	Country Name	Sen' slope	Direction	Significant at 5%	Significant at 1%
Surface soil moisture percentile (SFSM)	Egypt	-0.00675	Decreasing	FALSE	FALSE
	Morocco	-0.34094	Decreasing	TRUE	TRUE
	Algeria	-1.75262	Decreasing	TRUE	TRUE
	Libya	-1.98698	Decreasing	TRUE	TRUE
	Tunisia	-2.03501	Decreasing	TRUE	TRUE
Root zone soil moisture percentile (RZSM)	Egipt	-0.18387	Decreasing	TRUE	FALSE
	Morocco	-0.3635	Decreasing	TRUE	TRUE
	Algria	-2.0268	Decreasing	TRUE	TRUE
	Libya	-2.21761	Decreasing	TRUE	TRUE
	Tunisia	-2.27731	Decreasing	TRUE	TRUE
Groundwater storage percentile (GWS)	Egipt	-0.23679	Decreasing	TRUE	TRUE
	Morocco	-0.57408	Decreasing	TRUE	TRUE
	Libya	-1.90643	Decreasing	TRUE	TRUE
	Algria	-2.15695	Decreasing	TRUE	TRUE
	Tunisia	-2.91261	Decreasing	TRUE	TRUE

1.7- Attribution of Tunisia's groundwater decline to human pumping relies mainly on GRACE-based indicators without direct abstraction, well, land-use, or socio-economic data, so causality is inferred rather than demonstrated.

Response: We thank the reviewer for highlighting this important issue. As clearly indicated in the title, the focus of this study is the analysis of spatiotemporal hydrometeorological drought in North Africa, rather than the direct quantification of groundwater abstraction.

The important observation that Tunisia's groundwater storage has declined sharply is highlighted as an outcome of the hydrological drought analysis conducted across all North African countries. We agree with the reviewer that attributing Tunisia's groundwater decline to human pumping relies primarily on GRACE-based indicators and does not incorporate direct abstraction records, well data, land-use information, or socio-economic datasets. Therefore, causality is inferred rather than formally demonstrated. Accordingly, the manuscript has been written on this basis. We explicitly state that groundwater pumping is presented as a plausible socio-hydrological driver consistent with the observed timing, rather than as a proven causal mechanism. The conclusion is interpreted as an inference supported by temporal correspondence and spatial coherence among hydrological drought-related indicators, while explicitly acknowledging that dedicated socio-hydrological

datasets would be required for formal causal attribution. This point has been clearly addressed in both the Uncertainty and Limitations section and the Conclusion.

Section 4 – Uncertainty and Limitations:

"The spatiotemporal hydrometeorological drought results reveal pronounced changes after 2010, suggesting potential links between drought intensification and anthropogenic drivers such as groundwater abstraction. However, despite the emphasis on anthropogenic influences, this study is strictly confined to hydrological and hydroclimatic evidence. Accordingly, these interpretations should be approached with caution. The limitations of the present research highlight the need for future studies to adopt integrated socio-hydrological approaches that examine groundwater governance, land-use change, and water consumption, particularly agricultural water use, in Tunisia, where the most pronounced post-2010 anomalies were observed."

Section 5 – Conclusion

"In Tunisia, for example, the spatiotemporal evolution of hydrological indicators does not align with meteorological drought trends. The results show a statistically significant decline in hydrological variables, particularly groundwater storage after 2010–2011. This contrasts with the period from 2002 to 2010, during which precipitation levels remained relatively stable and no severe meteorological drought was detected. Despite this apparent meteorological stability, a pronounced hydrological drought emerged after 2010, accompanied by a sharp decline in Tunisia's groundwater resources. This temporal decoupling suggests that additional non-meteorological factors may have contributed to the observed groundwater depletion. While excessive groundwater abstraction represents a plausible mechanism consistent with the timing and spatial coherence of the decline, formal causal attribution would require dedicated socio-hydrological data beyond the scope of the present study."

If there is any specific part of the manuscript that the reviewer considers problematic, we would be grateful for clarification so that we can revise it accordingly.

1.8- The manuscript alternates between localised pixel-scale changes and claims of sustained regional drying without a clear methodological bridge linking the two scales.

Response: During pixel-scale analyses, results were evaluated at the individual grid-cell level. In contrast, for regional- or country-scale analyses, spatial averaging was performed using the corresponding shapefiles for each region or country, applied to the raster datasets. The spatial aggregation was conducted based on nearest-neighbor resampling methods to ensure consistency between raster resolution and administrative boundaries.

We acknowledge that such scale transitions may introduce uncertainty. Accordingly, this issue has been explicitly addressed in the Uncertainty and Limitations section, where we state:

“Furthermore, the analyses combined large-scale spatial averaging with pixel-wise statistical evaluations, each of which introduces distinct analytical constraints. In particular, pixel-level changepoint detection across large spatial domains may generate statistical uncertainties and spatial artifacts, especially in regions characterized by heterogeneous hydroclimatic conditions. These limitations were anticipated and are explicitly acknowledged within the uncertainty framework of this study.”

1.9- The term “amplified drought” is central to interpretation but lacks a formal operational definition or quantitative criteria.

Response: We fully agree that Amplified Drought represents newly introduced terminology derived from a more specific component within the broader concept of anthropogenic drought. Accordingly, we have expanded the methodological explanation to clarify this framework:

“Here, we introduce an operational framework defining a specific form of anthropogenic drought, which we term Amplified Drought. Amplified Drought refers to situations in which drought categories initiated by precipitation deficits relative to long-term conditions evolve into progressively intensified impacts across subsequent drought classifications (e.g., hydrological, agricultural, and economic), varying across locations and underlying drivers. In other words, a similar meteorological drought signal, arising from reduced precipitation over a broad regional extent, may produce divergent and disproportionately amplified responses in hydrological drought indicators within specific countries or regions. Regardless of the underlying mechanisms driving such amplification, quantifying these differential responses is critically important and plays a fundamental role in water resources management. In this context, the present study proposes a time-series–based methodological framework grounded in spatial comparisons at both regional and national scales. Specifically, the temporal evolution of meteorological and hydrological drought indices was analyzed at the country level using multiple independent databases across NA a region characterized by substantial data limitations. Detailed methodological descriptions of the spatiotemporal processing and multi-source dataset analyses conducted at different spatial scales are provided below.”

1.10- Abstract and conclusions include strong quantitative and attributional statements without a clear explanation of how aggregate metrics were computed or how uncertainty was assessed.

Response: We have carefully considered this issue. If there are specific quantitative or attributional statements in the Abstract or Conclusion that you consider insufficiently supported or insufficiently explained, we would be grateful if you could indicate them in detail so that we can revise, clarify, or remove them accordingly.

1.11- Placing the legend inside the figure affects the aesthetic balance of the map; moving it outside the mapped area would enhance readability and visual quality

Response: Thank you for this suggestion. The legend has been repositioned outside the mapped area to improve readability and visual balance.

1.12- Minor but systematic presentation issues. Typos and inconsistent terminology reduce clarity, though they do not affect the core results (e.g., IMMERG vs IMERG in the Fig. 3 caption; ERA5 vs ERA5-Land; change point vs changepoint/breakpoint).Hargreaves metho" (typo) in the SPEI methods description (L170)

Response: We thank the reviewer for pointing out these inconsistencies. All typographical errors and terminology inconsistencies have been carefully corrected throughout the manuscript.

2) Reviewer #2:

I have reviewed the revised manuscript and the authors responses. I appreciate the efforts made to address the concerns raised during the earlier review. The revisions have improved the clarity and quality of the manuscript, and the authors have adequately responded to the comments and suggestions.

In my opinion, the concerns raised in the previous review have been satisfactorily addressed. Therefore, I am happy to recommend the manuscript for publication in its current form.

Communication Earth & Environment

Rebuttal Letter-4 (COMMSENV-25-3877D) for

Spatiotemporal hydrometeorological drought variations in North Africa: indications of anthropogenic contributions to amplify drought

Morteza Rahimpour[1*], Taha B.M.J. Ouarda[1], Emna Gargouri-Ellouze[2], Fairouz Slama[2]

[1]Canada Research Chair in Statistical Hydro-Climatology, Institut National de la Recherche Scientifique, Centre Eau Terre Environnement, INRS-ETE, Québec, QC, Canada. Email: Morteza.Rahimpour@inrs.ca

[2]University of Tunis El Manar, National Engineering School of TUNIS (ENIT), LR99ES19; Laboratory of Modelling in Hydraulics and Environment (LMHE), Tunis, Tunisia.

Reviewers' Responses to Questions (4):

1) Reviewer #1:

1.1- Title: Please note that manuscript titles should be 15 words or less, written in the English language, and should not contain punctuation (including colons and dashes), idioms, or puns (these do not translate well internationally).

1.2- Title: Suggested new manuscript title: Hydrometeorological drought intensified and became more persistent across North Africa between 1998 and 2024.

Response 1 and 2: The original title was carefully considered by the authors and was retained in the revised manuscript because we believe it most clearly reflects the central message and scope of the study. The colon was used intentionally to improve clarity by separating the broad topic from the specific focus of the manuscript.

Nevertheless, we understand the journal's title requirements. If the journal cannot allow an exception to this policy, we propose the following alternative title:

"Spatiotemporal Hydrometeorological Drought Variations in North Africa Indications of Anthropogenic Contributions to Amplified Drought"

1.3- Abstract: The abstract should be accessible to non-specialists and less than ~160 words long. It should include the background and context of the work, "Here we show" or an equivalent phrase, and then the major results and conclusions of the paper in the present tense. It must not Abstract contain references. Acronyms and element symbols should be avoided.

Response: The Abstract was carefully written to present the background, main findings, and broader implications of the study as clearly as possible. We respectfully prefer to retain the original Abstract, as we believe it more fully conveys the scope and significance of the manuscript. However, we understand the journal's editorial requirements. If the journal cannot allow the

original Abstract to be retained, we propose the following revised version, which is shortened to fewer than approximately 160 words:

"This study addresses this gap by examining multiple large-scale drought types through a streamlined, integrative framework for monitoring severe drought events driven by both climate change and human activities across North Africa, a region characterized by pronounced data scarcity and observation gaps. Here we show that spatiotemporal hydro-meteorological drought variations across North Africa indicate that anthropogenic factors seem to have substantially intensified hydrological drought conditions throughout the region. Furthermore, between 2021 and 2024, the area experienced unprecedented meteorological droughts. Evaluation of multiple independent datasets shows that traditional univariate monitoring frameworks significantly underestimate the magnitude and persistence of extreme events, such as the 2010 North African drought, because they fail to capture the multidimensional and interactive nature of drought dynamics. In Tunisia, hydrological drought patterns diverge notably from meteorological trends: despite relatively stable precipitation between 2002 and 2010, groundwater storage declined sharply after 2010–2011. This divergence underscores the potential influence of anthropogenic stressors, particularly unsustainable groundwater abstraction, in amplifying and prolonging hydrological drought beyond climatic variability alone."

1.4- Main text: The paper should be structured as follows:

- **An "Introduction" section that provides sufficient background and rationale for the work. The final paragraph of the Introduction should summarize the major findings and conclusions of the work.**
- **A "Results" section with up to two layers of sub-headings, the first of which should appear after the 'Results' heading. This should be followed by a 'Discussion' section, with no sub-headings.**
- **A combined "Results and Discussion" section could be used instead, with sub-headings.**
- **You may wish to include a "Conclusions" section at the end, with heading.**
- **A "Methods" section after the Discussion or Conclusions. Sufficient detail should be given to allow the work to be reproduced. If the Methods are central to the study, this section can be placed in between the Introduction and the Results (or Results and Discussion) section.**
- **Section numbers are not supported by our formatting style. Please make sure not to reference sections by their number in the text (or the Supplementary Information).**

Response: Done. These formatting requirements had already been considered during manuscript preparation. In response to this comment, we carefully rechecked the structure of the manuscript and confirmed that it complies with the journal's requirements. The manuscript includes an

Introduction section that provides the background and rationale for the work, with the final paragraph summarizing the major findings and conclusions. The main text has been structured according to the journal-supported format, with appropriate section headings and sub-headings. The Methods section has also been reviewed to ensure that sufficient detail is provided for reproducibility. Section numbering has been removed throughout the manuscript and Supplementary Information, and references to numbered sections have been revised or removed where applicable.

1.5- Main text: Language such as “new”, “novel”, “for the first time”, “unprecedented”, etc, should be avoided, or qualified with “to the best of our knowledge” or similar. Novelty should be made clear from the context.

Response: The manuscript has been carefully reviewed in response to this comment. Terms implying novelty, priority, or strong claims were checked throughout the text.

The term “new” appears once in the following sentence: “Furthermore, Fig. 7 and Fig. S2 illustrate that the CSR and JPL products yield different results, and averaging their outputs to create a new product is not advisable.” This use refers only to the creation of a merged data product and does not represent a novelty or priority claim regarding the study.

The term “novel” appeared once in the following sentence: “The findings indicate notable changes in the time series trends of meteorological variables within localized areas of limited spatial extent. These changes may be attributed to drought or climate change, as suggested in previous studies on NA 29,49. However, our analysis of drought impacts on groundwater resources offers a novel perspective.” The term “novel” has been replaced with wording referring to “an additional perspective” to avoid an unsupported novelty claim while preserving the intended meaning.

The phrase “for the first time” appeared once in the following sentence: “These challenges, particularly in the aftermath of the unprecedented emerging drought (2021–2024) documented here for the first time, are expected to have profound and far-reaching consequences across all sectors of society. Marginalized farmers, whose livelihoods depend directly on groundwater availability, are likely to be the most severely affected59.” This phrase has been removed to avoid an explicit priority claim.

The term “unprecedented” has been retained where it is central to the main message of the manuscript and to the interpretation of the amplified drought conditions documented in this study.

1.6- Display items: For all graphs depicting a single point value (e.g., mean) with error bars, you must add individual data points or convert the graph to a boxplot or dot-plot. This applies to bar graphs and line graphs. You may wish to refer to this blog post about representing data distribution in plots (particularly for small datasets). https://ecrlife420999811.wordpress.com/2018/07/10/beyond-bar-graphs-free-tools-and-resources-for-creating-more-transparent-figures-for-small-datasets/ . We strongly encourage the same for plots with multiple time courses depicted.See the Comms Bio Editorial (June 2019) for more details about this policy. Example plots are shown here: https://www.nature.com/articles/s42003-019-0489-0

Response: The figures have been carefully reviewed in response to this comment. The main figures in the manuscript primarily present spatial patterns, temporal variations, and drought-index time series rather than bar graphs or line graphs depicting a single point value, such as a mean with error bars. Therefore, this requirement is not directly applicable to most of the display items in the manuscript.

Display items: Using rainbow colour plots can be visually misleading (see links below). Please change the colour schemes used in your plots to perceptually uniform ones.

https://www.nature.com/articles/s41467-020-19160-7

https://www.climate-lab-book.ac.uk/2014/end-of-the-rainbow/

https://root.cern.ch/rainbow-color-map

Response: The colour schemes used in the figures were carefully selected during figure preparation to avoid visually misleading rainbow-style colour maps. In response to this comment, we reviewed all figure files again and confirmed that the current colour schemes are simple, visually interpretable, and appropriate for the spatial and temporal gradients presented in the manuscript. Accordingly, the figure colour schemes were retained. We would, of course, be pleased to make any further adjustments if the editorial team identifies a specific figure or colour scale that requires revision.

Source Data: All source data, i.e. the numerical results underlying the graphs and charts presented in the main figures,are mandatory and must be made available as Supplementary Data (in Excel or text format) or via a generalist repository (e.g., Figshare or Dryad).
Response: Done. The source data underlying the graphs and charts in the main figures have been compiled and provided as Supplementary Data files in Excel format. These files contain the numerical results used to generate the relevant figure panels. We have also revised the Data Availability statement to clarify that the source data for the main figures are available in the accompanying Supplementary Data files.

1.7- Source Data: Please include a caption for the source data file and add a sentence to the Data Availability statement to describe how source data can be accessed using text similar to "The source data for Figure X is in Supplementary Data Y".

Response: Done. A caption has been added for the Source Data file. The Data Availability statement has also been revised to describe how the source data underlying the main figures can be accessed. Specifically, we have added the following sentence to the Data Availability statement:

"The source data for Figures 1–8 are provided in the accompanying source data file entitled "Figures Data.rar"."

1.8- Data availability:

- **At the time of publication, the minimal dataset necessary to interpret, replicate and build upon the methods or findings reported in the article should be available in a publicly accessible repository. The data should be uploaded to a repository before the manuscript is accepted. If this is not possible, for example, for legal or ethical concerns, this must be clearly stated in the Data Availability statement.**
- **The statement 'data are available upon request' or making data available solely within the paper or Supplementary Information is not acceptable, data should be placed in a publicly accessible repository.**
- **We distinguish model output from data; we encourage (but do not require) authors to deposit model output in a publicly accessible repository.**
- **All source data, i.e. the numerical results underlying the graphs and charts presented in the main figures must also be made available via a permanent repository (e.g., Figshare or Dryad). Please provide details on access in the Data Availability statement.**

Response: Done.

1.9- Data: Authors should cite any datasets stored in external repositories that are mentioned within their manuscript. For previously published datasets, we ask authors to cite both the related research articles and the datasets themselves. For more information on how to cite datasets in submitted manuscripts, please see our submission guidelines.

Response: Done. This requirement had already been addressed through a dedicated table in the manuscript entitled "Table 1. Overview of all data used in this study, including variables, data type, dataset name, available period, spatial resolution, and references." This table provides a comprehensive overview of all datasets used in the study, including more detailed information than the dataset name and access source alone, such as variables, data type, temporal coverage, spatial resolution, and references. In response to this comment, we carefully rechecked Table 1, the Data Availability statement, and the Reference list to ensure consistency and to confirm that externally

stored datasets mentioned in the manuscript are properly cited. Where applicable, both the dataset records and the related research articles have been cited.

1.10- Code availability: If code is not fully open, where code is a key advance within the paper, please provide details here of your alternative approach to describing the code advance, to make this aspect of value to the research community.

Response: Done. This requirement has already been addressed. The code used in this study is publicly available, and the corresponding access information has been provided in the revised manuscript. In addition, the methodological workflow has been described in the Methods section with sufficient detail to support reproducibility. The Code Availability statement has also been checked to ensure that the public code repository and access details are clearly reported.

1.11- Software: Please indicate the explicit version of the software used for any simulation or data analysis in the relevant point of the Methods section.

Response: Done.

1.12- Press: We regularly highlight papers published on the journal's Social Media accounts. If you would like us to mention authors, institutions, or lab groups in these posts, please provide the social media along with the respective handles in the right-hand column. Please let us know if there are any plans for an Institutional press release on your article.

Response: Thank you for the opportunity to provide social media information for potential journal promotion. At this stage, we do not have any confirmed plans for an institutional press release. If such plans are developed, we will inform the editorial team accordingly.

1.13- Featured Image: We would welcome the submission of material for the 'Featured Image' section on the journal home page. Images should relate to the content of your manuscript but need not be contained within the paper. Photographs and aesthetically interesting images are preferred; diagrams are generally not used. Suggestions should be uploaded as a Related Manuscript file. Please provide 1200x675-pixel RGB images. You will also need to submit a completed Image License to Publish. Unfortunately, we cannot promise that your suggestions will be used. Note that images should not be created using generative AI tools. In accordance with Nature Portfolio policy, we cannot use such images.

Response: Thank you for the opportunity to submit material for the Featured Image section. At this stage, we are not submitting a Featured Image.

1.14- Editorial Summary: If accepted, your paper will be accompanied by a two-sentence summary when it is published on our homepage. Please approve the draft summary below or provide us with a suitably edited version. <North Africa has experienced a persistent drying trend since 1998 culminating in unprecedented drought conditions in 2024, potentially amplified by anthropogenic contributions, according to a comparative data-driven approach integrating satellite observations, global datasets and observations.>

Response: We thank the editorial team for preparing the draft Editorial Summary. We approve the editorial summary.

2) Reviewer #2:

2.1- Files: We note that the main article file for your manuscript has been submitted in PDF format, which we are unable to accept at this stage. Please resubmit your article file in DOC, DOCX or LaTeX format instead. If you submit as a LaTeX file, please also provide a compiled PDF, which should include all reference (BIB/BBL) data.

Response: Done. The main article file has been resubmitted in an editable DOCX format. As the manuscript was prepared in Microsoft Word, no LaTeX, BIB, or BBL files are applicable.

2.2- Figures: Please upload your main Figures (1-8) as individual files. All associated components of a Figure should fit on one page, and each page should be presented in a separate file titled "Figure XX."

Response: Done. The main figures have been uploaded as separate files titled "Figure_1" to "Figure_8". Each file contains all associated panels and components for the corresponding figure on a single page, in accordance with the journal's figure-preparation guidelines.

2.3- Figures: At this stage we can only accept main text figure files (1-8) in the following formats: PSD, AI, EPC, TIFF, JPG, PDF, PS, GIF, PPT, PPTX, PNG, BMP, VSD, CDX, SVG, EMF, EPS. Please resupply your figures following these guidelines.

Response: Done.

2.4- Figures: Please ensure that all main text Figure captions (1-8) are grouped together at the end of your main manuscript file. Embedding captions within figure files is not permitted at this stage.

Response: Done. All main text figure captions for Figures 1–8 have been grouped together at the end of the main manuscript file.

2.5- Figures: Your Figure files must not include any additional text or labels that do not form part of the Figure itself. Please remove the text within the file of Figures (1-8), if any.

Response: Done.

2.6- Tables: GUIDANCE: Data in Tables must be free from bold/italic formatting unless this has been clearly defined in the footnote. Where Tables contain images, the images should appear in their own cell in the absence of any text.

Response: Done.

2.7- Titles and Captions: GUIDANCE: Figure and Table Captions: Please ensure that all Main Text Figures and Tables include both titles and captions. The titles must describe the figure as a whole and must not contain reference to specific figure panels. The titles should be brief. All figure panels must be defined in the legend. Figure legends should be informative, clear, not unnecessarily long.

Response: Done. All main text figure and table captions have been reviewed.

2.8- Titles and Captions: We notice the Figure 4, panel a, b, c, d, e, f, g, h are not defined in the legend. Please ensure that all figure panels are defined.

Response: Figure 4 and its legend have been reviewed in response to this comment. In response to a reviewer's request during the previous revision stages, the station names and station codes were included directly within the figure panels to ensure that each station can be clearly identified. Because this information is already shown within the figure itself, and to avoid unnecessary repetition and an overly long caption, we prefer to retain the concise version of the caption. However, if defining all panels explicitly in the legend is required according to the journal's formatting style, we propose the following revised caption:

"Fig. 4. Twelve-month SPI (SPI-12; black bars) and precipitation (blue line) patterns at eight selected observation stations in North Africa during 1984–2024: a, SKIKDA (AGM00060355); b, MOSTAGANEM (AGM00060457); c, BASSATINE (MOM00060150); d, MENARA (MOM00060230); e, JENDOUBA (TS000060725); f, GAFSA (TSM00060745); g, KAIROUAN (TSE00147773); and h, CARTHAGE (TSM00060715)."

2.9- Supplementary Information: Please ensure any highlighting or tracked changes are removed from the Supplementary Information file at this stage as it will be published as it appears on the system.

Response: Done. The Supplementary Information file has been carefully reviewed, and all highlighting, tracked changes, comments, and mark-ups have been removed. The uploaded Supplementary Information file is a clean version suitable for publication as it appears in the submission system.

2.10- Supplementary Information: Please supply your Supplementary Information file in PDF format.

Response: Done.

2.11- Main Text GUIDANCE: Please check that you have not copied any text directly from published work (even your own) without clear attribution, including one or more references. We run a plagiarism detection software and may need to request additional changes if we identify large blocks of identical text.

Response: Done.

2.12- ORCID:

We are committed to improving transparency in authorship. As part of our efforts in this direction, we are now requesting that all authors identified as 'corresponding author' create and link their Open Researcher and Contributor Identifier (ORCID) with their account on the Manuscript Tracking System (MTS) prior to acceptance. ORCID helps the scientific community achieve unambiguous attribution of all scholarly contributions. For more information, please visit http://www.springernature.com/orcid.

For all corresponding authors listed on the manuscript (Dr Morteza Rahimpour), please follow the instructions in the link below to link your ORCID to your account on our manuscript tracking system before submitting the final version of the manuscript. If you do not yet have an ORCID you will be able to create one in minutes.

https://www.springernature.com/gp/researchers/orcid/orcid-for-nature-research

Please note that we cannot link ORCIDs on your behalf, and therefore these should not be supplied in the manuscript file.

All authors identified as 'corresponding author' on the manuscript must follow these instructions. Non-corresponding authors do not have to link their ORCIDs but are encouraged to do so. Please note that it will not be possible to add/modify ORCIDs at proof. Thus, if they wish to have their ORCID added to the paper they must also follow the above procedure prior to acceptance.

To support ORCID's aims, we only allow a single ORCID identifier to be attached to one account. If you have any issues attaching an ORCID identifier to your account on our manuscript tracking system, please contact the Platform Support Helpdesk at http://platformsupport.nature.com/

Response: Done.

2.13- Data Availability:

The Data availability statement should include:

The data types used in the study and how they can be accessed

Accession codes for deposited data

Other unique identifiers (such as DOIs and hyperlinks for any other datasets)

If applicable, a statement explaining any restrictions on data access

If data are reported in the study, the statement should specify, at a minimum, that all relevant data are available from the authors upon request. This statement should include details on who will be responsible for replying to this request.

If no data were used or generated in the study, the Data Availability statement should be as follows: 'Data sharing not applicable to this article as no datasets were generated or analysed during the current study.'

You should also state how the data are currently being stored. If a dataset has a Digital Object Identifier (DOI) as its unique identifier, we ask that you include this in the Reference list and cite the dataset in the Data Availability Statement.

For data restriction (on generated data): We require the following parameters in DAS- reasons for controlled access, precise conditions of access (including contact details for access requests), a timeframe for response to requests and details of any restrictions imposed on use of controlled data by reporting conditions of data use agreements.

Response: Done. The Data Availability statement has been reviewed to ensure that it includes the required information on the data types used in the study, how the datasets can be accessed, and the relevant unique identifiers, including DOI and hyperlinks where applicable. Additionally, the datasets used in this study are publicly available and have also been summarized in Table 1, which provides the variables, data type, dataset name, available period, spatial resolution, and references.

2.14- Code Availability: We strongly encourage authors to deposit all code associated with the paper in a repository where they can be freely and enduringly accessed. Obtain a DOI and add to the reference list. Cite the reference in the text alongside the URL.

Response: Done. This requirement has already been addressed. The code used in this study is publicly available, and the corresponding access information has been provided in the revised manuscript. In addition, the methodological workflow has been described in the Methods section with sufficient detail to support reproducibility. The Code Availability statement has also been checked to ensure that the public code repository and access details are clearly reported.

2.15- Acknowledgements: Any relevant funding should be declared in a separate funding statement. Please refer to our funding statement guidelines for more information.

After making any changes, please update the manuscript tracking system with the text of your new acknowledgements statement.

Response: Done. Funding information has been declared separately in a dedicated Funding statement. The Acknowledgements section has been revised accordingly, and the updated acknowledgements text has been entered in the Manuscript Tracking System.
