# Supplementary material for: Spatiotemporal variations in hydrometeorological drought across North Africa and indications of anthropogenic amplification
Source: Commun Earth Environ. 2026 Jul 13;7(1):871. doi: 10.1038/s43247-026-03807-2 (PMC13736680; doi:10.1038/s43247-026-03807-2)
Supplement: Supplementary file 2 [file 43247_2026_3807_MOESM2_ESM.pdf]

Communication Earth & Environment

Supporting Information for

Spatiotemporal variations in hydrometeorological drought across North Africa and indications of anthropogenic amplification

Morteza Rahimpour[1*], Taha B.M.J. Ouarda[1], Emna Gargouri-Ellouze[2], Fairouz Slama[2]

[1]Canada Research Chair in Statistical Hydro-Climatology, Institut National de la Recherche Scientifique, Centre Eau Terre Environnement, INRS-ETE, Québec, QC, Canada. Email: Morteza.Rahimpour@inrs.ca

[2]University of Tunis El Manar, National Engineering School of TUNIS (ENIT), LR99ES19; Laboratory of Modelling in Hydraulics and Environment (LMHE), Tunis, Tunisia.

1 Introduction

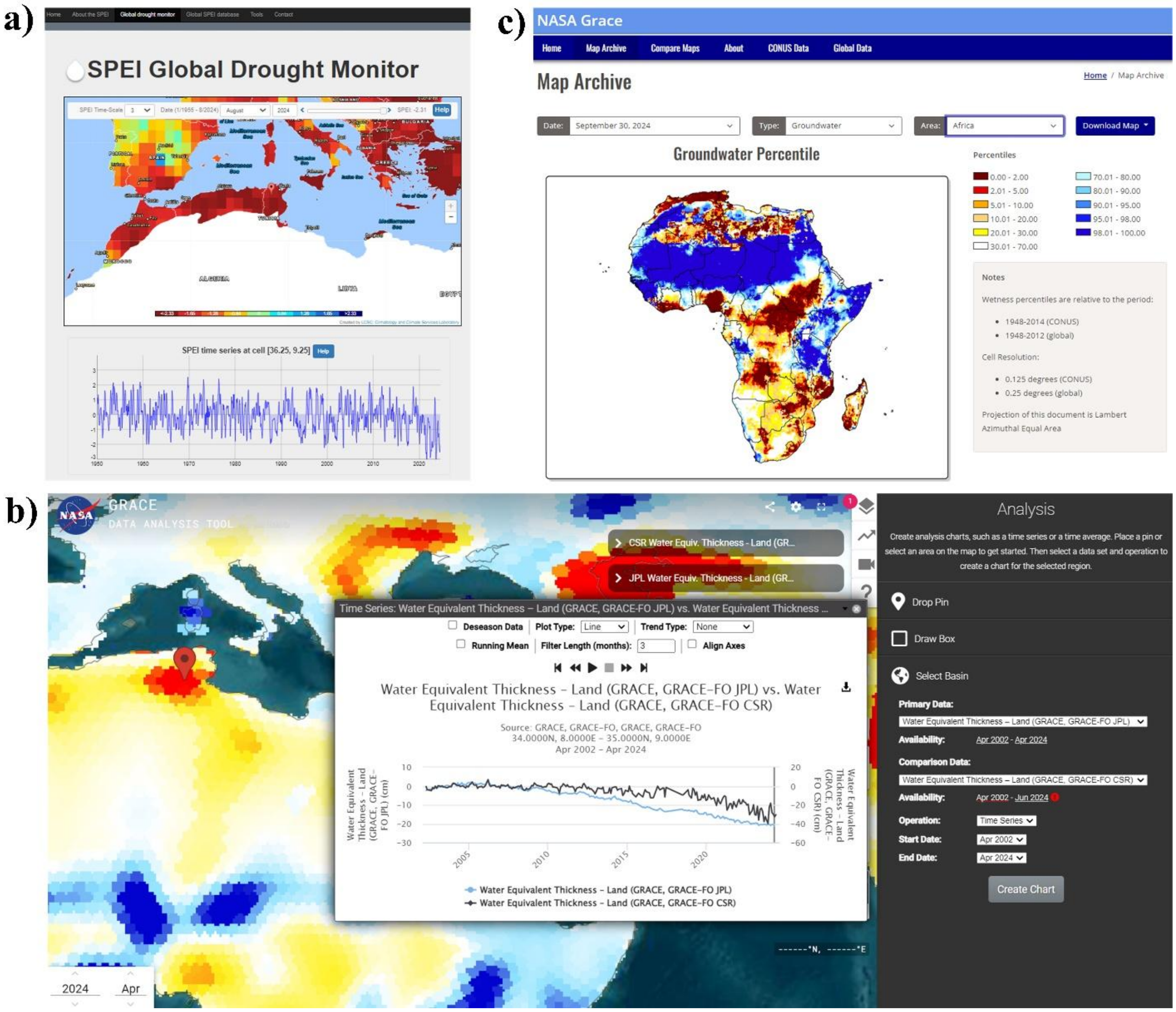

Fig.S1. Examples of three user-friendly websites that provide publicly accessible information for North Africa: a) Climatology and Climate Services Laboratory (LCSC), b) GRACE-based drought indicator product generation , c) CSR and JPL GRACE and GRACE-FO mascon ocean, ice, and hydrology equivalent water .

Table S1. Station details provided by the Global Historical Climatology Network (GHCN) for the study area (Applequist et al., 2024)

Country	GHCN_ID	Station_Name	Latitude	Longitude	Elevation (m)	First_Year	Last_Year
Algeria	AG000060390	ALGER-DAR EL BEIDA	36.72	3.25	24	1837	2025
	AG000060590	EL-GOLEA	30.57	2.87	397	1891	2024
	AG000060611	IN-AMENAS	28.05	9.63	561	1958	2025
	AG000060680	TAMANRASSET	22.80	5.43	1362	1925	2025
	AGE00135039	ORAN-HOPITAL MILITAIRE	35.73	0.65	50	1852	1966
	AGE00147704	ANNABA-CAP DE GARDE	36.97	7.79	161	1909	1937
	AGE00147705	ALGIERS-VILLE/UNIVERSITE	36.78	3.07	59	1877	1938
	AGE00147706	ALGIERS-BOUZAREAH	36.80	3.03	344	1893	1920
	AGE00147707	ALGIERS-CAP CAXINE	36.80	3.04	38	1878	1879
	AGE00147708	TIZI OUZOU	36.72	4.05	222	1879	2025
	AGE00147709	FORT NATIONAL	36.63	4.20	942	1879	1961
	AGE00147710	BEJAIA-BOUGIE (PORT)	36.75	5.10	9	1909	2011
	AGE00147712	ORLEANSVILLE (CHLEF)	36.17	1.34	112	1879	1938
	AGE00147714	ORAN-CAP FALCON	35.77	0.80	78	1875	1938
	AGE00147716	NEMOURS (GHAZAOUET)	35.10	-1.85	83	1876	2024
	AGE00147718	BISKRA	34.85	5.72	125	1845	2025
	AGE00147719	LAGHOUAT	33.80	2.89	767	1864	2024
	AGE00147720	GERYVILLE (EL-BAYADH)	33.68	1.00	1320	1888	1938
	AGM00060351	JIJEL	36.80	5.87	11	1984	2025
	AGM00060353	JIJEL-PORT	36.82	5.88	6	1989	2019
	AGM00060355	SKIKDA	36.93	6.95	7	1854	2025
	AGM00060360	ANNABA	36.82	7.81	4.9	1950	2025
	AGM00060367	EL-KALA	36.90	8.45	13	1987	2013
	AGM00060369	ALGER-PORT	36.77	3.10	12	1992	2025
	AGM00060402	SOUMMAM	36.71	5.07	6.1	1953	2025
	AGM00060403	GUELMA	36.47	7.47	228	1858	2025
	AGM00060415	AIN-BESSAM	36.32	3.53	748	1994	2025
	AGM00060417	BOUIRA	36.38	3.88	560	1950	2025
	AGM00060419	MOHAMED BOUDIAF INTL	36.28	6.62	690.4	1837	2025
	AGM00060421	OUM EL BOUAGHI	35.87	7.12	891	1988	2025
	AGM00060423	SOUK AHRAS	36.28	7.97	680	1988	2025
	AGM00060425	ECH CHELIFF	36.21	1.33	141.1	1864	2025
	AGM00060430	MILIANA	36.30	2.23	721	1865	2025
	AGM00060437	MEDEA	36.28	2.73	1036	1874	2025
	AGM00060444	BORDJ-BOU-ARRERIDJ	36.07	4.77	930	1983	2025
	AGM00060445	SETIF AIN ARNAT	36.18	5.32	1050	1855	2025
	AGM00060452	ARZEW	35.82	-0.27	4	1988	2025
	AGM00060457	MOSTAGANEM	35.88	0.12	138	1849	2025

AGM00060467	M'SILA	35.67	4.50	442	1987	2025
AGM00060468	BATNA	35.55	6.18	1052	1855	2025
AGM00060471	BARIKA	35.33	5.33	461	1990	2025
AGM00060475	CHEIKH LARBI TEBESSI	35.43	8.12	811.1	1877	2025
AGM00060476	KHENCHELLA	35.47	7.08	987	1993	2025
AGM00060490	ES SENIA	35.62	-0.62	89.9	1841	2025
AGM00060506	MASCARA-MATEMORE	35.60	0.30	476	1988	2008
AGM00060507	GHRISS	35.21	0.15	513.9	1984	2025
AGM00060511	BOU CHEKIF	35.34	1.46	989.1	1987	2025
AGM00060514	KSAR CHELLALA	35.17	2.32	801	1990	2025
AGM00060515	BOU SAADA	35.33	4.21	459	1987	2025
AGM00060518	BENI-SAF	35.30	-1.35	70	1987	2025
AGM00060520	SIDI-BEL-ABBES	35.20	-0.62	476	1859	2025
AGM00060522	MAGHNIA	34.82	-1.77	426	1950	2025
AGM00060531	ZENATA	35.02	-1.45	248.1	1853	2025
AGM00060535	DJELFA/TLETSI	34.68	3.25	1144	1875	2025
AGM00060536	SAIDA	34.87	0.15	752	1874	2025
AGM00060540	EL-KHEITER	34.15	0.07	1001	1987	2025
AGM00060549	MECHERIA	33.54	-0.24	1175	1882	2025
AGM00060550	EL-BAYADH	33.67	1.00	1347	1874	2025
AGM00060555	SIDI MAHDI	33.07	6.09	85	1908	2025
AGM00060557	NAAMA	33.27	-0.30	1166	1990	2025
AGM00060559	GUEMAR	33.51	6.78	61.9	1904	2025
AGM00060560	AIN-SEFRA	32.77	-0.60	1059	1889	2025
AGM00060563	HASSIR'MEL	32.93	3.28	764	2002	2025
AGM00060566	NOUMERAT	32.38	3.79	460.9	1884	2025
AGM00060571	BECHAR	31.65	-2.27	811.1	1906	2025
AGM00060580	OUARGLA	31.92	5.41	150	1884	2025
AGM00060581	OUED IRARA	31.67	6.14	141.1	1982	2025
AGM00060602	BENI-ABBES	30.13	-2.17	505	1903	2025
AGM00060603	RHOURD NOUSS	29.72	6.70	252	2007	2025
AGM00060607	TIMIMOUN	29.24	0.28	313	1912	2025
AGM00060620	TOUAT CHEIKH SIDI MOHAMED B	27.84	-0.19	280.1	1906	2025
AGM00060630	IN-SALAH	27.23	2.50	269	1905	2025
AGM00060640	ILLIZI TAKHAMALT	26.72	8.62	541.9	1925	2025
AGM00060656	TINDOUF	27.70	-8.17	442.9	1935	2025
AGM00060670	TISKA	24.29	9.45	968	1925	2024
AGM00060690	IN-GUEZZAM	19.57	5.77	401	1989	2025
AGMLP060354	EL MILIA	36.73	3.90	31	1990	1995
AGMLP060368	TARF	36.75	8.08	4	2024	2025
AGMLP060387	DELLYS	36.92	3.95	8	1857	2025
AGMLP060405	BOUCHEGOUF	36.50	7.72	111	2002	2025

	AGMLP060410	TENES	36.50	1.33	18	1950	2025
	AGMLP060456	MOSTAGANEM PORT	35.80	-0.08	210	1991	1995
	AGMLP060492	RELIZANE	35.43	0.35	70	1863	2023
	AGMLP060585	KRECHBA	29.07	2.21	-99	2024	2025
	AGMLP060639	TINFOUYE	28.43	7.55	471	2024	2024
	AGMLP060686	BORDJ-BADJ-MOKHTAR	21.33	0.95	399	1988	2025
	AGXLP332763	CAP CARBON	36.80	5.10	230	1951	1960
	AGXLP332768	DJIDJELI	36.50	5.43	15	1859	1892
	AGXLP332832	GHAZAOUET	35.10	-1.87	5	1991	2025
	AGXLP332910	AOULEF	27.04	1.05	290	1931	1962
	AGXLP332919	REGGANE	26.70	0.30	285	1963	1963
	AGXLP332927	OUALLEN	24.60	1.20	347	1931	1962
	AGXLP334533	BENI OUNIF	32.05	-1.14	825	1905	1980
	AGXLP334535	EL ABIOD SIDI CHEIKH	32.54	0.32	904	1909	1962
	AGXLP334537	EL ARFIANE	33.39	5.59	23	1935	1977
	AGXLP334539	FT FLATTER	28.10	6.70	381	1925	1965
	AGXLP336088	MASCARA	35.43	0.10	-999.9	1853	1961
	AGXLP336093	PUERTO	35.30	6.05	8	1907	1933
	AGXLP336128	AFLOU	34.08	2.03	1426	1874	1900
	AGXLP336129	AUMALE	36.09	3.41	894	1864	1885
	AGXLP336130	BLIDAH	36.30	2.50	260	1865	1899
	AGXLP336131	BONE	36.55	7.45	35	1858	1889
	AGXLP336132	BOUFARIK	36.43	0.30	58	1877	1900
	AGXLP336133	BOUGIE	36.49	5.03	30	1836	1884
	AGXLP336134	BOUSSAADA	35.09	1.55	669	1880	1900
	AGXLP336136	CHERCHEL	36.36	2.10	20	1843	1889
	AGXLP336139	DRAELMIZAN	36.22	3.50	780	1865	1889
	AGXLP336140	ELARICHA	34.16	-3.41	1330	1875	1889
	AGXLP336141	JEMMAPE	36.43	7.05	90	1859	1870
	AGXLP336142	LACALLE	36.54	6.06	3	1858	1889
	AGXLP336151	STAOUELI	36.43	0.35	124	1874	1889
	AGXLP336152	STDENIS	35.30	-0.12	55	1857	1880
	AGXLP336154	TENEITELHAAD	35.47	2.01	1200	1864	1889
Egypt	EG000062417	SIWA	29.20	25.32	-15	1912	2019
	EG000062432	DAKHLA	25.48	29.00	107	1932	2013
	EG000062463	HURGUADA	27.15	33.72	16	1951	2025
	EGE00147724	SALLOUM	31.55	25.18	4	1920	1990
	EGE00147726	PORT SAID	31.28	32.23	6	1880	1990
	EGE00147728	CAIRO EZBEKIYA	30.05	31.25	20	1909	1957
	EGE00147729	GIZA (CAIRO)	30.03	31.21	28	1924	1957
	EGE00147732	ISMAILIA	30.60	32.23	10	1867	1986
	EGE00147733	EL SUEZ	29.93	32.55	10	1866	1957
	EGM00062305	SALLUM PLATEAU	31.57	25.13	6	1996	2021

	EGM00062318	ALEXANDRIA INTL	31.18	29.95	-1.8	1868	2023
	EGM00062337	EL ARISH INTL	31.07	33.84	36.9	1907	2025
	EGM00062378	HELWAN	29.87	31.33	141	1904	2013
	EGM00062387	MINYA	28.08	30.73	40	1934	2013
	EGM00062423	FARAFRA	27.05	27.98	78	1957	2020
	EGM00062435	KHARGA	25.45	30.53	73	1926	2025
	EGM00062476	SHALATIN	23.13	35.58	21	2003	2025
	EGMLP062306	MERSA MATRUH	31.62	27.22	27	1905	2025
	EGMLP062366	CAIRO (AIRPORT)	30.10	31.40	74	1887	1998
	EGMLP062393	ASYOUT	27.18	31.10	23	1924	1990
	EGMLP062403	SOUTH OF WADI	26.18	32.73	78	2009	2025
	EGMLP062405	LUXOR	25.67	32.70	95	1941	1990
	EGMLP062414	ASSWAN	23.97	32.78	200	1901	2025
	EGMLP062420	BAHARIA	28.33	28.90	127	1951	2025
	EGMLP062453	EL HASANA	30.47	33.78	226	2009	2024
	EGMLP062459	TOR	28.20	33.60	3	1920	1986
	EGMLP062465	KOSSEIR	26.00	34.20	7	1927	1986
	EGMLP062467	DAHAB	28.49	34.50	19.3	2009	2025
	EGXLP333154	SIDI BARANI	31.60	26.00	23	1909	1987
	EGXLP333165	ROSETTA	31.40	30.40	3	1931	1987
	EGXLP333166	DAMIETTA	31.40	31.80	5	1931	1987
	EGXLP333174	GHAZZA	31.50	34.50	16	1953	1967
	EGXLP333175	SAKHA	31.10	30.90	15	1931	1987
	EGXLP333176	TANTA	30.80	30.90	8	1961	1986
	EGXLP333177	ZAGAZIG	30.60	31.50	13	1961	1986
	EGXLP333184	GIZA	30.00	31.20	22	1931	1986
	EGXLP333189	FAYOUM	29.30	30.80	23	1961	1986
	EGXLP333199	QENA	26.20	32.70	74	1935	1986
	EGXLP336097	GAZA	31.50	34.45	35	1923	1948
	EGXLP336098	SALUM	31.52	25.15	-999.9	1919	1950
Libya	LY000062131	HON	29.12	15.95	267	1954	2022
	LYE00147741	ZUARA	32.88	12.08	3	1920	2025
	LYE00147742	TRIPOLI SIDI EL MESRI	32.87	13.22	25	1915	2008
	LYE00147745	MISURATA	32.41	15.05	32	1914	2025
	LYE00147746	SIRTE	31.20	16.58	13	1925	2025
	LYE00147748	BENGHAZI RAGMA	32.07	20.07	322	1922	1935
	LYE00147749	AGEDABIA	30.72	20.17	7	1924	2011
	LYE00147750	SHAHAT	32.80	21.88	648	1921	2011
	LYE00147751	DERNA	32.76	22.66	10	1913	2022
	LYM00062008	YEFREN	32.08	12.55	691	1913	2025
	LYM00062012	EL KHOMS	32.63	14.30	22	1991	2025
	LYM00062062	TOBRUK	32.10	23.93	51	1914	2025
	LYM00062103	GHADAMES EAST	30.15	9.72	342	1924	2025

	LYM00062120	GARIAT EL-SHARGHIA	30.38	13.58	497	1991	2020
	LYM00062161	JALO	29.03	21.57	60	1950	2010
	LYM00062176	GIGHBUB	29.75	24.53	-1	1956	2025
	LYM00062200	OBARI	26.60	12.78	463	1993	2024
	LYM00062212	GHAT	25.15	10.14	699.8	1992	2025
	LYM00062259	TAZERBO	25.80	21.13	261	1961	2025
	LYMLP062002	NALUT	31.87	10.98	621	1913	2025
	LYMLP062010	TRIPOLI AIRPORT	32.17	13.15	81	1879	2015
	LYMLP062011	GHARIAN	32.20	13.00	721	1914	2025
	LYMLP062014	BENWLID	31.74	13.96	271	2006	2025
	LYMLP062053	BENINA	32.08	20.27	129	1881	2025
	LYMLP062124	SEBHA	27.02	14.43	432	1930	2025
	LYMLP062271	ELKUFRA	24.22	23.30	436	1933	2025
	LYW00033123	WHEELUS	32.90	13.28	13.1	1949	1967
	LYXLP333073	BENI ULID	31.80	14.00	230	1924	1989
	LYXLP333075	MIZDA	31.50	13.00	410	1914	1988
	LYXLP333086	HOMS/LA VALDAGNO	32.60	14.20	18	1913	1989
	LYXLP333103	TUMMINA	32.20	15.10	25	1951	1989
	LYXLP336100	AZIZIA	32.55	13.03	145	1919	1969
	LYXLP336103	PISIDA	33.12	11.72	10	1919	1936
	LYXLP339930	BIR EL CHINEM	32.28	12.65	150	1961	1970
	LYXLP339931	GARABULLI	32.73	13.75	40	1961	1969
	LYXLP339932	HASCIAN	32.77	12.93	50	1961	1969
	LYXLP339934	SORMAN	32.78	12.63	20	1961	1969
	LYXLP339935	TAHRIR FARM/HOMS	32.63	14.30	30	1961	1969
	LYXLP339936	THARHUNA	32.43	13.58	410	1913	1970
Morocco	MOE00147752	TANGIER CITY	35.78	-5.82	86	1912	2025
	MOE00147753	TANGIER AIRPORT	35.73	-5.90	19	1961	1978
	MOE00147754	AL HOCEIMA	35.18	-3.85	27	1965	2016
	MOM00060135	SALE	34.05	-6.75	84.1	1930	2025
	MOM00060150	BASSATINE	33.88	-5.52	576.1	1931	2025
	MOM00060155	ANFA	33.56	-7.66	61.9	1897	2025
	MOM00060220	ESSAOUIRA	31.40	-9.68	117	1872	2025
	MOM00060230	MENARA	31.61	-8.04	467.9	1886	2025
	MOM00060318	SANIAT RMEL	35.59	-5.32	3	1918	2025
	MOMLP060033	LAAYOUNE	27.17	-13.22	64	2013	2025
	MOMLP060060	SIDI IFNI	29.37	-10.18	50	1951	2025
	MOMLP060100	TANGER-PORT	35.78	-5.80	15	2001	2016
	MOMLP060101	TANGER-AERO	35.72	-5.90	15	1879	2025
	MOMLP060105	LARACHE	35.18	-6.13	47	1956	2012
	MOMLP060106	CHEFCHAOUEN	35.08	-5.30	300	2001	2025
	MOMLP060115	OUJDA	34.78	-1.93	465	1931	2025
	MOMLP060120	KENITRA	34.30	-6.60	5	1926	2025

MOMLP060127	TAZA	34.22	-4.00	509	1923	2016
MOMLP060128	TAOURIRT	34.38	-2.90	415	2001	2025
MOMLP060141	FES-SAIS	33.93	-4.98	571	2001	2025
MOMLP060146	MOHAMMEDIA	33.72	-7.40	4	2001	2012
MOMLP060160	IFRANE	33.50	-5.17	1663	1991	2025
MOMLP060165	ELJADIDA	33.23	-8.52	27	1918	2025
MOMLP060177	SETTAT	32.95	-7.62	407	1909	2012
MOMLP060178	KHOURIBGA	32.87	-6.97	770	1925	2025
MOMLP060185	SAFI	32.28	-9.23	43	1896	2025
MOMLP060190	KASBA TADLA	32.87	-6.27	507	1915	2012
MOMLP060191	BENI MELLAL	32.37	-6.40	468	2001	2012
MOMLP060195	MIDELT	32.68	-4.73	1508	1931	2025
MOMLP060200	BOUARFA	32.57	-1.95	1142	2001	2025
MOMLP060210	ERRACHIDIA	31.93	-4.40	1037	1936	2025
MOMLP060250	AGADIR INEZGANNE	30.38	-9.57	23	1921	2016
MOMLP060252	AGADIR AL MASSIRA	30.32	-9.40	74	2001	2012
MOMLP060253	TAROUDANT	30.50	-8.82	264	1931	2024
MOMLP060265	OUARZAZATE	30.93	-6.90	1136	1931	2025
MOMLP060270	TIZNIT	29.68	-9.73	260	1921	2025
MOMLP060280	GUELMIM	29.02	-10.05	300	2001	2025
MOMLP060285	TAN-TAN	28.17	-10.93	45	1948	2025
MOMLP060335	SMARA	26.67	-11.67	110	2003	2012
MOMLP060340	NADOR-AROUI	34.98	-3.02	178	2002	2024
MOW00013019	NOUASSEUR	33.37	-7.58	200.3	1951	2025
MOW00013020	SIDI SLIMANE	34.23	-6.07	55.2	1951	2012
MOW00013021	EL DJEMA SAHIM	32.35	-8.78	-999.9	1952	1953
MOW00013022	BOULHAUT	33.62	-7.12	171.3	1952	1956
MOW00013023	BEN GUERIR	32.12	-7.88	448.1	1951	1963
MOXLP332685	FES	34.02	-5.00	414	1914	1996
MOXLP334516	CAPE SPARTEL	35.80	-5.90	59	1894	1920
MOXLP334517	BEN SLIMANE	33.60	-7.20	-999.9	1913	1982
MOXLP334518	ROMMANI	33.50	-6.70	-999.9	1918	1981
MOXLP334519	AZILAL	32.00	-6.50	-999.9	1917	1981
MOXLP334520	AKNOUL	34.70	-3.60	-999.9	1932	1983
MOXLP334522	SOUK LARBAT	34.80	-6.00	-999.9	1917	1981
MOXLP334524	OUEZZENE	34.90	-5.60	-999.9	1931	1981
MOXLP334525	OUED ZEM	33.00	-6.60	-999.9	1925	1981
MOXLP334529	SIDI KACEM	34.30	-5.80	-999.9	1914	1981
MOXLP334532	DEMNATE	31.70	-7.00	-999.9	1927	1981
MOXLP336092	GRANJA AGRICOLA	35.78	-6.55	40	1917	1936
MOXLP336299	AZROU	33.27	-5.14	1665	1922	1996
MOXLP336300	BERKANE	34.59	-2.20	150	1917	1996
MOXLP336301	CAPJUBY (TARFAYA)	27.58	-12.55	-999.9	1885	1885

	MOXLP336302	ERFOUD	31.28	-4.10	815	1931	1996
	MOXLP336303	GUERCIF	34.15	-3.21	366	1922	1996
	MOXLP336304	TATA	29.44	-7.56	906	1931	1989
	MOXLP336305	TIFLET	33.54	-6.18	320	1914	1988
Tunisia	TS000060725	JENDOUBA	36.48	8.80	143	1900	2025
	TS000060765	GABES	33.88	10.10	4	1885	2025
	TSE00147767	BIZERTE CAP BLANC	37.33	9.84	264	1929	1961
	TSE00147768	BIZERTE KAROUBA	37.25	9.80	5	1886	1998
	TSE00147771	KELIBIA	36.85	11.08	29	1890	1998
	TSE00147773	KAIROUAN	35.67	10.10	60	1885	2025
	TSE00147775	SFAX EL-MAOU	34.72	10.68	21	1875	1996
	TSE00147776	TOZEUR	33.92	8.10	87	1889	1996
	TSE00147778	DJERBA MELLITA	33.87	10.77	3	1898	1992
	TSM00060715	CARTHAGE	36.85	10.23	6.7	1873	2025
	TSM00060745	GAFSA	34.42	8.82	323.1	1885	2025
	TSMLP060740	MONASTIR-SKANES	35.66	10.75	2	1971	1980
	TSMLP060775	REMADA	32.18	10.24	301	1930	1996
	TSXLP332944	AIN DRAHAM	36.48	8.42	739	1885	1996
	TSXLP332961	DJERBA	33.80	10.60	0	1890	1992
	TSXLP332965	MEDENINE	33.21	10.20	110	1904	1996
	TSXLP336379	KEBILI	33.41	8.58	36	1900	1996
	TSXLP336380	KEF	36.11	8.43	-999.9	1885	1900
	TSXLP336385	SOUK-EL-ARBA	36.30	8.47	130	1889	1900
	TSXLP336386	SOUK-EL-DJEMAA	35.54	8.55	-999.9	1885	1900
	TSXLP336387	SOUSSE	35.49	10.41	-999.9	1885	1900
	TSXLP336388	TABARKA	36.57	8.45	4	1889	1900
	TSXLP336391	ZAGHOUAN	36.25	10.08	270	1889	1900
	TSXLP340915	ZAGHONAN MAGRANE	36.43	10.77	156	1901	1998

2 Methods

2.1 Operational description

All datasets were harmonized prior to analysis to ensure spatial and temporal comparability. Spatial regridding was performed using bilinear interpolation to a common 0.25° latitude–longitude grid. For datasets with coarser native resolution, interpolation was applied conservatively to preserve large-scale variability without introducing artificial fine-scale structure.

Temporal aggregation was conducted at the monthly scale. Daily datasets (e.g., ERA5-Land) were first aggregated to monthly means (for temperature and evapotranspiration) or monthly totals (for precipitation).

A consistent land mask was applied across all datasets to ensure spatial comparability within the North Africa domain. Missing values were excluded prior to statistical analysis.

The Standardized Precipitation Index (SPI) was computed using a gamma distribution fitted to monthly precipitation totals, following standard procedures. Accumulation periods of 12 and 48 months were selected to capture interannual and multi-year drought variability.

Because the GWS, RZSM, and SSM time series are weekly and may contain both seasonal cycles and temporal persistence, trend estimation was performed after explicitly accounting for seasonality and serial correlation. First, the seasonal component was removed using weekly climatology. For each variable and region, the long-term mean for each week of the year was calculated across the full study period and subtracted from the corresponding weekly observations. This produced deseasonalized anomaly series and prevented the regular intra-annual cycle from being interpreted as a long-term trend. Second, serial correlation was assessed using the autocorrelation function of the deseasonalized and detrended residual series for lags from 1 to 52 weeks. Autocorrelation coefficients exceeding ±1.96/√N were considered statistically significant at the approximate 95% confidence level. Where significant lag-1 autocorrelation was detected, a trend-free pre-whitening procedure was applied before estimating the final trend magnitude. Sen's slope was then calculated from the deseasonalized and serial-correlation-adjusted series.

2.2 Bayesian time-series decomposition using BEAST

In a Bayesian framework, all unknown quantities are treated as random variables, including the model structure M, regression coefficients $\boldsymbol{\beta}_M$, and noise variance σ^2. For a given time, series $\mathcal{D} = \{t_i, y_i\}_{i=1,\dots,n}$, the objective is not limited to point estimation but extends to the full posterior distribution $p(\boldsymbol{\beta}_M, \sigma^2, M \mid \mathcal{D})$. According to Bayes' theorem, this posterior is proportional to the product of the likelihood and prior distributions:

$$p(\beta_M, \sigma^2, M \mid D) \propto p(D \mid \beta_M, \sigma^2, M)\pi(\beta_M, \sigma^2, M) \quad (1)$$

The observation model is expressed using the general linear representation:

$$y = X_M \beta_M + \varepsilon \quad (2)$$

where $\mathbf{y} = (y_1, \dots, y_n)^\top$ is the response vector, X_M is the design matrix implied by model structure M, $\boldsymbol{\beta}_M$ is the corresponding coefficient vector, and $\boldsymbol{\varepsilon}$ denotes the residual errors. Assuming independent Gaussian residuals, the likelihood becomes:

$$p(D \mid \beta_M, \sigma^2, M) = \prod_{i=1}^{n} N(y_i; X_M(t_i)\beta_M, \sigma^2) \quad (3)$$

Equivalently, in vector form:

$$y \mid \beta_M, \sigma^2, M \sim N(X_M \beta_M, \sigma^2 I_n) \quad (4)$$

The joint prior distribution is factorized as:

$$\pi(\beta_M, \sigma^2, M) = \pi(\beta_M, \sigma^2 \mid M)\pi(M) \quad (5)$$

such that the parameter prior and the structural prior can be specified separately. In the absence of strong prior information, weakly informative priors were adopted. Conditional on model structure M, a normal–inverse-gamma prior was assigned to $(\boldsymbol{\beta}_M, \sigma^2)$:

$$\pi(\beta_M, \sigma^2 | M) = \pi_\beta(\beta_M | \sigma^2, \nu, M)\pi_{\sigma^2}(\sigma^2 | a, b), \tag{6}$$

With $\pi_\beta(\beta_M | \sigma^2, \nu, M) = N(\beta_M; 0_M, \sigma^2 \nu I_M),$ (7)

And $\pi_{\sigma^2}(\sigma^2 | a, b) = IG(\sigma^2; a, b)$ (8)

Here, $\mathbf{0}_M$ is a zero vector and I_M is an identity matrix whose dimension depends on the model structure M. The scalar ν controls the prior dispersion of the regression coefficients. Because suitable fixed values for ν are generally unavailable a priori, ν was also treated as unknown and assigned an inverse-gamma hyperprior:

$$\pi_\nu(\nu | c, d) = IG(\nu; c, d) \tag{9}$$

Accordingly, the hierarchical prior for the model parameters becomes:

$$\pi(\beta_M, \sigma^2, \nu | M; a, b, c, d) = \pi_{\beta_M}(\beta_M | \sigma^2, \nu, M)\pi_{\sigma^2}(\sigma^2 | a, b)\pi_\nu(\nu | c, d) \tag{10}$$

The structural prior $\pi(M)$is specified to be intentionally vague, reflecting limited prior knowledge regarding the number and timing of abrupt changes in the observed signal. Because BEAST parameterizes the trend and seasonal components separately, the model prior is expressed as:

$$\pi(M) = \pi(\{m\} \cup \{\tau_j\}_{j=1,\dots,m})\pi(\{p\} \cup \{\xi_k\}_{k=1,\dots,p} \cup \{L_k\}_{k=0,\dots,p}) \tag{11}$$

where $m$and $\{\tau_j\}$denote the number and locations of trend changepoints, respectively; $p$and $\{\xi_k\}$denote the number and locations of seasonal changepoints; and $L_k$denotes the harmonic order within each seasonal segment. For the trend component, the prior is decomposed as:

$$\pi(\{m\} \cup \{\tau_j\}_{j=1,\dots,m}) = \pi(\{\tau_j\}_{j=1,\dots,m} | m)\pi(m) \tag{12}$$

The number of trend changepoints is assigned a discrete uniform prior over the admissible range $\{0, 1, \dots, M_{tcp}\}$:

$$\pi(m) = \begin{cases} \frac{1}{M_{tcp}+1}, \text{ if } 0 \le m \le M_{tcp} \\ 0, \text{ if m} > M_{tcp} \end{cases} \tag{13}$$

Conditional on m, the changepoint locations are assumed to lie on the set of observation times $\{t_i\}_{i=1}^n$, subject to a minimum separation constraint h_{tcp} to avoid implausibly dense changepoint configurations. A coherent formulation is therefore:

$$\pi(\{\tau_j\}_{j=1,\ldots,m} | \, m) \propto 1(\min_{i \neq j} | \, \tau_i - \tau_j | \geq h_{tcp}) \tag{14}$$

where $\mathbf{1}(\cdot)$ is the indicator function. If changepoints are ordered, the same condition may equivalently be written as:

$$\pi(\{\tau_j\}_{j=1,\ldots,m} | \, m) \propto 1(\tau_j - \tau_{j-1} \geq h_{tcp}, \forall j = 2, \ldots, m) \tag{15}$$

This part is important: the formula visible in your screenshot is not reliable enough to use as-is. The version above is the one that is logically consistent with the stated minimum-spacing assumption. The seasonal structural prior is specified analogously:

$$\pi(\{p\} \cup \{\xi_k\}_{k=1,\ldots,p} \cup \{L_k\}_{k=0,\ldots,p}) = \pi(\{\xi_k\}_{k=1,\ldots,p} | \, p)\pi(p) \prod_{k=0}^{p} \pi(L_k) \tag{16}$$

The number of seasonal changepoints $p$is assigned the same form of prior as the trend component, but with an upper bound M_{scp}:

$$\pi(p) = \begin{cases} \frac{1}{M_{tcp}+1}, \text{ if } 0 \leq p \leq M_{tcp} \\ 0, \text{ if } p > M_{tcp} \end{cases} \tag{17}$$

Similarly, the seasonal changepoint locations satisfy a minimum separation constraint h_{scp}:

$$\pi(\{\xi_k\}_{k=1,\ldots,p} | \, p) \propto 1(\min_{r \neq s} | \, \xi_r - \xi_s | \quad \geq h_{scp}) \tag{18}$$

The harmonic order in each seasonal segment, L_k, is assigned a discrete uniform prior over the allowable interval $[L_{\min}, L_{\max}]$:

$$\pi(L_k) = \begin{cases} \frac{1}{L_{\max} - L_{\min} + 1}, \text{ if } L_{\max} \leq \mathrm{L_k} \leq L_{\min} \\ 0, \; otherwise \end{cases} \tag{19}$$

By combining the likelihood and prior components, the full posterior distribution can be written as:

$$p(\beta_M, \sigma^2, \nu, M \mid D) \propto \; p(\beta_M, \sigma^2, M \mid D) \; \pi_\beta(\beta_M | \, \sigma^2, \nu, M) \pi_{\sigma^2}(\sigma^2 | \, a, b) \pi_\nu(\nu | \, c, d) \pi(M) \tag{20}$$

Expanding the structural prior yields:

$$\begin{aligned} p(\beta_M, \sigma^2, \nu, M \mid D) \propto \; & \prod_{i=1}^{n} N(y_i; X_M(t_i)\beta_M, \sigma^2) \; \pi_\beta(\beta_M | \, \sigma^2, \nu, M) \pi_{\sigma^2}(\sigma^2 | \, a, b) \pi_\nu(\nu | \, c, d) \\ & \pi(\{\tau_j\}_{j=1,\ldots,m}, m \mid \mathrm{M_{tcp}}, \mathrm{h_{tcp}}) \pi(\{\xi_k\}_{k=1,\ldots,p} | \, p) \pi(p) \prod_{k=0}^{p} \pi(L_k | \, p, \mathrm{L_{min}}, \mathrm{L_{max}}) \end{aligned} \tag{21}$$

Under the conjugate hierarchical structure, posterior sampling can be conducted through a hybrid Gibbs–MCMC scheme. The posterior can be factorized into three principal conditional distributions. First, the conditional posterior of the model structure is:

$$p(M \mid \nu, D) \propto p(D \mid \nu, M)\pi(M) \tag{22}$$

where $p(\mathcal{D} \mid \nu, M)$ denotes the marginal likelihood after integrating out $\boldsymbol{\beta}_M$ and σ^2.

Second, the joint conditional posterior of $(\boldsymbol{\beta}_M, \sigma^2)$ is:

$$p(\beta_M, \sigma^2 \mid \nu, M, D) = N(\beta_M; V_M^* X_M^T y, \sigma^2 V_M^*) IG(\sigma^2; a + \frac{n}{2}, b + [y^T y - y^T X_M V_M^* X_M^T y])$$

With

$$V_M^* = (\nu^{-1} I_M + X_M^T X_M)^{-1} \tag{23}$$

Third, the conditional posterior of ν is:

$$p(\nu \mid \beta_M, \sigma^2, M, D) = IG\left(\nu; c + \frac{p_M}{2}, d + \frac{\sum_{k=1}^{p_M} \beta_{k,M}^2}{2}\right) \tag{24}$$

where p_M denotes the total number of coefficients across all linear and harmonic segments under structure M. These conditional posterior distributions are sampled iteratively to generate a Markov chain of posterior realizations, from which changepoint probabilities, trend estimates, seasonal components, and associated uncertainty measures can be derived.

2.2.1 MCMC settings and convergence:

The revised text now specifies that four parallel RJ-MCMC chains were run for each pixel, each consisting of 60,000 iterations, with the first 20,000 discarded as burn-in and a thinning factor of 10. This yielded 16,000 posterior draws used for inference. Random seeds were recorded for reproducibility. Convergence was evaluated through trace plots, the Gelman–Rubin statistic ($\hat{R} \leq 1.05$), and effective sample sizes (ESS > 500). These settings are consistent with recommendations from Zhao, et al. [1],Zhao, et al. [2] and Hu, et al. [3].

2.2.2 Priors and hyperparameters:

We now include the explicit prior forms: (1) a discrete uniform prior on the number of changepoints (0–5 per layer), (2) conditional uniform priors on changepoint locations, (3) Gaussian priors with broad variances for the trend and seasonal coefficients, and (4) a weak inverse-gamma prior for the noise variance.

2.2.3 **Computation of changepoint probabilities:**

The posterior inclusion probability (PIP) for each time step is defined as the proportion of posterior samples in which that time is identified as a changepoint. This metric is directly derived from the RJ-MCMC sampling process, following Zhao, et al. [2].

2.3 Modified Mann-Kendall (MMK)

The Mann–Kendall test is a non-parametric method commonly used to detect monotonic trends in time series data [4,5]. For a dataset with N observations $d_1, d_2, \ldots d_N$, the test statistic T is defined as:

$$T = \sum_{k=1}^{N-1} \sum_{j=k+1}^{N} sign(d_j - d_k),\ sign(d_j - d_k) = \begin{cases} 1, & \text{if } d_j - d_k > 0 \\ 0, & \text{if } d_j - d_k = 0 \\ -1, & \text{if } d_j - d_k < 0 \end{cases} \tag{25}$$

The variance of T is computed as:

$$Var(T) = \frac{N(N-1)(2N+5) - \sum_{p-1}^{P} t_p(t_p - 1)(2t_p + 5)}{18} \tag{26}$$

where P is the number of tied groups and t_p is the number of observations in the pth group. The standardized test statistic Z_T is calculated as:

$$Z_T = \begin{cases} \dfrac{T-1}{\sqrt{\mathrm{Var}(T)}}, & \text{if } T > 0 \\ 0, & \text{if } T = 0 \\ \dfrac{T+1}{\sqrt{\mathrm{Var}(T)}}, & \text{if } T < 0 \end{cases} \tag{27}$$

A positive (negative) ZT indicates an increasing (decreasing) trend. The null hypothesis is rejected at the significance level α if $|ZT| > Z_{1-\alpha/2}$.

To address the effect of autocorrelation, the Modified Mann–Kendall test uses an adjusted variance based on the effective sample size N′ [6]:

$$N' = \frac{N}{1 + 2\sum_{k=1}^{m}\left(1 - \frac{k}{N}\right) r_k} \tag{28}$$

Where r_k is the autocorrelation coefficient at lag k, and m is the number of significant lags. The corrected variance Var∗(T) is computed by substituting N′ into the original variance formula. The corresponding Z_T is then recalculated as in equation 12.

2.4 Sen's Slope

To estimate the rate of change in a time series, the Sen's slope estimator calculates the slope θ_i between all combinations of data pairs [7]:

$$\theta_i = \frac{d_j - d_k}{j - k}, \quad \forall j > k \tag{29}$$

The median of the θ_i values is taken as the Sen's slope estimator θ_{med}:

$$\theta_{\text{med}} = \begin{cases} \theta_{(N+1)/2}, & \text{if } N \text{ is odd} \\ \dfrac{\theta_{N/2} + \theta_{(N/2)+1}}{2}, & \text{if } N \text{ is even} \end{cases} \tag{30}$$

where $N=n(n-1)/2$ is the total number of slope values. A positive (negative) θ_{med} indicates an increasing (decreasing) trend.

3 Result

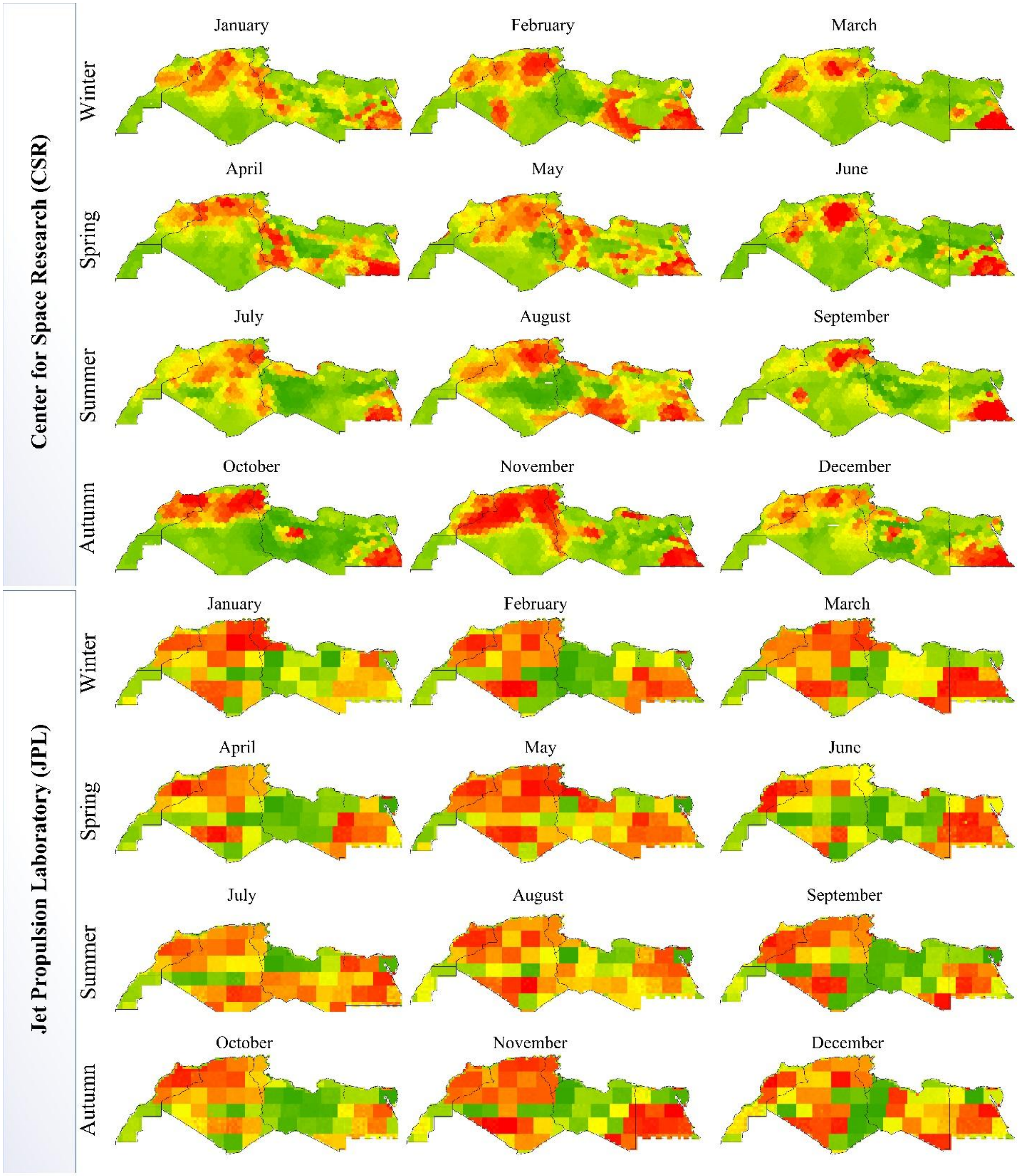

Fig.S2. Spatiotemporal pattern of monthly time series analysis results: probability of abrupt change based on two CSR and JPL GRACE products (2002–2024) across North Africa.

4 Reference

1. Zhao, K., Valle, D., Popescu, S., Zhang, X. & Mallick, B. Hyperspectral remote sensing of plant biochemistry using Bayesian model averaging with variable and band selection. *Remote Sensing of Environment* **132**, 102-119 (2013). https://doi.org/https://doi.org/10.1016/j.rse.2012.12.026
2. Zhao, K. *et al.* Detecting change-point, trend, and seasonality in satellite time series data to track abrupt changes and nonlinear dynamics: A Bayesian ensemble algorithm. *Remote Sensing of Environment* **232**, 111181 (2019). https://doi.org/https://doi.org/10.1016/j.rse.2019.04.034
3. Hu, T. *et al.* Mapping fine-scale human disturbances in a working landscape with Landsat time series on Google Earth Engine. *ISPRS Journal of Photogrammetry and Remote Sensing* **176**, 250-261 (2021). https://doi.org/https://doi.org/10.1016/j.isprsjprs.2021.04.008
4. Mann, H. B. Nonparametric tests against trend. *Econometrica: Journal of the econometric society*, 245-259 (1945).
5. Kendall, M. G. Rank correlation methods. (1948).
6. Hamed, K. H. & Rao, A. R. A modified Mann-Kendall trend test for autocorrelated data. *Journal of hydrology* **204**, 182-196 (1998).
7. Sen, P. K. Estimates of the regression coefficient based on Kendall's tau. *Journal of the American statistical association* **63**, 1379-1389 (1968).
